# A Randomized, Double-Blind, Placebo-Controlled Phase II Trial Investigating the Safety and Immunogenicity of Modified Vaccinia Ankara Smallpox Vaccine (MVA-BN®) in 56-80-Year-Old Subjects

**DOI:** 10.1371/journal.pone.0157335

**Published:** 2016-06-21

**Authors:** Richard N. Greenberg, Christine M. Hay, Jack T. Stapleton, Thomas C. Marbury, Eva Wagner, Eva Kreitmeir, Siegfried Röesch, Alfred von Krempelhuber, Philip Young, Richard Nichols, Thomas P. Meyer, Darja Schmidt, Josef Weigl, Garth Virgin, Nathaly Arndtz-Wiedemann, Paul Chaplin

**Affiliations:** 1 University of Kentucky School of Medicine, MN663 Medical Science Bldg., 800 Rose Street, Lexington, KY, 40536, United States of America; 2 University of Iowa, SW54, GH, 200 Hawkins Drive, UHC, Iowa City, IA, 52242, United States of America; 3 University of Rochester Medical Center School of Medicine and Dentistry, 601 Elmwood Avenue, Box 689, Rochester, NY, 14642, United States of America; 4 Orlando Clinical Research Center, 5055 South Orange Avenue, Orlando, FL, 32809, United States of America; 5 Bavarian Nordic GmbH, Fraunhoferstrasse 13, 82152 Martinsried, Germany; The George Washington University School of Medicine and Health Sciences, UNITED STATES

## Abstract

**Background:**

Modified Vaccinia Ankara MVA-BN^®^ is a live, highly attenuated, viral vaccine under advanced development as a non-replicating smallpox vaccine. In this Phase II trial, the safety and immunogenicity of Modified Vaccinia Ankara MVA-BN^®^ (MVA) was assessed in a 56–80 years old population.

**Methods:**

MVA with a virus titer of 1 x 10^8^ TCID_50_/dose was administered via subcutaneous injection to 56–80 year old vaccinia-experienced subjects (N = 120). Subjects received either two injections of MVA (MM group) or one injection of Placebo and one injection of MVA (PM group) four weeks apart. Safety was evaluated by assessment of adverse events (AE), focused physical exams, electrocardiogram recordings and safety laboratories. Solicited AEs consisted of a set of pre-defined expected local reactions (erythema, swelling, pain, pruritus, and induration) and systemic symptoms (body temperature, headache, myalgia, nausea and fatigue) and were recorded on a memory aid for an 8-day period following each injection. The immunogenicity of the vaccine was evaluated in terms of humoral immune responses measured with a vaccinia-specific enzyme-linked immunosorbent assay (ELISA) and a plaque reduction neutralization test (PRNT) before and at different time points after vaccination.

**Results:**

Vaccinations were well tolerated by all subjects. No serious adverse event related to MVA and no case of myopericarditis was reported. The overall incidence of unsolicited AEs was similar in both groups. For both groups immunogenicity responses two weeks after the final vaccination (i.e. Visit 4) were as follows: Seroconversion (SC) rates (doubling of titers from baseline) in vaccine specific antibody titers measured by ELISA were 83.3% in Group MM and 82.8% in Group PM (difference 0.6% with 95% exact CI [-13.8%, 15.0%]), and 90.0% for Group MM and 77.6% for Group PM measured by PRNT (difference 12.4% with 95% CI of [-1.1%, 27.0%]). Geometric mean titers (GMT) measured by ELISA two weeks after the final vaccination for Group MM were 804.1 and 605.8 for Group PM (with ratio of GMTs of 1.33 with 95% CI of [0.96, 1.84]). Similarly, GMTs measured by PRNT were 210.3 for Group MM and 126.7 for Group PM (with ratio 1.66 and 95% CI [0.95, 2.90]).

**Conclusions:**

One or two doses of MVA were safe and immunogenic in a 56–80 years old vaccinia-experienced population. No cases of myopericarditis were observed following vaccinations with MVA. The safety, reactogenicity and immunogenicity were similar to that seen in younger (18–55 year old) healthy populations as investigated in other MVA trials. The results suggest that a single dose of MVA in a 56–80 years old population was well tolerated and sufficient to rapidly boost the long-term B cell memory response induced by a prior vaccination with a traditional smallpox vaccine.

**Trial Registration:**

ClinicalTrials.gov NCT00857493

## Introduction

Following a global vaccination campaign, smallpox, caused by the variola virus (VARV), was declared eradicated in 1980 by the World Health Organization [1]. Nevertheless, concern about the re-emergence of VARV persists due to either the accidental or deliberate release of VARV stocks [2, 3]. While traditional smallpox vaccines, based on replicating vaccinia virus (VACV) strains, are highly effective, their use in a pre-emergency situation can be problematic because of rare but severe side effects associated with the replication competence of these vaccines [4, 5, 6].

MVA is a live, highly attenuated pox virus currently in advanced clinical development as a non-replicating smallpox vaccine. Due to the proven incapability to replicate in human cells [7] and based on the current database derived from completed and ongoing clinical trials [8–19], MVA is a safer alternative to traditional, replicating smallpox vaccines. MVA has been shown to be efficacious in mice [20], rabbits [21] and in non-human primates [22] challenged with species specific orthopox viruses highly related to VARV in humans. MVA has also been shown to be immunogenic with a good safety profile in healthy and at-risk populations who have contraindications for replicating smallpox vaccines, namely subjects infected with human immunodeficiency virus (HIV) [8, 9], diagnosed with atopic dermatitis (AD) [10, 17]) or under immune suppressive treatment [11]. Moreover, MVA induces peak antibody and VARV-neutralizing responses comparable to a traditional smallpox vaccine (Dryvax^®^) [12].

While trials performed to date have shown MVA is able to boost immune responses in vaccinia-experienced healthy and HIV infected subjects [8, 9, 16, 17], no data exists in older populations (>55 years old) where the majority have been vaccinated against smallpox earlier in their lives. This population is important to investigate, because one of the most striking changes that occur during normal human aging is known as immunosenescence, a progressive and overall diminution of immune functions that affects all cells and organs of the innate and adaptive immune system [23]. As a hallmark of human aging, the progressive involution of the thymus leads to an impaired balance and function of naïve, memory and effector T cells, thus promoting a latent pro-inflammatory status in the elderly. This situation manifests in clinically relevant implications such as poor overall immune responses, decreased ability to control infectious diseases and diminished response to vaccinations [12, 24, 25].

To specifically examine this older population, this trial targeted a population 56 to 80 years of age to expand the MVA clinical data in vaccinia-experienced subjects. The primary goal was to generate for the first time data on safety and immunogenicity of MVA in this population using one or two booster vaccinations.

## Methods

### Study Vaccine

The MVA strain used in the current study was derived from the MVA vaccine licensed in Germany [26, 27] by additional passages and serial dilutions in chicken embryo fibroblast cells and has been shown not to replicate in human cells or severely immunocompromised animals [7]. MVA was produced by IDT Biologika GmbH (Dessau-Roßlau, Germany) according to GMP and provided by Bavarian Nordic A/S (Kvistgaard, Denmark) in aliquots of 0.5 ml liquid-frozen vaccine containing a titer of at least 1 x 10^8^ TCID_50_.

### Study Design and Subjects

This randomized, double-blind, placebo-controlled Phase II trial conducted at four sites in the US enrolled 120 subjects. Enrollment began July 1, 2009 and the last follow-up (FU) visit was on August 24, 2010. The study was approved by the following Institutional Review Boards: University of Kentucky IRB Office of Research Integrity, 315 Kinkead Hall Lexington, KY; University of Iowa IRB Human Subjects Office, 340 College of Medicine Ad. Bldg., Iowa City, IA; Independent Investigational Review Board, 6738 West Sunrise Blvd Plantation, FL; and the Western Institutional Review Board 3535 Seventh Ave. SW Olympia, WA. Group MM received two subcutaneous (s.c.) injections with 0.5 ml MVA at week 0 and week 4. Group PM received a first injection (s.c.) with placebo (0.5 ml saline), followed by a second injection (s.c.) with 0.5 ml MVA four weeks later. Vaccinia-experienced women and men aged 56 to 80 years were eligible for enrollment. The trial consisted of a screening period of up to four weeks, an active trial period of eight to ten weeks comprised of five visits (initial vaccination, 2 week FU, second vaccination at week 4, another 2 week FU and an additional FU visit 2 weeks later) and a long term FU period of at least 26–30 weeks after the second injection ending with a final FU visit. At this final visit long-term safety data and serum for anti-vaccinia antibody analysis was collected and a physical examination was performed. Electrocardiogram and laboratory tests were to be performed only if clinically indicated. The trial was conducted according to the standards of GCP and the principles of the Declaration of Helsinki. The protocol was approved by independent review committees for each site and all subjects provided written informed consent prior to participation.

### Safety Assessments

To evaluate safety of MVA, solicited and unsolicited AEs (adverse events) were recorded at screening and at each of the five visits during the active trial phase. Serious Adverse Events (SAEs) and AEs of special interest (AESI) were monitored throughout the trial up to the six months FU visit. Solicited AEs constituted a set of pre-defined, expected local reactions (erythema, swelling, pain, pruritus, and induration) and systemic symptoms (body temperature, headache, myalgia, nausea and fatigue). All solicited AEs were recorded on a memory aid for an 8-day period following each injection. Unsolicited AEs were all events reported by the subject within a 29-day period after each injection not already solicited in the memory aid. Safety laboratory tests including troponin I and electrocardiograms (ECG) were performed at screening and two weeks after each injection. Any cardiac symptom or ECG change, which was determined to be clinically significant and elevation of Troponin I above twice the normal limit were defined as AESIs. Unsolicited or solicited AEs that prevented daily activities or body temperatures ≥ 39°C were defined as grade ≥3.

### Immunogenicity Assays

Samples for immunogenicity testing were drawn at the first visit (baseline, sample drawn prior to first vaccination) and each post-baseline visit. The vaccinia-specific humoral immune response was analyzed using a vaccinia-specific ELISA [9] and a vaccinia-specific PRNT [17]. A titer equal or above detection limit was defined as seropositive. Seroconversion was defined as a rise by a minimum of a factor of 2 from baseline [8, 9, 14].

### Study Objectives and Statistical Methods

The primary objective was to expand the MVA data base on safety in a vaccinia-experienced population 56–80 years of age after administration of either one or two doses of MVA. The secondary objectives were to investigate the safety and reactogenicity as well as to descriptively compare the humoral immune response induced by one versus two doses of MVA in this population.

The sample size calculation (two groups of 60 subjects each) is based on the primary endpoint to evaluate the incidence of SAEs. A sample size of 60 subjects provides a probability of 95% to detect SAEs with an incidence of at least 5%, i.e. 60 subjects provide a 95% certainty of detecting at least one SAE that occurs with an incidence of 1 in 20.

Subjects eligible for Group MM or PM were randomly allocated per site to one of the two treatment groups in order of their appearance in a 1:1 ratio. Randomization was performed automatically within an electronic data capture system using a block size of 8 for each of the 4 sites. The pharmacist who prepared the syringes for administration was the only individual aware of the group allocation and only had the subject number and no other subject details.

The trial was not powered to provide formal testing between arms, and the statistical analyses performed were descriptive. Confidence intervals (CI) for rates (%) were calculated using the Clopper-Pearson method, and CIs of the differences (Δ = Group MM–Group PM) in rates between groups (adverse event rates and SC rates) used a two one-sided exact confidence interval. Confidence intervals of the ratio of GMTs (Group MM/ Group PM) and fold ratios from baseline were provided by assuming that titers are log-normally distributed and calculating the standard homoscedastic t-test based interval.

## Results

### Study Population

**Fig 1:**
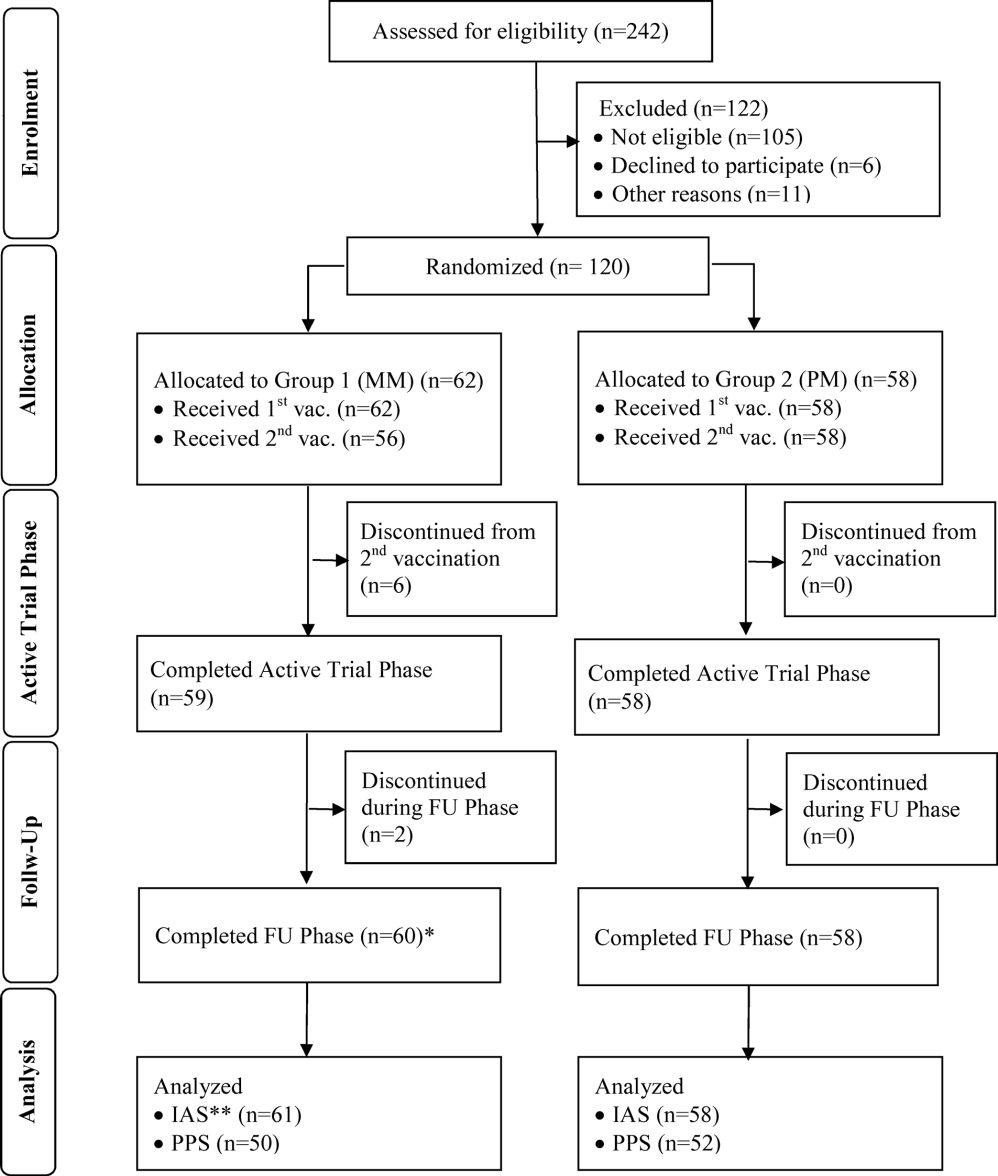
Disposition of Subjects and Data Sets analyzed. Of 242 screened volunteers, 120 subjects were assessed eligible for enrollment, allocated to one of the two groups, Group MM (MVA/MVA) or Group PM (Placebo/MVA), respectively, and received at least one vaccination of MVA. All safety data for these 120 subjects were analyzed. * One subject, who didn’t complete the active trial phase returned for the FU visit. ** One subject was excluded from the Immunogenicity Analysis Set (IAS; N = 119), because baseline results were missing. 18 subjects were excluded from the Per-Protocol Set (PPS); N = 102). FU = Follow-up.

The trial was conducted from June 2009 until August 2010 at 4 sites corresponding to the first 4 authors of this manuscript. A total of 120 subjects were enrolled. Safety data were analyzed for all enrolled subjects. One subject was excluded from the Immunogenicity Analysis Set (IAS) because baseline results were missing. 18 subjects were excluded from the Per Protocol Set (PPS, Fig 1). The results of the PPS were similar to that for the IAS. In this manuscript only the immunogenicity results obtained using the IAS are described. The analyses performed with the PPS are provided as supporting information (S1 and S2 Tables; S1 and S2 Figs).

### Demographics

There were no relevant differences observed in any demographic parameter between groups. In both groups, more female than male subjects were included (Table 1).

**Table 1:** Demographic Data (all enrolled subjects, N = 120).

		Group MM (N = 62)	Group PM (N = 58)
Age [years]	Mean (SD)	64.6 (5.4)	62.6 (5.9)
	95% CI	(63.3, 66.0)	(61.1, 64.1)
	Median	64.0	62.0
	Range	56–77	56–80
Gender [n (%)]	Female	37 (59.7)	40 (69.0)
	Male	25 (40.3)	18 (31.0)
Race [n (%)]	White (Caucasian)	59 (95.2)	57 (98.3)
	Black or African American	2 (3.2)	1 (1.7)
	Asian	1 (1.6)	0 (0.0)
Ethnicity [n (%)]	Hispanic or Latino	1 (1.6)	0 (0.0)
	Non-Hispanic or Latino	61 (98.4)	58 (100.0)

N = Number of subjects; n = Number of subjects in specified group; SD = Standard Deviation

### Safety and Reactogenicity

Two SAEs were documented (Table 2). One subject in Group MM was hospitalized due to "non-cardiac chest pain", and one subject in Group PM was diagnosed with "prostate cancer". Both SAEs were assessed by the site’s principal investigator as unrelated to the trial vaccine.

**Table 2:** Summary of the Safety and Reactogenicity Results (all enrolled subjects, N = 120).

Safety Endpoint all AEs	Group MM (N = 62)		Group PM (N = 58)		Δ (MM–PM) [95% CI]	
	n (%) all AEs	n (%) related AE	n (%) all AEs	n (%) related AE	(Δ%) all AEs	(Δ%) Related AEs
At least one AE	57 (91.9)	34 (54.8) ^3^	55 (94.8)	33 (56.9) ^3^	-2.9 [-13.5, 7.3]	-2.1 [-15.8, 20.1]
SAE	1 (1.6)	0 (0.0)	1 (1.7)	0 (0.0)	-0.1 [-7.9, 7.3]	NA
AESI	5 (8.1)	0 (0.0)	0 (0.0)	0 (0.0)	8.1 [1.1, 17.8]	NA
AE leading to discontinuation of 2^nd^ vaccination.	4 (6.5)	0 (0.0)	0 (0.0)	0 (0.0)	6.5 [-0.2. 15.7]	NA
AE leading to withdrawal from trial	1 (1.6)	0 (0.0)	0 (0.0)	0 (0.0)	1.6 [-4.9, 9.0]	NA
AEs of Grade ≥ 3	11 (17.7)	3 (4.8)	4 (6.9)	1 (1.7)	10.8 [-1.4, 23.7]	3.1 [-5.0, 12.3]
Unsolicited AEs	36 (58.1)	17 (27.4)	31 (53.4)	19 (32.8)	4.7 [-13.6, 22.4]	-5.3 [-22.1, 11.4]
Safety Endpoint Solicited AEs	n (%) all solicited AEs	n (%) Grade ≥3 solicited AEs	n (%) all solicited AEs	n (%) Grade ≥3 solicited AEs	(Δ%) all AEs	(Δ%) Related AEs
Solicited local AE (after first administration ^1^)	53 (85.5)	4 (6.5)	12 (20.7)	0 (0.0)	64.8 [48.4, 77.1]	6.5 [-0.3, 15.7]
Solicited local AE (after second administration ^2^)	45 (72.6)	4 (7.1)	46 (79.3)	2 (3.4)	-6.7 [-22.2, 9.0]	3.0 [-6.3, 12.7]
Solicited general AEs (after first administration ^1^)	27 (43.5)	3 (4.8)	15 (25.9)	0 (0.0)	17.7 [-0.0, 34.3]	4.8 [-1.8, 13.8]
Solicited general AEs (after second administration ^2^)	21 (33.9)	1 (1.8)	25 (43.1)	1 (1.7)	-9.2 [-26.9, 8.7]	-0.1 [-7.9, 7.3]

AE = adverse event, N = Number of subjects in specified group, n = number of subjects with at least one respective AE, SAE = serious adverse event, AESI = AE of special interest.

^1^ = The first administration for Group MM is the first MVA vaccination, for Group PM it is a placebo administration.

^2^ = The second administration for Group MM is the second MVA vaccination, for Group PM it is the first MVA vaccination.

^3^ = Solicited local AEs are excluded.

AESIs were defined as any cardiac symptoms, ECG changes determined to be clinically significant or Troponin I elevated twice above normal limit. Five not clinically significant AESIs (cardiac symptoms or ECG changes) were reported for five subjects in Group MM after the first MVA vaccination (Table 2). Four of these AESIs were assessed by the investigator as unrelated, and one as unlikely related to the trial vaccine. Four of the five subjects did not receive the second vaccination due to the AESI and one subject with supraventricular extra systoles was withdrawn from the trial. No AESIs were documented for Group PM. No case of myopericarditis was observed in either group.

As shown in Table 2, the incidence of unsolicited AEs was comparable for Group MM and Group PM (58.1% and 53.4%, respectively). Most unsolicited AEs were unrelated to the trial vaccine. Unsolicited AE with relationship to the vaccine of at least “possible” were observed in 47.3% of subjects in Group MM and in 50.8% of subjects in Group PM (S3 Table). The incidence after each administration was comparable within each group. The incidence of related unsolicited AEs was not higher after administration of MVA than after administration of placebo. Unsolicited AEs were not more frequent, whether MVA was given once or twice (S4 Table, S5 Table). Furthermore there was no distinct pattern of AEs according to system organ class (S6 Table).

Solicited local AEs are per definition classified as related. The overall incidence of solicited local AEs was similar in both groups after each vaccination with MVA (85.5% and 72.6% in Group MM and 79.3% in Group PM) with no increase from first to second vaccination observed in Group MM (Table 2). After placebo administration in Group PM, an incidence of 20.7% solicited local AEs were reported (which increased to 79.3% after vaccination with MVA). In both groups, "erythema" was the most frequently reported solicited local AE, followed by "pain" and "swelling".

"Myalgia", "fatigue" and "headache" were the most frequent solicited general AEs in both groups after each vaccination. The overall incidence of solicited general AEs after each trial vaccine administration was similar in both groups (43.5% and 33.9% in Group MM and 43.1% in Group PM) with no increase from first to second vaccination observed in Group MM. After placebo administration an incidence of 25.9% was reported for solicited general AEs in Group PM (which increased to 43.1% after the vaccination with MVA). The incidence of grade ≥3 AEs assessed as related to vaccination was very low and comparable between the two groups with three cases in Group MM (4.8%) and one case in Group PM (1.7%).

There were no relevant changes in mean and median hematology and biochemistry values during the trial. There were only minor mean and median changes in vital signs at the various trial visits compared to the visit 1 (prior to first vaccination; baseline) in both groups.

### Immunogenicity

At baseline 98.4% of subjects in Group MM and 94.8% of subjects in Group PM were seropositive in the ELISA and 72.1% and 69.0% were seropositive in the PRNT, respectively (Table 3). The ELISA seroconversion rate two weeks after the first dose of MVA was 83.6% in Group MM (week 2) and 82.8% in Group PM (week 6). Two weeks after the second dose of MVA (Group MM only; week 6)) the seroconversion rate was 83.3% (Δ (MM–PM) = 0.6%; 95% CI = -13.8, 15.0) (Table 3).

**Table 3:** Seropositivity and Seroconversion Rates (IAS, N = 119^1^).

	Group MM	Group PM	Difference
**ELISA**	(N = 61) % [95% CI]^2^	(N = 58) % [95% CI]^2^	Δ% (MM—PM) [95% CI]^3^
Baseline (S+)	98.4 [91.2, 100]	94.8 [85.6, 98.9]	3.5 [-4.6, 13.0]
Week 2 (SC)	83.6 [71.9, 91.8]	1.7% [0.0, 9.2]	81.9 [69.8, 90.7]
Week 6 (SC)	83.3 [71.5, 91.7]	82.8 [70.6, 91.4]	0.6 [-13.8, 15.0]
FU (week 32) (SC)	59.3 [45.7, 71.9]	58.6 [44.9, 71.4]	0.7 [-17.0, 18.3]
Indiv. Peak (SC)	90.2 [79.8, 96.3]	84.5 [72.6, 92.7]	5.7[-6.8, 18.8]
**PRNT**	Group MM (N = 61) % [95% CI]^2^	Group PM (N = 58) % [95% CI]^2^	Difference Δ% (MM—PM) [95% CI]^3^
Baseline (S+)	72.1 [59.2, 82.9]	69.0 [55.5, 80.5]	3.2 [-13.4, 19.8]
Week 2 (SC)	73.8 [60.9, 84.2]	10.3 [3.9, 21.2]	63.4 [47.4, 76.2]
Week 6 (SC)	90.0 [79.5, 96.2]	77.6 [64.7, 87.5]	12.4 [-1.1, 27.0]
FU (week 32) (SC)	55.9 [42.4, 68.8]	41.4 [28.6, 55.1]	9.2 [-4.0, 32.6]
Indiv. Peak (SC)	95.1 [86.7, 99]	77.6 [64.7, 87.5]	17.5 [4.4, 30.9]

ELISA = enzyme-linked immunosorbent assay, IAS = Immunogenicity Analysis Set, GMT = geometric mean titer, N = Number of subjects in specified group, NA = Not Applicable, PRNT = plaque reduction neutralization test, SC = seroconversion, S+ = seropositivity. Administrations at week 0 (Group MM: first MVA vaccination; Group PM: Placebo) and at week 4 (Group MM: second MVA vaccination; Group PM: first MVA vaccination).

^1^ = One subject was excluded from the IAS because baseline results were missing.

^2^ = 95% Clopper Pearson CI of the proportions

^3^ = 95% exact two one-sided CI of the difference in proportions.

The PRNT seroconversion rates two weeks after the first dose of MVA were comparable with 73.8% in Group MM (week 2) and 77.6% in Group PM (week 6). However, the second MVA vaccination in Group MM increased the seroconversion rate to 90.0% (week 6) (Δ (MM–PM) = 12.4%; 95% CI = -1.1, 27.0) (Table 3).

**Fig 2:**
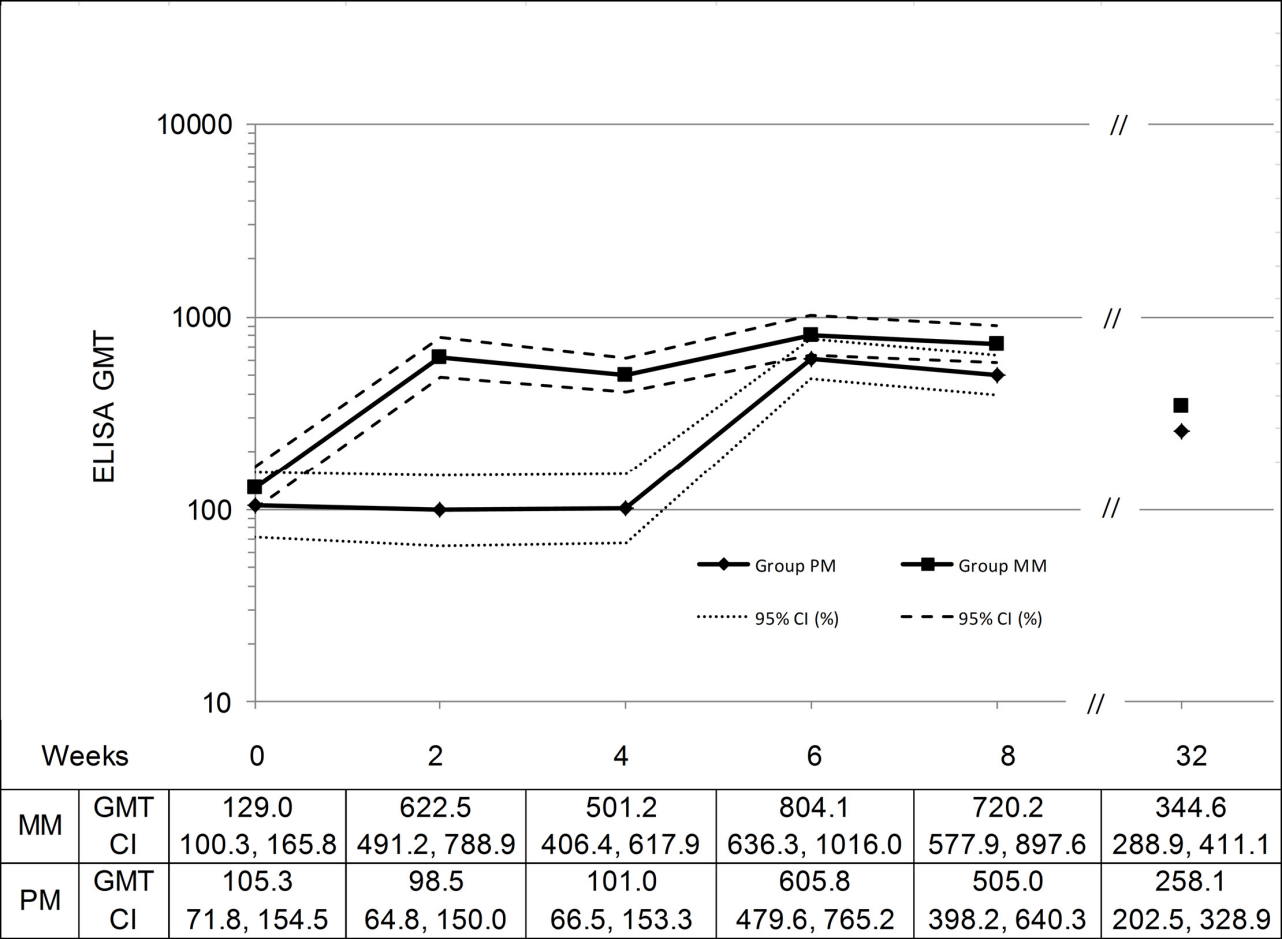
Vaccinia-specific ELISA GMTs by Week (IAS, N = 119). Administrations at week 0 (Group MM: first MVA vaccination; Group PM: Placebo) and at week 4 (Group MM: second MVA vaccination; Group PM: first MVA vaccination). No samples were taken between week 8 and 32, therefore the graph was cut. IAS = Immunogenicity Analysis Set, GMT = geometric mean titer, ELISA = enzyme-linked immunosorbent assay, CI = confidence interval.

In line with the high seroconversion rate, one vaccination with MVA resulted in substantial vaccinia specific antibody titers. ELISA GMT values two weeks after the first vaccination with MVA were 622.5 in Group MM and 605.8 in Group PM (Table 4, Fig 2), representing 4.8 and 5.8-fold compared to the baseline titers respectively. After two doses of MVA, the GMT in Group MM increased to 804.1, higher than the GMT determined in Group PM after one MVA vaccination (Ratio (MM/PM) = 1.33; 95% CI = 0.96, 1.84)

**Table 4:** Geometric Mean Titers and Geometric Fold Ratios (IAS, N = 119^1^).

**ELISA**	Group MM (N = 61)	Group PM (N = 58)	Ratio (MM / PM) [95% CI]
Baseline GMT [CI]	129.0 [100.3, 165.8]	105.3 [71.8, 154.5]	1.22 [0.78, 1.92]
Week 2 GMT [CI] (Fold Ratio) [CI]	622.5 [491.2, 788.9] (4.83) [3.76, 6.19]	98.5 [64.8, 150.0] (0.94) [0.81, 1.08]	6.32 [3.94, 10.11] (5.16) [3.87, 6.87]
Week 6 GMT [CI] (Fold Ratio) [CI]	804.1 [636.3, 1016] (6.21) [4.62, 8.35]	605.8 [479.6, 765.2] (5.75) [4.20, 7.87]	1.33 [0.96, 1.84] (1.08) [0.70, 1.65]
Week 32 GMT [CI] (Fold Ratio) [CI]	344.6 [288.9, 411.1] (2.70) [2.16, 3.38]	258.1 [202.5, 328.9] (2.45) [1.82, 3.29]	1.34 [0.99, 1.79] (1.10) [0.76, 1.59]
Indiv. Peak GMT [CI] (Fold Ratio) [CI]	992.4 [769.2, 1280.3] (7.70) [5.69, 10.40]	645.2 [505.0, 824.3] (6.12) [4.48, 8.38]	1.54 [1.08, 2.18] (1.26) [0.82, 1.93]
**PRNT**	Group MM (N = 61)	Group PM (N = 58)	Ratio (MM / PM) [95% CI]^2^
Baseline GMT [CI]	11.9 [7.4, 19.1]	11.3 [6.5, 19.4]	1.06 [0.52, 2.15]
Week 2 GMT [CI] (Fold Ratio) [CI]	111.4 [72.0, 172.2] (9.36) [6.01, 14.57]	9.2 [5.3, 15.8] (0.82) [0.58, 1.15]	12.12 [6.1, 24.1] (11.48) [6.56, 20.10]
Week 6 GMT [CI] (Fold Ratio) [CI]	210.3 [146.1, 302.7] (17.74) [11.79, 26.69]	126.7 [82.4, 194.8] (11.24) [7.04, 17.94]	1.66 [0.95, 2.90] (1.58) [0.85, 2.92]
Week 32 GMT [CI] (Fold Ratio) [CI]	47.0 [31.1, 71.1] (4.11) [2.81, 5.98]	27.6 [17.0, 44.8] (2.45) [1.59, 3.77]	1.70 [0.91, 3.20] (1.68) [0.95, 2.95]
Indiv. Peak GMT [CI] (Fold Ratio) [CI]	257.6 [178.6, 371.5] (21.65) [14.18, 33.06]	139.6 [89.3, 219.2] (12.41) [7.75, 19.88]	1.84 [1.04, 3.26] (1.74) [0.93, 3.26]

ELISA = enzyme-linked immunosorbent assay, IAS = Immunogenicity Analysis Set, GMT = geometric mean titer, N = Number of subjects in specified group, NA = Not Applicable, PRNT = plaque reduction neutralization test

Administrations at week 0 (Group MM: first MVA vaccination; Group PM: Placebo) and at week 4 (Group MM: second MVA vaccination; Group PM: first MVA vaccination).

^1^ = One subject was excluded from the IAS because baseline results were missing.

^2^ = 95% one sample t-distribution CI.

**Fig 3:**
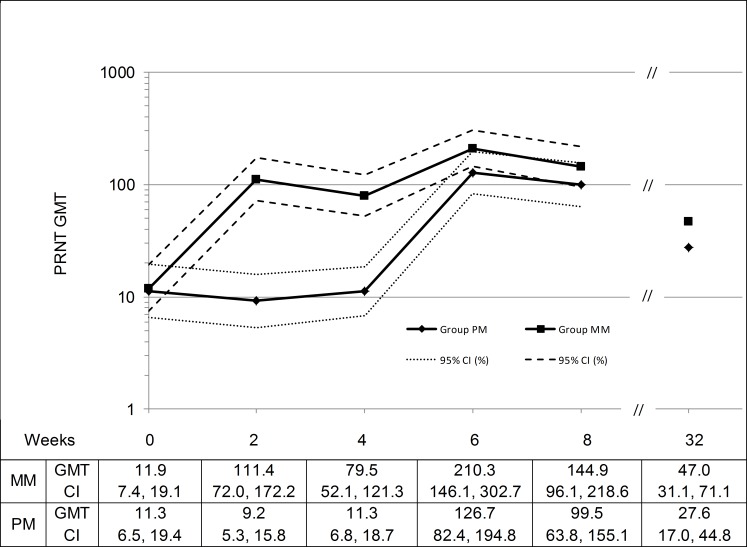
Vaccinia-specific PRNT GMTs by Week (IAS, N = 119). Administrations at week 0 (Group MM: first MVA vaccination; Group PM: Placebo) and at week 4 (Group MM: second MVA vaccination; Group PM: first MVA vaccination). No samples were taken between week 8 and 32, therefore the graph was cut. IAS = Immunogenicity Analysis Set, GMT = geometric mean titer, PRNT = plaque reduction neutralization test, CI = confidence interval.

The PRNT GMTs after the first MVA vaccination were reported as 111.4 for Group MM and 126.7 for Group PM (Table 4, Fig 3) representing 9.4 and 11.2-fold compared to the baseline titers respectively. The second MVA dose in Group MM nearly doubled the GMT (210.3) compared to the GMT determined after one MVA vaccination in Group PM (Ratio (MM/PM) = 1.66; 95% CI = 0.95, 2.90).

ELISA and PRNT GMTs per week are shown in Fig 2 and Fig 3, respectively. Starting with comparable baseline titers for subjects in Group MM and Group PM, the GMTs for both assays increased after each vaccination with MVA peaking at week 2 and 6 for Group MM and at week 6 for Group PM. After 32 weeks, the ELISA and PRNT GMTs determined were 344.6 and 47.0 for Group MM and 258.1 and 27.6 for Group PM, which were all higher than the baseline titers. The titers were lower for the group receiving only one vaccination with MVA compared to the group receiving two doses of MVA.

## Discussion

The observed incidences of unsolicited and solicited AEs reported in this population (56 to 80 years of age) were very similar to that previously reported in MVA trials in younger vaccinia experienced adults (≤55 years old) that were either healthy or HIV infected [8, 9,16]. Clinical studies with traditional smallpox vaccines based on replicating VACV strains have reported fewer AEs in vaccinia-experienced subjects compared to vaccinia-naïve populations, hypothesized to be related to the immunologic memory against VACV [28]. Interestingly given that MVA fails to replicate in human cells and as such cannot cause several of the AE associated with the replicating nature of traditional smallpox vaccines (e.g. progressive vaccinia, generalized vaccinia, inadvertent inoculation), the safety profile of MVA has been shown to be the same for vaccinia experienced and vaccinia naïve populations whether for healthy [16] or HIV infected [8, 9] adults.

A high number of suspected and probable myopericarditis cases were reported in the pivotal clinical program of ACAM2000, a replicating VACV [29]. The Phase III ACAM2000 trials included active post-vaccination cardiac monitoring, leading to the detection of 8 myopericarditis cases in 1,162 primary vaccinees after vaccination with either ACAM2000 or Dryvax (incidence of 1:145). Most cases were symptomatic (i.e. subjects presented with chest pain, reduced tolerance to exercise, dyspnea or palpitations) and diagnosis was confirmed by coexisting ECG changes or echocardiogram, findings indicative of myocardial inflammation. During the campaign to vaccinate first line responders in the USA in 2003, the majority (76%) of the 40,000 people vaccinated were vaccinia-experienced and of the 203 cardiac events reported, 183 were in people previously vaccinated [30]. Therefore, serious cardiac events may be associated with vaccination in vaccinia-experienced populations receiving traditional smallpox vaccines. Despite the fact that subjects 56 to 80 years of age in general are at higher risk of experiencing cardiac events [31], no such cases were reported in the current study. Despite close cardiac monitoring, no confirmed cases of myo-pericarditis have been observed in any MVA-BN trials. This supports a favorable cardiac safety profile of MVA for the population of 56 to 80 years of age and confirms results reported in a large clinical trial (N = 745) designed to investigate cardiac safety in 18–55 year old healthy vaccinia-naïve and vaccinia experienced subjects [16].

Several studies have demonstrated that humoral immunity against smallpox is long-lived and can persist for more than 30 years [28, 32–35]. Consistent with this finding, moderate baseline antibody titers were detected in the 56–80 years old subjects enrolled in this study, although primary vaccination of the subjects was likely performed more than 40 years ago. Routine smallpox vaccination in the USA was stopped 1972. The ability of MVA to rapidly boost the antibody responses in all vaccinia-experienced subjects supports the observation that traditional smallpox vaccines induce a long-lived B-cell memory [33]. Historically, booster vaccinations with traditional smallpox vaccines provided a robust protection [36], although the vaccine take, which is an accepted marker of efficacy against smallpox, was often attenuated in vaccinia-experienced subjects. Recently the ability to boost the antibody response in vaccinia-experienced subjects has been suggested to be more indicative of protection than the take rate [29]. Therefore, the anamnestic response recorded following a single booster vaccination with MVA suggests that this vaccine will be effective in 56–80 year old vaccinia-experienced subjects. Even though antibody titers had declined during the 6 month follow-up period of the clinical trial, ELISA and PRNT titers were still considerably above the baseline titers. Since GMTs are currently not an accepted correlate of protection and no protective titer is defined, it is unknown if this antibody level is high enough to protect against smallpox. Clinical proof of efficacy is not possible due to the fact that smallpox is eradicated. A randomized Phase 3 non-inferiority trial is currently ongoing to directly compare indicators of efficacy of MVA to the replicating smallpox vaccine ACAM2000^®^.

Overall this trial found one or two doses of MVA to be safe and immunogenic in a vaccinia-experienced, population 56 to 80 years of age. These results also suggest that a single MVA vaccination boosts the memory response induced by a prior vaccination with a traditional smallpox vaccine. As such this further suggests that this boosted memory response after MVA vaccination should provide efficacy against VARV in humans.

## Supporting Information

S1 Fig Vaccinia-specific ELISA GMTs by Week (PPS, N = 102). Administrations at Week 0 (Group MM: first MVA vaccination; Group PM: Placebo) and at Week 4 (Group MM: second MVA vaccination; Group PM: first MVA vaccination). No samples were taken between week 8 and 32, therefore the graph was cut. PPS = Per Protocol Set, GMT = geometric mean titer, ELISA = enzyme-linked immunosorbent assay, CI = confidence interval, MM = MVA/MVA, PM = Placebo/MVA.
(TIF)

S2 Fig Vaccinia-specific PRNT GMTs by Week (PPS, N = 102). Administrations at Week 0 (Group MM: first MVA vaccination; Group PM: Placebo) and at Week 4 (Group MM: second MVA vaccination; Group PM: first MVA vaccination). No samples were taken between week 8 and 32, therefore the graph was cut. PPS = Per Protocol Analysis Set, GMT = geometric mean titer, PRNT = plaque reduction neutralization test, CI = confidence interval, MM = MVA/MVA, PM = Placebo/MVA.
(TIF)

S1 File CONSORT Checklist. (DOCX)

S2 File Patents. (DOCX)

S3 File Initial Protocol for IRBs. (PDF)

S4 File Poster. (PDF)

S1 Table Demographic Data PPS. (DOCX)

S2 Table Immunogenicity Results PPS. (DOCX)

S3 Table Grade and Relationship of Unsolicited AEs. (DOCX)

S4 Table Unsolicited related AEs after first vaccination. (DOCX)

S5 Table Unsolicited related AEs after second vaccination. (DOCX)

S6 Table Unsolicited AEs ≥ 3%. (DOCX)

S7 Table Baseline Seropositivity Rates. (DOCX)

S8 Table Overview of Immunogenicity Results. (DOCX)

## References

[1] Breman JG, Arita I. The confirmation and maintenance of smallpox eradication. N Engl J Med. 1980, 303(22): 1263–73. 6252467 10.1056/NEJM198011273032204

[2] Mayr A. Smallpox vaccination and bioterrorism with pox viruses, Comp Immun Microbiol Infect Dis 2003, 23, 423–430 10.1016/S0147-9571(03)00025-0 12818626

[3] Artenstein AW, Grabenstein JD. Smallpox vaccines for biodefense: need and feasibility. Expert Rev Vaccines 2008, 7(8): 1225–37. 10.1586/14760584.7.8.1225 18844596 PMC9709930

[4] Lane JM, Ruben FL, Neff JM, Millar JD. Complications of smallpox vaccination, 1968. N Engl J Med. 1969; 281: 1201–1208. 4186802 10.1056/NEJM196911272812201

[5] Lane JM, Ruben FL, Abrutyn E, Millar JD. Deaths attributable to smallpox vaccination, 1959 to 1966, and 1968. JAMA 1970 4 20; 212(3): 441–4. 4392370

[6] Lane JM, Ruben FL, Neff JM, Millar JD. Complications of smallpox vaccination, 1968: results of ten statewide surveys. J Infect Dis. 1970; 122: 303–309. 4396189 10.1093/infdis/122.4.303

[7] Suter M, Meisinger-Henschel C, Tzatzaris M, Hülsemann V, Lukassen S, Wulff N, et al Modified vaccinia Ankara strains with identical coding sequences actually represent complex mixtures of viruses that determine the biological properties of each strain. Vaccine 2009, 27(52): 7442–7450. 10.1016/j.vaccine.2009.05.095 19539582

[8] Greenberg RN, Overton ET, Haas DW, Frank I, Goldman M, von Krempelhuber A et al Safety, Immunogenicity, and Surrogate Markers of Clinical Efficacy for Modified Vaccinia Ankara as a Smallpox Vaccine in HIV-Infected Subjects. J Infect Dis. 2013; 207: 749–758. 10.1093/infdis/jis753 23225902 PMC3611764

[9] Overton E, Stapleton J, Frank I, Hassler S, Goepfert P, Barker D et al Safety and Immunogenicity of Modified Vaccinia Ankara-Bavarian Nordic Smallpox Vaccine in Vaccinia-Naive and Experienced Human Immunodeficiency Virus-Infected Individuals: An open-Label, Controlled Clinical Phase II Trial. Open Forum Infect Dis. 2015 1–10. 10.1093/ofid/ofv040 PMC4567089 26380340

[10] von Sonnenburg F, Perona P, Darsow U, Ring J, von Krempelhuber A, Vollmar J et al Safety and immunogenicity of modified vaccinia ankara as a smallpox vaccine in people with atopic dermatitis. Vaccine 2014, 32: 5696–5702 10.1016/j.vaccine.2014.08.022 25149431

[11] Walsh SR, Wilck MB, Dominguez DJ et al Safety and immunogenicity of modified vaccinia Ankara in hematopoietic stem cell transplant recipients: a randomized, controlled trial. JID 2013, 207, 1888–1897 10.1093/infdis/jit105 23482644 PMC3654753

[12] Frey SE, Newman FK, Kennedy JS et al Clinical and immunologic responses to multiple doses of IMVAMUNE (Modified Vaccinia Ankara) followed by Dryvax challenge. Vaccine 2007, 25: 8562–8573. 18036708 10.1016/j.vaccine.2007.10.017 PMC2713577

[13] Frey S, Winokur P, Salata R, El-Kamary S, Turley C, Walter E Jr, et al Safety and immunogenicity of IMVAMUNE^®^ smallpox vaccine using different strategies for a post event scenario. Vaccine 2013; 31(29): 3025–3033. 10.1016/j.vaccine.2013.04.050 23664987 PMC3755481

[14] Frey S, Winokur P, Hill H, Goll J, Chaplin P and Belshe R. Phase II randomized, double-blinded comparison of a single high dose (5 x 10^8^ TCID_50_) of modified vaccinia Ankara compared to a standard dose (1 x 10^8^ TCID_50_) in healthy vaccinia-naïve individuals. Vaccine 2014; 32(23): 2732–2739. 10.1016/j.vaccine.2014.02.043 24607004 PMC4106233

[15] Frey SE, Wald A, Edupuganti S, Jackson LA, Stapleton JT, El Sahly H et al Comparison of lyophilized versus liquid modified vaccinia Ankara (MVA) formulations and subcutaneous versus intradermal routes od administration in healthy vaccinia-naïve subjects. Vaccine 2015 10.1016/j.vaccine.2015.06.075 PMC9533873 26143613

[16] Zitzmann-Roth E, Von Sonnenburg F, De La Motte S, Arndtz-Wiedemann N, Von Kremplhuber A, Uebler N, et al Cardiac Safety of Modified Vaccinia Ankara for Vaccination against Smallpox in a Young Healthy Study Population. PLOS One 2015, 10(4): e0122653 10.1371/journal.pone.0122653 25879867 PMC4399887

[17] Greenberg RN; Hurley Y; Dinh VD, Mraz S; Gomez Vera J; von Bredow D et al A multicenter, open-label, controlled phase II study to evaluate safety and immunogenicity of MVA smallpox vaccine (IMVAMUNE^®^) in 18–40 year old subjects with diagnosed atopic dermatitis. PLOS One 2015, 10(10): e0138348 10.1371/journal.pone.0138348 26439129 PMC4595076

[18] Vollmar J, Arndtz N, Eckl K, Thomsen T, Petzold B, Mateo L et al Safety and immunogenicity of IMVAMUNE, a promising candidate as a third generation smallpox vaccine. Vaccine 2006, 24(12): 2065–2070. 16337719 10.1016/j.vaccine.2005.11.022

[19] Von Krempelhuber A, Vollmar J, Pokorny R, Rapp P, Wulff N, Petzold B, et al A randomized, double-blind, dose-finding Phase II study to evaluate immunogenicity and safety of the third generation smallpox vaccine candidate IMVAMUNE. Vaccine 2010, 28(5): 1209–1216. 19944151 10.1016/j.vaccine.2009.11.030 PMC2814951

[20] Samuelsson C, Hausmann J, Lauterbach H, Schmidt M, Akira S, Wagner H, et al Survival of lethal poxvirus infection in mice depends on TLR9, and therapeutic vaccination provides protection. J Clin Invest 2008, 118: 1776–1784. 10.1172/JCI33940 18398511 PMC2289795

[21] Garza NL, Hatkin JM, Livingston V, Nichols DK, Chaplin P, Volkmann A et al Evaluation of the efficacy of modified vaccinia Ankara (MVA)/IMVAMUNE against aerosolized rabbitpox virus in a rabbit model Vaccine 2009, 27, 5496–5504. 10.1016/j.vaccine.2009.06.105 19632316 PMC2737728

[22] Stittelaar KJ, van Amerogen G, Kondova I, Kuiken T, van Lavieren RF, Pistoor FH et al Modified vaccinia virus Ankara protects macaques against respiratory challenge with monkeypox virus. J Virol. 2005; 79: 7845–7851. 15919938 10.1128/JVI.79.12.7845-7851.2005 PMC1143678

[23] Gruver AL, Hudson LL, Sempowski GD. Immunosenescence of ageing, J Pathol 2007; 2001: 144–156 10.1002/path.2104 PMC1931833 17200946

[24] Paavonen J, Jenkins D, Bosch FX, Naud P, Salmerón J, Wheeler CM et al Efficacy of a prophylactic adjuvanted bivalent L1 virus-like-particle vaccine against infection with human papillomavirus types 16 and 18 in young women: an interim analysis of a phase III double-blind, randomised controlled trial. Lancet 2007, 30, 2161–2170. 10.1016/S0140-6736(07)60946-5 17602732

[25] Pfister G, Savino W. Can the Immune System Still Be Efficient in the Elderly? An Immunological and Immunoendocrine Therapeutic Perspective. Neuroimmunomodulation 2008, 15, 351–364. 10.1159/000156477 19047811

[26] Mayr A, Hochstein-Mintzel V, Stickl H. Passage history, properties, and use of attenuated vaccinia virus strain MVA. Infection. 1975;3:6–14.

[27] Mayr A, and Danner K. Vaccination against pox diseases under immunosuppressive conditions, Dev. Biol. Stand.1978, 41: 225–234. 223909

[28] Greenberg RN, Kennedy JS, Clanton DJ, Plummer EA, Hague L, Ennis FA et al Safety and immunogenicity of new cell-cultured smallpox vaccine compared with calf-lymph derived vaccine: a blind, single-centre, randomized controlled trial. Lancet 2005, 365, 398–409. 15680454 10.1016/S0140-6736(05)17827-1

[29] ACAM2000 Vaccines and Related Biological Products Advisory Committee (VRBPAC) Briefing Document, April 2007. Available: http://www.fda.gov/ohrms/dockets/ac/07/briefing/2007-4292B2-00-index.htm

[30] Chasey CG, Iskander JK, Roper MH, Mast EE, Wen XJ, Török TJ et al Adverse events associated with Smallpox Vaccination in the United States, January-October 2003. JAMA 2005, 294 (21), 2734–2743. 16333009 10.1001/jama.294.21.2734

[31] McMurray JJ and Stewart S Epidemionolgy, aetiology, and prognosis of heart failure Heart 2000, 83, 596–602. 10768918 10.1136/heart.83.5.596 PMC1760825

[32] Taub DD, Ershler WB, Janowski M, Artz A, Key ML, McKelvey et al Immunity from Smallpox Vaccine persists for decades: A longitudinal study. Am J Med 2008, 121, 1058–1064. 10.1016/j.amjmed.2008.08.019 19028201 PMC2610468

[33] Crotty S, Felgner P, Davies H, Glidewell J, Villarreal L, Ahmed R. Cutting edge: long-term B cell memory in humans after smallpox vaccination. J Immunol 2003; 171: 4969–4973 14607890 10.4049/jimmunol.171.10.4969

[34] El-Ad B, Roth Y, Winder A, Tochner Z, Lublin-Tennenbaum T, Katz E et al The persistence of neutralizing antibodies after revaccination against smallpox. J Infect Dis 1990; 161: 446–448. 2155973 10.1093/infdis/161.3.446

[35] Hammarlund E, Lewis MW, Hansen SG, Strelow LI, Nelson JA, Sexton GJ et al Duration of antiviral immunity after smallpox vaccination. Nat Med 2003; 9: 1131–1137. 12925846 10.1038/nm917

[36] Fenner F, Henderson DA, Arita I, Jezek Z and Ladnyi ID. Smallpox and its Eradication. World Health Organization 1988, Geneva, History of International Public Health, No. 6.

