# Supplementary figures and images for: A Randomized, Double-Blind, Placebo-Controlled Phase II Trial Investigating the Safety and Immunogenicity of Modified Vaccinia Ankara Smallpox Vaccine (MVA-BN®) in 56-80-Year-Old Subjects
Source: PLoS One. 2016 Jun 21;11(6):e0157335. doi: 10.1371/journal.pone.0157335 (PMC4915701; doi:10.1371/journal.pone.0157335)

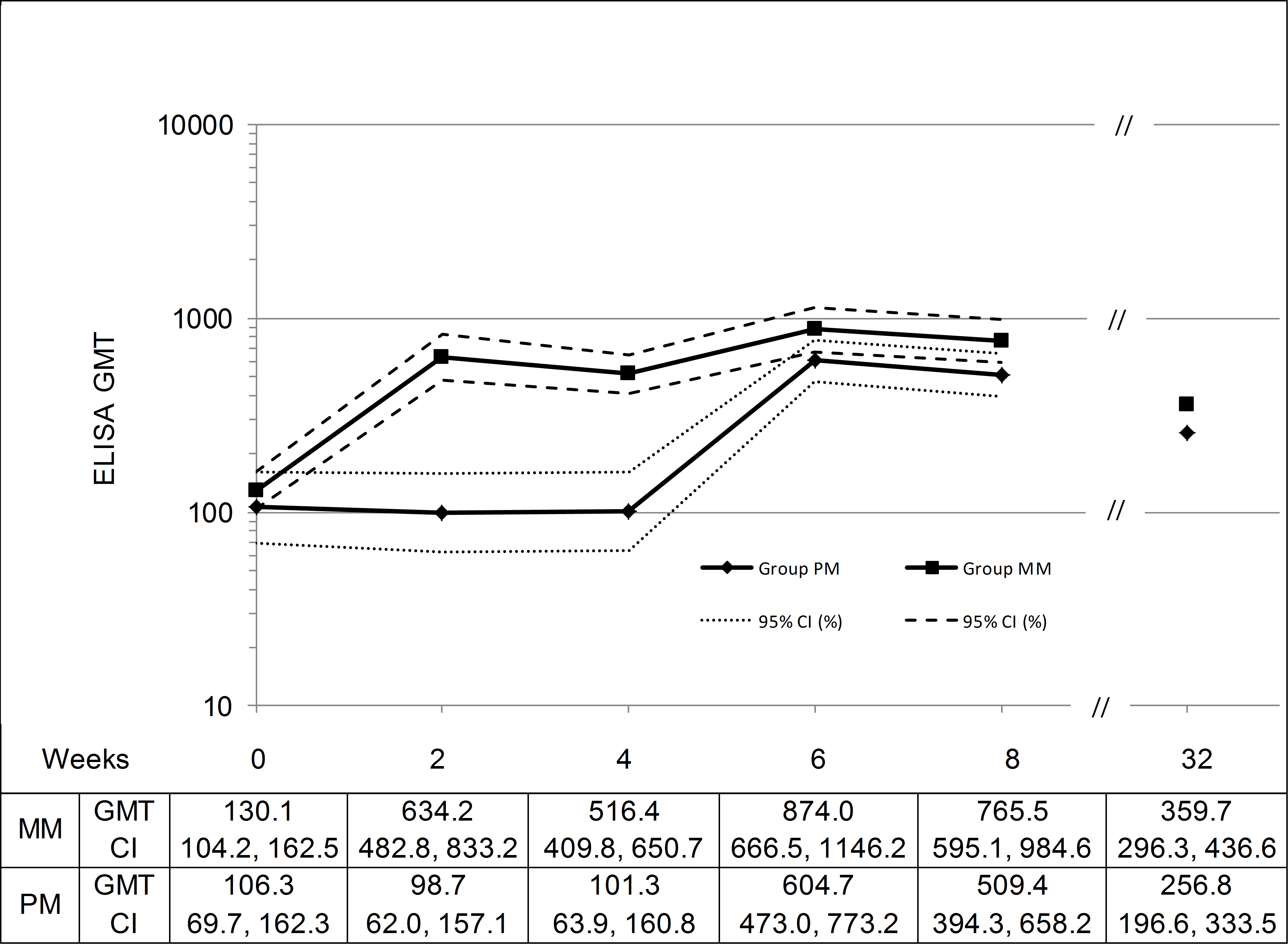

Supplement: S1 Fig — Administrations at Week 0 (Group MM: first MVA vaccination; Group PM: Placebo) and at Week 4 (Group MM: second MVA vaccination; Group PM: first MVA vaccination). No samples were taken between week 8 and 32, therefore the graph was cut. PPS = Per Protocol Set, GMT = geometric mean titer, ELISA = enzyme-linked immunosorbent assay, CI = confidence interval, MM = MVA/MVA, PM = Placebo/MVA. (TIF) [file pone.0157335.s001.tif]

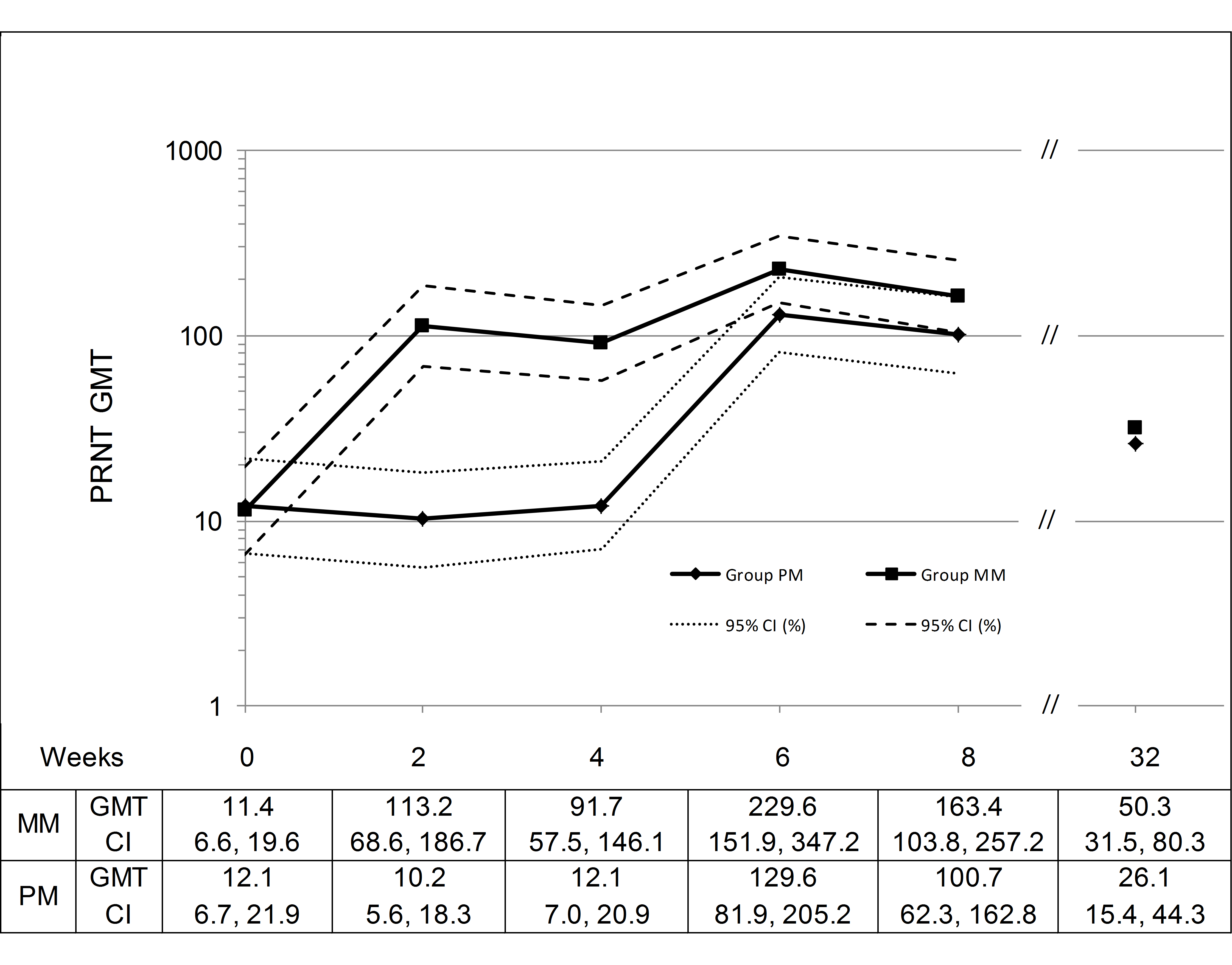

Supplement: S2 Fig — Administrations at Week 0 (Group MM: first MVA vaccination; Group PM: Placebo) and at Week 4 (Group MM: second MVA vaccination; Group PM: first MVA vaccination). No samples were taken between week 8 and 32, therefore the graph was cut. PPS = Per Protocol Analysis Set, GMT = geometric mean titer, PRNT = plaque reduction neutralization test, CI = confidence interval, MM = MVA/MVA, PM = Placebo/MVA. (TIF) [file pone.0157335.s002.tif]
