# Supplementary material for: A Randomized, Double-Blind, Placebo-Controlled Phase II Trial Investigating the Safety and Immunogenicity of Modified Vaccinia Ankara Smallpox Vaccine (MVA-BN®) in 56-80-Year-Old Subjects
Source: PLoS One. 2016 Jun 21;11(6):e0157335. doi: 10.1371/journal.pone.0157335 (PMC4915701; doi:10.1371/journal.pone.0157335)
Supplement: S3 File — (PDF) [file pone.0157335.s005.pdf]

POX-MVA-024

DMID 08-0019

A randomized, double-blind, placebo-controlled Phase II study to evaluate safety and immunogenicity of one and two doses of IMVAMUNE® smallpox vaccine in 56-80 year old vaccinia-experienced subjects

1. General Information

1.1 Site Signature Page

Herewith I agree that I have read and fully understood this protocol:

A randomized, double-blind, placebo-controlled Phase II study to evaluate safety and immunogenicity of one and two doses of IMVAMUNE® smallpox vaccine in 56-80 year old vaccinia-experienced subjects, Version 1.0 dated 18-MAR-2009, DMID 08-0019

This trial protocol describes all the information necessary to conduct the study. I agree that I will conduct the study according to the instructions given within this protocol. Furthermore, I agree that I will conduct this study according to International Conference of Harmonization of Technical Requirements for Registration of Pharmaceuticals for Human Use (ICH) Good Clinical Practice (GCP), the current version of the Declaration of Helsinki, US Code of Federal Regulations (CFR) applicable to clinical studies and local legal and regulatory requirements.

I agree that all information given in this protocol will be handled with strict confidentiality.

Additionally, I will permit trial related monitoring, audits, Institutional Review Board (IRB) / Independent Ethics Committee (IEC) review and regulatory inspections, providing direct access to source data/documents as required.

Date

[name]
Principal Investigator (PI), [site]

[site address]

1.2 Signature Page

By signing the protocol:

A randomized, double-blind, placebo-controlled Phase II study to evaluate safety and immunogenicity of one and two doses of IMVAMUNE® smallpox vaccine in 56-80 year old vaccinia-experienced subjects, Version 1.0, dated 18-MAR-2009, DMID 08-0019

The undersigned parties agree that the protocol was written according to international ethical and scientific quality standards (ICH GCP), in compliance with the current version of the Declaration of Helsinki, the CFR applicable to clinical studies and local legal and regulatory requirements.

19-MAR-2009<br>Date	Eve Wagner on behalf of Siegfried Rösch<br>Siegfried Roesch, MSc<br>Coordinating Protocol Author<br>Bavarian Nordic GmbH
19-Mar-2009<br>Date	Alfred von Krempelhuber, PhD<br>Manager Clinical Development<br>Bavarian Nordic GmbH
19-Mar-2009<br>Date	Garth Virgin<br>Garth Virgin, MD<br>Medical Monitor<br>Bavarian Nordic GmbH
18-Mar-2009<br>Date	Thomas Meyer<br>Thomas Meyer, PhD<br>Manager Clinical Analysis<br>Bavarian Nordic GmbH
18-MAR-2009<br>Date	Philip Young<br>Philip Young, PhD<br>Biostatistician and Data Manager<br>Bavarian Nordic GmbH
19*<br>~~18~~-Mar-2009<br>Date	Robert Semich<br>Robert Semich, PhD<br>GCP Specialist, Quality Assurance Development<br>Bavarian Nordic GmbH
18-MAR-2009<br>Date	Paul Chaplin, PhD<br>Chief Scientific Officer<br>Bavarian Nordic GmbH

* writing error, correct date 19-Mar-2009

Signature Page (cont.)

By signing the protocol:

A randomized, double-blind, placebo-controlled Phase II study to evaluate safety and immunogenicity of one and two doses of IMVAMUNE® smallpox vaccine in 56-80 year old vaccinia-experienced subjects, Version 1.0, dated 18-MAR-2009, DMID 08-0019

The undersigned parties agree that the protocol was written according to international ethical and scientific quality standards (ICH GCP), in compliance with the current version of the Declaration of Helsinki, the CFR applicable to clinical studies and local legal and regulatory requirements.

March 18, 2009
Date

Prof. Richard Greenberg, MD
Coordinating Investigator
University of Kentucky
School of Medicine Department of Medicine

1.3 Responsibilities

Study Number	POX-MVA-024<br>DMID 08-0019
Study Title	A randomized, double-blind, placebo-controlled Phase II study to evaluate safety and immunogenicity of one and two doses of IMVAMUNE® smallpox vaccine in 56-80 year old vaccinia-experienced subjects.
Principal Investigator and Study Site	Professor Richard Greenberg<br>VA staff physician, Lexington VA Medical Center Professor of Medicine, The Belinda Mason Carden and Paul Mason Professor of HIV/AIDS Research and Education<br>University of Kentucky, School of Medicine, Department of Medicine<br>Room MN-672<br>800 Rose Street,<br>Lexington, KY 40536-0084<br>USA
Phone	+1 859 323 6327
Fax	+1 859 323 1631
E-mail	rngree01@email.uky.edu
Sponsor and Product Supply IMVAMUNE®	Bavarian Nordic A/S<br>Hejreskovvej 10A<br>DK-3490 Kvistgård<br>Denmark
Phone	+45 33 268 383
Fax	+45 33 268 380
Product Manufacturer (Placebo)	Bavarian Nordic GmbH<br>Robert-Roessle-Strasse 10<br>13125 Berlin<br>Germany
Phone	+49 30 9406 3900
Fax	+49 30 9406 3999

Project Leader	Eva Wagner, PhD<br>Bavarian Nordic GmbH<br>Fraunhoferstrasse 13<br>82152 Martinsried<br>Germany
Phone	+49 89 8565 1342
Fax	+49 89 8565 1333
E-mail	eva.wagner @bavarian-nordic.com

Project Manager	Alfred von Krempelhuber, PhD<br>Bavarian Nordic GmbH<br>Fraunhoferstrasse 13<br>82152 Martinsried<br>Germany
Phone	+49 89 8565 1369
Fax	+49 89 8565 1333
E-mail	alfred.krempelhuber@bavarian-nordic.com

Medical Monitor	Garth Virgin, MD<br>Director Drug Safety<br>Bavarian Nordic GmbH<br>Fraunhoferstrasse 13<br>82152 Martinsried<br>Germany
Phone	+49 89 8565 1319
Fax	+49 89 8565 1419
E-mail	garth.virgin@bavarian-nordic.com

Study Statistician	Philip Young PhD<br>Bavarian Nordic GmbH<br>Fraunhoferstrasse 13<br>82152 Martinsried<br>Germany
Phone	+49 89 8565 1462
Fax	+49 89 8565 1333
E-mail	philip.young@bavarian-nordic.com

CRO/Project Leader	Cyndy Hughes<br>Kendle International Inc.<br>36 Tumbleweed Rd.<br>Sandia Park, NM 87047
Phone	+1 505 286 8683
Fax	+1 866 570 4552
E-mail	hughes.cynthial@kendle.com

Laboratory (Safety)	Nicole Lester<br>Quintiles Laboratories, Ltd.<br>1600 Terrell Mill Road, Ste. 100<br>Marietta, GA 30067
Phone	+1 770-373-3610
Fax	+1 770-373-3741
E-mail	nicole.lester@quintiles.com

Laboratory (Immunology)	Thomas Meyer, PhD<br>Bavarian Nordic GmbH<br>Fraunhoferstrasse 13<br>82152 Martinsried<br>Germany
Phone	+49 89 8565 1405
Fax	+49 89 8565 1333
E-mail	thomas.meyer@bavarian-nordic.com

Centralized ECG Assessment	Lisa McKnight<br>Cardiocore<br>6901 Rockledge Drive<br>Suite #700<br>Bethesda, MD 20817
Phone	+1 301-214-7617
Fax	+1 301-214-7620
E-mail	lisa.mcknight@cardiocore.com

Electronic Data Capture	Valery Verpale<br>Unithink<br>Waterfront Research Park, Galileilaan 18<br>2845 Niel<br>Belgium
Phone	+32 32980057
Fax	+32 32980047
E-mail	valerie.verpale@unithink .com

1.4 Table of Contents

1. General Information 2
1.1 Site Signature Page 2
1.2 Signature Page 3
Signature Page (cont.) 4
1.3 Responsibilities 5
1.4 Table of Contents 8
1.5 List of Abbreviations 11
1.6 Protocol Synopsis 13
1.7 Flow Chart 20
2. Background Information 21
2.1 First Generation Smallpox Vaccines 21
2.2 Second Generation Smallpox Vaccines 22
2.3 Origin and Characteristics of IMVAMUNE® 23
2.4 Summary of Non-clinical Data with IMVAMUNE® 23
2.5 Clinical Profile of IMVAMUNE® 24
2.5.1 Safety Overview of IMVAMUNE® 26
2.5.1.1 SAEs 26
2.5.1.2 Adverse Events / Reactions 27
2.5.2 Immunogenicity Overview of IMVAMUNE® 30
2.6 Rationale 34
2.7 Study Population 35
2.8 Benefit/Risk Assessment 35
2.8.1 Risks for Study Participants 35
2.8.2 Benefits for Study Participants 36
3. Objectives 36
4. Study Design 36
4.1 Experimental Design 36
4.2 Description of Study Procedures 37
4.2.1 Screening Phase 37
4.2.2 Active Study Phase 38
4.2.3 Unscheduled Visits 41
4.2.4 Follow-up Phase 41
4.3 Study Duration 41
4.4 Study Halting Rule 41
4.5 DSMB 42
4.6 Interim Safety Review 42
5. Selection of Subjects 43
5.1 Recruitment Procedure 43
5.2 Inclusion Criteria 43
5.3 Exclusion Criteria 44
6. Withdrawal of Subjects 45
6.1 Individual Withdrawal Criteria during the Study 45
6.2 Contraindications and Precautions for further Study Vaccinations 46
6.3 Subject Withdrawal Procedure 46
7. Study Treatment 47

7.1 Investigational Product ... 47
7.2 Packaging and Labeling ... 47
7.3 Storage and Handling ... 48
7.4 Randomization ... 48
7.5 Preparation, Administration and Dosage ... 49
7.6 Accountability and Disposal ... 50
7.7 Concomitant Medication ... 50
8. Clinical and Laboratory Assessments ... 50
8.1 General ... 50
8.1.1 Medical History ... 50
8.1.2 Complete Physical Examination ... 51
8.1.3 Targeted Physical Examination ... 51
8.1.4 ECG and Cardiac Event Assessment ... 51
8.2 Assessment of Safety and Reactogenicity ... 54
8.2.1 Assessment of General Safety Parameters ... 54
8.2.1.1 Vital signs ... 54
8.2.1.2 Laboratory Measurements ... 54
8.2.1.3 Safety Laboratory ... 54
8.2.2 Assessment of AEs ... 55
8.2.2.1 Assessment of Solicited Local AEs ... 55
8.2.2.2 Assessment of Solicited Systemic AEs ... 56
8.2.2.3 Assessment of Unsolicited AEs ... 57
8.2.2.4 Assessment of Severity for AEs ... 57
8.2.2.5 Assessment of Laboratory Measured Toxicities ... 57
8.2.2.6 Causality Assessment ... 58
8.3 Assessment of Immunogenicity ... 59
8.3.1 Antibody Response ... 59
8.3.1.1 ELISA ... 59
8.3.1.2 PRNT ... 60
9. Safety Assessment and Reporting ... 60
9.1 Definition of AE ... 60
9.1.1 Definition of Solicited AE ... 60
9.1.2 Definition of Unsolicited AE ... 61
9.1.3 Definition of "AE of Special Interest" (SIAE) ... 61
9.2 Definition of SAE ... 61
9.3 Reporting of SAEs ... 62
9.4 Pregnancy ... 64
10. Statistical Considerations ... 65
10.1 Statistical Analysis Plan ... 65
10.2 Sample Size Calculations ... 65
10.3 Endpoints ... 66
10.3.1 Safety and Reactogenicity ... 66
10.3.2 Immunogenicity ... 67
10.3.2.1 ELISA ... 67
10.3.2.2 PRNT ... 67
10.4 Study Cohorts/Data Sets to be evaluated ... 68
10.5 Biometrical Evaluation ... 69

10.5.1 Analysis of Demographics and Baseline Characteristics ... 69
10.5.2 Analysis of Immunogenicity ... 69
10.5.3 Analysis of Safety and Reactogenicity ... 70
10.5.3.1 Solicited Local AEs ... 70
10.5.3.2 Solicited General AEs ... 71
10.5.3.3 Unsolicited AEs: ... 71
10.5.3.4 SAEs ... 71
10.5.3.5 Safety Laboratory ... 71
10.5.3.6 Vital Signs and Physical Examinations ... 72
10.5.3.7 Concomitant Medication ... 72
10.5.4 Data Handling ... 72
11. Ethical Aspects ... 72
11.1 Ethical and Legal Regulations ... 72
11.2 Approval of an IEC/IRB and Institutional Biosafety Committee (IBC) ... 73
11.3 Confidentiality and Data Protection ... 73
12. Informed Consent ... 73
13. CRFs and Retention of Records ... 74
13.1 Electronic CRFs ... 74
13.2 Retention of records ... 75
14. Monitoring of the Study ... 75
15. Responsibilities of the Investigator ... 76
16. References ... 78
17. Appendices ... 80
17.1 Appendix I: Toxicity Scale ... 80
17.2 Appendix II: Case Definitions Acute Myocarditis / Pericarditis ... 82
17.3 Appendix III: Test of understanding ... 83

1.5 List of Abbreviations

AD	Atopic Dermatitis
ADR	Adverse Drug Reaction
AE	Adverse Event
AIDS	Acquired Immunodeficiency Syndrome
ALT	Alanine Aminotransferase
AST	Aspartate Aminotransferase
ATC	Anatomical-Therapeutic-Chemical Classification
BN	Bavarian Nordic A/S
CEF	Chicken Embryo Fibroblasts
CFR	US Code of Federal Regulations
CDISC	Clinical Data Interchange Standards Consortium
CRF(s)	Case Report Form(s)
CRO	Contract Research Organization
CVA	Chorioallantois Vaccinia Virus Ankara
DMID	Division of Microbiology and Infectious Diseases
DSMB	Data Safety Monitoring Board
ECG	Electrocardiogram
ECTV	Ectromelia Virus
EDC	Electronic Data Capture
ELISA	Enzyme-linked Immunosorbent Assay
FAS	Full Analysis Set
FDA	US Food and Drug Administration
FU	Follow-up
GCP	Good Clinical Practice
GMT	Geometric Mean Titer
HCG	Human Choriogonadotropin
HIPAA	Health Insurance Portability and Accountability Act
HIV	Human Immunodeficiency Virus
ICF	Informed Consent Form
ICH	International Conference on Harmonization of Technical Requirements for Registration of Pharmaceuticals for Human Use
IEC	Independent Ethics Committee
IgG	Immunoglobuline G
IFN	Interferon
i.m.	Intramuscular
i.n.	Intranasal
IND	Investigational New Drug
IRB	Institutional Review Board
IBC	Institutional Biosafety Committee
LLN	Lower Limit of Normal
LOCF	Last Observation Carried Forward
MLD_{50}	Murine Lethal Dose 50
MVA	Modified Vaccinia Ankara
MVA-BN®	Modified Vaccinia Ankara – Bavarian Nordic

n/N	Number
NIAID	National Institute of Allergy and Infectious Diseases
NIH	National Institute of Health
NYCBH	New York City Board of Health
ODM	Operational Data Modeling
PI	Principal Investigator
PPS	Per Protocol Set
PRNT	Plaque Reduction Neutralization Test
PVC	Premature Ventricular Contractions
SAE	Serious Adverse Event
SAP	Statistical Analysis Plan
s.c.	Subcutaneous
SCR	Screening
SIAE	Special Interest Adverse Event
ST	ST Segment in Electrocardiogram
$TCID_{50}$	Tissue Culture Infectious Dose 50%
ULN	Upper Limit of Normal
US	United States of America
V	Visit
VRBPAC	Vaccines and Related Biological Products Advisory Committee
VV	Vaccinia Virus
VV-WR	Vaccinia Virus Western Reserve
WBC	White Blood Cell Count
WHO	World Health Organization
WOCBP	Women of Childbearing Potential

1.6 Protocol Synopsis

Title	A randomized, double-blind, placebo-controlled Phase II study to evaluate safety and immunogenicity of one and two doses of IMVAMUNE® smallpox vaccine in 56-80 year old vaccinia-experienced subjects
DMID Number	08-0019
Sponsor	Bavarian Nordic A/S<br>Hejreskovvej 10A<br>DK-3490 Kvistgård<br>Denmark
Principal Investigator	Prof. Richard N. Greenberg<br>VA staff physician, Lexington VA Medical Center, Professor of Medicine, The Belinda Mason Carden and Paul Mason Professor of HIV/AIDS Research and Education, University of Kentucky, School of Medicine, Department of Medicine, Room MN-672, 800 Rose Street, Lexington, KY 40536-0084, USA
Project phase	Phase II
Investigational drug	One dose IMVAMUNE® liquid-frozen, containing 1 x 10^8 tissue culture infectious dose 50% ($TCID_{50}$) Modified Vaccinia Ankara – Bavarian Nordic (MVA-BN®) virus per 0.5 ml.
Vaccine dosage	Each subject will receive either two vaccinations with 0.5 ml IMVAMUNE® vaccine dose containing 1 x 10^8 $TCID_{50}$ (Group 1) or one vaccination with 0.5 ml Placebo and one vaccination with IMVAMUNE® vaccine dose containing 1 x 10^8 $TCID_{50}$ (Group 2)
Route of administration	Each vaccination consists of one subcutaneous (s.c.) injection preferably in the non-dominant upper arm.

Vaccination schedule	Two vaccinations, first either with IMVAMUNE® or Placebo, second always with IMVAMUNE® will be administered to subjects 28 to 35 days apart.
Primary objective	To expand the IMVAMUNE® data base on safety in a vaccinia-experienced population 56-80 years of age after administration of either one or two doses of IMVAMUNE®.
Secondary objectives	To investigate the safety and reactogenicity of IMVAMUNE® in a vaccinia-experienced population 56-80 years of age after administration of either one or two doses of IMVAMUNE®<br><br>To compare the immunogenicity of IMVAMUNE® in a vaccinia-experienced population 56-80 years of age after administration of either one or two doses of IMVAMUNE®.
Study design	Two groups, randomized, double-blind, placebo-controlled. A stratified randomization with regard to the age ranges 56 - 70 and 71-80 years will be performed.<br><br>Group 1:<br>60 subjects will receive two s.c. vaccinations with 0.5 ml IMVAMUNE® vaccine containing 1 x 10^8 $TCID_{50}$ / dose according to a 0-4 week schedule (Day 0 / Day 28-35).<br><br>Group 2:<br>60 subjects will receive a first s.c. vaccination with placebo (0.5 ml saline) at Day 0, followed by a second s.c. vaccination with 0.5 ml IMVAMUNE® vaccine containing 1 x 10^8 $TCID_{50}$ at Day 28-35.<br><br>In a first step, 30 subjects in the age stratum 56-70 years will be enrolled. Safety data from these subjects up through Visit 2 will be reviewed by an independent Data Safety Monitoring Board (DSMB). If no safety concerns are identified, enrolment for the remaining subjects will be opened to volunteers up to 80 years of age.
Study duration	Seven study visits, up to 39 weeks for each subject.

Planned total sample size	The total sample size will be 120 with 60 subjects in each of the two groups. The study will be performed at up to four study centers in the US.
Statistical considerations	The statistical analysis will be done descriptively.<br>An analysis will be done for the overall study population. In addition, a stratified analysis will be performed for the 56-70 year old subject stratum and the 71-80 year old subject stratum.

Subject entry criteria

Inclusion criteria

1. Male and female subjects 56-70 years of age. If no safety concerns are identified upon review of the safety data from the first 30 subjects enrolled, the age range is extended up to 80 years.
2. Time since most current smallpox vaccination > 10 years.
3. The subject has read, signed and dated the Informed Consent Form (ICF), successfully completed (at least 90% correct [no more than 3 attempts allowed]) the test of understanding and has signed the Health Insurance Portability and Accountability Act (HIPAA) authorization form.
4. Women must have a negative serum pregnancy test at screening and negative urine pregnancy test within 24 hours prior to vaccination.
5. Women of childbearing potential (WOCBP) must have used an acceptable method of contraception for 30 days prior to the first vaccination, must agree to use an acceptable method of contraception during the study and must not plan to become pregnant for at least 28 days after the last vaccination. (Acceptable contraception methods are restricted to abstinence, barrier contraceptives, intrauterine contraceptive devices or licensed hormonal products).
6. Weight: ≥ 100 pounds (45.5 kg) and ≤ 330 pounds (150 kg).
7. White blood cells ≥ 2500/mm^3 and < 11,000/mm^3.
8. Absolute neutrophil count within normal limits.
9. Hemoglobin within normal limits.
10. Platelets within normal limits.
11. Adequate renal function defined as:
    - Urine protein ≤ +1 (by dip stick)
    - Serum creatinine within normal limits

12. Adequate hepatic function defined as:
    - Total bilirubin ≤ 1.5 x upper limit of normal (ULN) in the absence of other evidence of significant liver disease.
    - Aspartate aminotransferase (AST), alanine aminotransferase (ALT) and alkaline phosphatase ≤ 1.5 x ULN.
13. Cardiac troponin I < 2 x ULN.
14. Electrocardiogram (ECG) without clinically significant findings, e.g. any kind of atrioventricular or intraventricular conditions or blocks such as complete left or right bundle branch block, AV node block, QTc or PR prolongation, sustained atrial arrhythmias, sustained ventricular arrhythmia, 2 premature ventricular contractions (PVC) in a row, ST elevation consistent with ischemia.

Exclusion criteria

1. History of or active immunodeficiency or immuno-suppression caused by acquired or congenital diseases or caused by treatments such as chronic administration (> 14 days) of systemic, i.e. parenteral or oral, corticosteroids (> 5 mg prednisone [or equivalent] per day), radiation or immune-modifying drugs.
2. Periodic steroid injections, e.g. intraarticular, are not allowed within 30 days prior to the first vaccination and throughout the study until Visit 5 (V5).
3. Post organ transplant subjects whether or not receiving chronic immunosuppressive therapy.
4. Uncontrolled serious infection, i.e. not responding to antimicrobial therapy.
5. History of any serious medical condition, which in the opinion of the investigator would compromise the safety of the subject or prevent the subject from complying with study requirements.
6. History of or active autoimmune disease, e.g. Type I diabetes. Persons with vitiligo or thyroid disease taking thyroid hormone replacement are not excluded.
7. Skin cancer in the past six months. If treatment for skin cancer was successfully completed more than six months ago and the malignancy is considered to be cured, the subject may be enrolled. Subjects with history of skin cancer must not be vaccinated at the previous site of cancer.
8. Any other malignancy in the past five years. If treatment for cancer was successfully completed more than 5 years ago and the malignancy is considered to be cured, the subject may be enrolled.

9. Clinically significant hematological, renal, hepatic, pulmonary, central nervous, cardiovascular or gastrointestinal disorders which are not adequately controlled by medical treatment within the last 12 weeks before vaccination as judged by the site's Principal Investigator.
10. History of myocardial infarction, congestive heart failure with marked limitation of activity due to symptoms, e.g. walking short distances [20-100 m] (i.e. > Grade II according to the New York Heart Association), cardiomyopathy and stroke or transient ischemic attack in the past two years.
11. Uncontrolled high blood pressure defined as systolic blood pressure ≥ 150 mm Hg and/or ≥ diastolic blood pressure ≥ 100 mm Hg within the last six months.
12. Subjects with active coronary heart disease manifested by angina, even if on medication.
13. 25 % or greater risk of developing a myocardial infarction or coronary death within the next 10 years using the National Cholesterol Education Program's Risk Assessment Tool: http://hin.nhlbi.nih.gov/atpiii/calculator.asp
14. Clinically significant mental disorder not adequately controlled by medical treatment.
15. History of chronic alcohol abuse (40 g/day, e.g. 3 glasses of beer or 2 glasses of wine for at least six months) and/or intravenous drug abuse (within the last six months). Subjects with a history of other substance and/or alcohol abuse are also excluded if – in the opinion of the investigator – the abuse could prevent the subject from complying with study requirements.
16. History of allergic disease or reactions likely to be exacerbated by IMVAMUNE® or any component of the vaccine, e.g. tris(hydroxymethyl)-amino methane, chicken embryo fibroblast proteins, aminoglycosides (gentamycin).
17. History of anaphylactic shock or any severe allergic reaction to a vaccine requiring immediate treatment.
18. Subjects undergoing treatment for tuberculosis infection or disease.
19. Having received any vaccinations or planned vaccinations with a live vaccine within 30 days prior to or after study vaccination.
20. Having received any vaccinations or planned vaccinations with a killed vaccine within 14 days prior to or after study vaccination.

21. Administration or planned administration of immunoglobulins and/or any blood products during a period starting from three months prior to administration of the vaccine and ending at study conclusion.
22. Use of any investigational or non-registered drug or vaccine other than the study vaccine within 30 days preceding the first dose of the study vaccine or planned administration of such a drug during the study period.
23. Temperature ≥ 100.4°F (38.0°C) at the time of enrollment.
24. Any condition which might interfere with study objectives or would limit the subject's ability to complete the study in the opinion of the investigator.
25. Study personnel.

Study endpoints

Primary endpoint	Occurrence of any serious adverse events (SAEs) associated with the study vaccine occurring until the last active study visit (Visit 5).
Secondary endpoint (safety)	Occurrence of unsolicited non-serious adverse events (AEs) within 28 days after each vaccination: Severity, duration and relationship to vaccination.<br><br>Occurrence of any Grade 3 or 4 AEs associated with the study vaccine within 28 days after each vaccination.<br><br>Occurrence, relationship and severity of any cardiac events and/or any ECG change indicating a case of myo-/pericarditis at any time during the study.<br><br>Occurrence of solicited local adverse reactions within one week (Days 0-7) after each vaccination: Analysis of severity and duration.<br><br>Occurrence of solicited general AEs within one week (Days 0-7) after each vaccination: Analysis of severity, duration and relationship to vaccination.
Secondary endpoint (immunology)	Percentage of subjects with an ELISA response, i.e. either an appearance of antibody titers ≥ 50 in a vaccinia-specific Enzyme-linked immunosorbent assay (ELISA) for initially seronegative subjects, or an increase of the antibody titer compared to the baseline titer for subjects with a pre-existing antibody titer, at the individual peak response.

Percentage of subjects with an ELISA response at all individual blood sampling time-points.

Geometric Mean Titers (GMTs) measured by vaccinia-specific ELISA at the individual peak response.

GMTs measured by vaccinia-specific ELISA at all blood sampling time-points.

Percentage of subjects with a Plaque-reduction neutralization test (PRNT) response, i.e. either an appearance of antibody titers ≥ 6 in a vaccinia-specific PRNT for initially seronegative subjects, or an increase of the antibody titer compared to the baseline titer for subjects with a pre-existing antibody titer in the PRNT, at the individual peak response.

Percentage of subjects with a PRNT response at all individual blood sampling time-points.

GMTs measured by vaccinia-specific PRNT at the individual peak response.

GMTs measured by vaccinia-specific PRNT at all blood sampling time-points.

Pearson's correlation coefficient between antibody titers measured by ELISA and PRNT at each individual visit, and individual peak values. It is assumed that the data are log-normally distributed. Therefore, the titers will be log10 transformed for the correlation analysis.

1.7 Flow Chart

Phase	Screening	Active Study Phase					Follow up
Visit (V)	SCR	V 1	V 2	V 3	V 4	V 5	V FU
Day(s)	-28–1	0	V 1 +10-15	V 1 +28-35	V 3 +10-15	V 3 +28-35	V 3 +182–210
Informed consent, incl. HIPPA and test of understanding	X						
Medical history	X						
Check inclusion/ exclusion criteria	X	X		X[6]			
Complete physical exam (see 8.1.2)	X						
Targeted physical exam (see 8.1.3)		X	X	X	X	X	X
Vital signs	X	X	X	X	X	X	X
ECG[1]	X		X		X		
Review baseline signs & symptoms	X	X					
Review prior / concomitant medication	X	X	X	X	X	X	
Urine analysis[1]	X						
Safety laboratory[1]	X		X		X		
Pregnancy test[2]	X	X		X		X	
Sera sampling for antibody analysis		X	X	X	X	X	X
Randomization		X					
**Vaccination**		**X**		**X**[6]			
Handout of diary card[3]		X		X			
Collection of diary card			X		X		
AE reporting and documentation		X	X	X	X	X	X[4]
Blood sampling in ml[5]	15	8	23	8	23	8	8

1 At any other study visit, safety lab, urine analysis and/or ECG is only required if it is clinically indicated.
2 Urine or serum pregnancy test. At screening visit, a serum test must be performed.
3 The diary card should be completed daily for 7 days. If symptoms persist at Day 7, temperature/symptom measurements should be recorded each day until resolved.
4 SAEs only.
5 Amount of single blood draws: Safety laboratory: 15 ml; Antibody analysis: 8 ml. Total amount of blood taken during study: Maximum of 93 ml.
6 Please see Section 6.2: Contradictions and Precautions for further Study Vaccinations.

2. Background Information

In May 1980, the World Health Organization (WHO) declared the global eradication of smallpox. Since 1982, smallpox vaccination has not been required for international travelers' vaccination and International Certificates of Vaccination no longer include smallpox. As smallpox vaccination was deleted from routine vaccination schedules, production of the vaccine was no longer required.

Despite the fact that the WHO officially declared smallpox to be eradicated, a new threat exists due to the potential use of variola virus as an agent for biological warfare and/or bio-terrorism. Indeed, as mass vaccination programs halted more than 25 years ago, it is estimated that the majority of the world population has no existing immunity to smallpox, and as such, the release of this highly contagious virus would have devastating effects. As a consequence, there is an urgent need for a safe and efficacious vaccine to protect the public against smallpox.

2.1 First Generation Smallpox Vaccines

The original smallpox vaccines were based on a number of different vaccinia virus (VV) strains, e.g. Lister-Elstree strain recommended by the WHO and used primarily in Europe or the New York City Board of Health (NYCBH, Dryvax®) strain used in the United States. While these proved to be highly effective immunizing agents making the eradication of smallpox possible, they also showed considerable side effects. Besides local reactions with scab development and scarring, general symptoms observed frequently after smallpox vaccination have been fever, weakness, muscular pain, headache, swelling and soreness of local lymph nodes and rashes. Fever occurred in the majority of vaccinees, especially in small children: up to 70% developed temperatures of 100.4°F (38 °C), while 15-20% showed even higher temperatures. Apart from less dramatic and transient side effects like erythematous or urticarial rashes, severe and potentially fatal cutaneous complications of vaccinia vaccination include eczema vaccinatum and progressive vaccinia. Most feared are complications of the central nervous system, especially post-vaccinal encephalitis, which lead to death in 15-25% of cases and in 25% to neurologic sequelae (Goldstein et al. 1975; Lane et al. 1969; Lane et al. 1970). In Germany, the occurrence of neurological complications in primary vaccinees was reported in 1:20,000 to 30,000 vaccinees. Even though some countries such as the United States excluded high-risk individuals from vaccination, an average of seven persons a year still died from complications due to smallpox vaccination during the eradication campaign (McElwain 1972).

Another consideration for the discontinued use of the conventional smallpox vaccine is the world prevalence of human immunodeficiency virus (HIV) and acquired immunodeficiency syndrome (AIDS). Replication competent smallpox vaccines could be lethal if given to immune compromised individuals. A study published in 1991 (Guillaume et al. 1991) reported two cases of HIV infected individuals that received an experimental immunotherapy in form of paraformaldehyde fixed autologous T cells previously infected with recombinant VVs. Both patients were immune compromised and experienced necrotic skin lesions due to generalized vaccinia infections that led to death. However, complications following vaccinations with vaccinia can also occur in HIV infected individuals with T cell counts in the normal range and who are otherwise healthy (Redfield et al. 1987).

Traditionally, successful vaccination with a smallpox vaccine was assessed based on the formation of a vesicle ("take") at the inoculation site seven to nine days after vaccination. Recent clinical studies using Dryvax® confirmed a success rate by vesicle formation in vaccinia-naïve volunteers of 95 to 99% (Frey et al, 2002; ACAM2000™ Vaccines and Related Biological Products Advisory Committee [VRBPAC] Briefing Document, 2007).

2.2 Second Generation Smallpox Vaccines

Second generation smallpox vaccines are derived from first generation VV strains by plaque purification and manufactured in cell cultures according to modern Good Manufacturing Procedure (GMP) standards. Vaccination of individuals with these vaccines is performed in the same way as with first generation smallpox vaccines, namely by intradermal administration (scarification) of a single dose.

ACAM2000™ developed by Acambis Inc. is based on the Dryvax® NYCBH strain. In preparation of a Biologics License Application at the US Food and Drug Administration (FDA), Acambis conducted two pivotal Phase III clinical trials enrolling either vaccinia-naïve or vaccinia-experienced populations. The trials were designed to compare the safety, tolerability and efficacy of ACAM2000™ with Dryvax®. In total, the ratio of individuals in these trials receiving ACAM2000™ and Dryvax® was 3:1. Results were publicly made available in May 2007 (ACAM2000™ Vaccines VRBPAC Briefing Document, 2007).

Safety information available from these trials suggests that the non-serious adverse reactions were typical for injectable vaccines. The majority (99%) of subjects experienced at least one treatment-emergent AE after vaccination. The AEs most commonly reported fell into four distinct categories: reactions at the vaccination site, lymphadenitis, constitutional "flu-like" symptoms and minor gastrointestinal symptoms.

Of special importance, however, were a total of 10 serious cases of myo-/pericarditis that were reported within the ACAM2000™ development program. In a total vaccinia-naïve population of 1,675 subjects, these events occurred in seven subjects treated with ACAM2000™ (5.73 events per thousand vaccinations) and in three subjects having received Dryvax® (10.38 events per thousand vaccinations for a combined calculated incidence of 5.97 cases of myo-/pericarditis per thousand vaccinations). These figures represent an alarmingly high rate of potentially life-threatening serious AE following vaccination with a prophylactic vaccine.

Vaccine efficacy data were collected to demonstrate non-inferiority compared to Dryvax® based on the efficacy parameters of major cutaneous reaction ("take") rates and antibody titers against VV using PRNT in both trials.

Enrolling vaccinia-naïve subjects in one of the two trials, non-inferiority against Dryvax® could be shown for take rates, but not for antibody titers. On the contrary, for the study population of vaccinia-experienced subjects in the second Phase III trial, non-inferiority against Dryvax® could be determined for PRNT antibody titers, but not for take rates. Taken together, two of the four targeted efficacy measures were met in these trials.

Based on the safety and efficacy data collected in these pivotal Phase III trials, the FDA approved ACAM2000™ in September 2007 for use in vaccinia-naïve as well as vaccinia-experienced healthy populations, issuing a black box warning on the prescribing information for the special risks of this conventional smallpox vaccine.

2.3 Origin and Characteristics of IMVAMUNE®

VV is considered the best known member of the poxvirus family and the prototype live viral smallpox vaccine. VV replicates in the cytoplasm of the host cell, its DNA does not integrate into the host cell genome and it is non-oncogenic.

Modified Vaccinia Ankara (MVA) was derived from the serial passage of chorioallantois vaccinia Ankara virus (CVA), a VV strain used during the smallpox eradication program. During this passaging, MVA suffered a multitude of mutations within its genome, including six major deletions, resulting in the loss of 15% (30kbp) of original genetic information (Antoine et al. 1998). The deletions affected a number of virulence and host range genes (Antoine et al. 1998; Rosel et al. 1986; Meyer et al. 1991) and as a consequence, MVA exhibits a severely restricted host range in most mammalian cell types (Sutter & Moss 1992; Carroll & Moss 1997; Blanchard et al. 1998; Drexler et al. 1998). Although MVA exhibits a strongly attenuated replication in these cell types, its genes are efficiently transcribed with the block in viral replication being at the level of virus assembly and egress (Sutter & Moss 1992; Carroll & Moss 1997).

IMVAMUNE® has been derived from MVA-572 and is a highly attenuated, purified live vaccine produced under serum-free conditions in chicken embryo fibroblast (CEF) cells.

2.4 Summary of Non-clinical Data with IMVAMUNE®

Extensive in-house non-clinical and clinical studies have demonstrated that MVA-BN® (IMVAMUNE®) has superior characteristics compared to other MVA strains. Indeed, these studies have revealed that:

- IMVAMUNE® has a superior attenuation profile compared to other MVA strains and fails to replicate in human cell lines (Chaplin et al. 2002) and in severely immune compromised animals.
- A comprehensive Good Laboratory Practice safety package (acute toxicity, peri- and postnatal developmental toxicity, teratology and bio-distribution) has been performed that has demonstrated an excellent safety profile for IMVAMUNE® and the rapid clearance of the vaccine within 48 hours.

- A single vaccination with IMVAMUNE® induces the equivalent protection in mice challenged intranasally (i.n.) with 50x murine lethal dose 50 (MLD_{50}) VV Western Reserve strain (VV-WR) as traditional smallpox vaccines (Dryvax® and Elstree). Moreover, the protection afforded by IMVAMUNE® is robust and can protect the majority of vaccinated animals from a 500x MLD_{50} challenge with VV-WR (maximum challenge dose).
- Within 3 days of a single IMVAMUNE® vaccination, mice are protected from a lethal challenge (i.n.) with VV-WR, while animals vaccinated with traditional smallpox vaccines (e.g. Dryvax®) are only protected 10-14 days later.
- Non-human primates vaccinated with IMVAMUNE® are completely protected from a lethal monkeypox virus intratracheal challenge demonstrating an equivalent protection to traditional smallpox vaccines (Dryvax® and Lister-Elstree; Stittelaar et al. 2005)
- Mice lacking responsiveness to Interferon (IFN) type I (IFN alpha / beta) are highly susceptible to many viral diseases including Ectromelia Viruses (ECTV). IMVAMUNE® given at the same time as high lethal doses of ECTV protected highly immune compromised animals against death. In contrast, vaccination with Dryvax® killed these immune compromised animals.
- IMVAMUNE® given to Toll-like Receptor 9 deficient mice one day after exposure to a lethal dose of ECTV fully protected these immune compromised animals against death.

For more detailed information on non-clinical data please refer to the respective section of the Investigator's Brochure.

2.5 Clinical Profile of IMVAMUNE®

To date, 13 clinical studies evaluating the safety and immunogenicity of IMVAMUNE® have been completed or are ongoing. The majority of the studies have been / are being performed under Bavarian Nordic’s (BN) Investigational New Drug (IND) Application 11596, although three studies have been / are being conducted under IND 11229, filed by the Division of Microbiology and Infectious Diseases (DMID), National Institute of Allergy and Infectious Diseases (NIAID), National Institutes of Health (NIH). Two of the clinical studies (HIV-NEF-004 and HIV-POL-002) are evaluating two recombinant MVA-based HIV candidate vaccines (MVA-Nef and MVA-Polytope) in HIV infected subjects and utilize IMVAMUNE® as a control arm. To date, more than 2,600 subjects have been vaccinated with IMVAMUNE®, including risk groups for which conventional smallpox vaccines are contraindicated, such as HIV infected patients and patients with atopic dermatitis (AD). Table 1 provides an overview of completed and ongoing Phase I and II clinical trials in which subjects received IMVAMUNE®.

Table 1: Overview of completed and ongoing clinical trials with IMVAMUNE® (status: 10-Feb-2009)

Study Code	Phase	IND	Current Enrollment				Objective	Status
			Healthy	HIV infected	AD	HSCT*		
POX-MVA-001	I	Non-IND	86	-		-	Safety, dose-finding	Completed (Vollmar et al. 2006)
POX-MVA-002	I	11229 (NIH as sponsor)	75	-		-	Safety and immunogenicity comparison of IMVAMUNE® to Dryvax®	Completed (Frey et al. 2007)
POX-MVA-004	II	Non-IND	165	-		-	Safety and immunogenicity , dose-finding	Completed
POX-MVA-005	II	11596 (BN as sponsor)	564	-		-	Safety and immunogenicity, two doses vs. single dose	Completed
POX-MVA-007	I	11596	29	-	31		Safety and immunogenicity	Completed
POX-MVA-008	II	11596	275	-	322		Safety and immunogenicity	Ongoing
POX-MVA-009	I/II	11229	218				Immunogenicity, vaccination Days 0/7 vs. Days 0/14	Ongoing
POX-MVA-010	I/II	11596	60	91		-	Safety and immunogenicity	Completed
POX-MVA-011	II	11596	98	484		-	Safety and immunogenicity	Ongoing
POX-MVA-023	II	11596	152	-		-	Immunogenicity and safety	Ongoing
POX-MVA-030	I	11229				3	Safety and immunogenicity	Ongoing
HIV-NEF-004	II	Non-IND	-	26		-	HIV vaccine trial, IMVAMUNE® used in a comparator arm with 26 subjects	Completed
HIV-POL-002	I	Non-IND		10			HIV vaccine trial, IMVAMUNE® used in a comparator arm with 10 subjects	Reporting
Total Enrolment: 2689			1722	611	353	3		

*Persons who have undergone prior Hematopoietic Stem Cell Transplantation (HSCT)

2.5.1 Safety Overview of IMVAMUNE®

In all completed and ongoing clinical studies, vaccinations with IMVAMUNE® have shown to be generally safe and well tolerated. No cases of death, assessed as being even possibly related, have been reported for a subject in a clinical trial using IMVAMUNE®.

2.5.1.1 SAEs

To date, four SAEs assessed by the reporting investigators as being possibly related have been reported for IMVAMUNE®, two having occurred during the follow-up phase of the studies POX-MVA-010 and POX-MVA-005 and two events in the ongoing studies POX-MVA-011 and POX-MVA-008.

The first SAE concerns a 30 year old, healthy, white male subject diagnosed with “sarcoidosis”. The man began experiencing arthralgia approximately 70 days following the second vaccination with IMVAMUNE® and later on fever up to 38°C (100.4°F) combined with night sweats. The first symptoms occurred during antibiotic treatment of a urinary tract infection with Chlamydia trachomatis, however, these persisted for nearly a month at which time a thorax computer tomography was performed. The computer tomography results suggested the diagnosis of sarcoidosis and this was confirmed two weeks later via bronchoscopy and biopsy. The subject was treated symptomatically with ibuprofen over a period of four weeks and was last reported to be without any complaints. The investigator assessed the case to be “otherwise medically important” and possibly related to the vaccination, due to the fact that the cause of sarcoidosis is unknown. Upon review of all available data, the DSMB members assessed the event to be unlikely related to vaccination with the study drug and they expressed no concerns regarding continuation of the development program with IMVAMUNE®.

The second SAE of “congestive heart failure due to cardiomyopathy”, which led to hospitalization occurred 133 days following the second vaccination in a 30 year old, HIV infected, African American female subject who revealed several complicating factors in her medical history (foramen ovale repair as a child, right bundle branch block and left ventricular hypertrophy at screening) and concomitant medications (growth hormone releasing factor also in a clinical trial setting). Following extensive review of the complete documentation for this event, the members of the independent DSMBs reviewing the IMVAMUNE® development program have assessed that IMVAMUNE® and its recombinant products do not seem to pose an increased risk to subjects for developing cardiac events.

The third SAE reported to date with IMVAMUNE® involves a 39 year old, HIV infected, white female who was admitted to the hospital with complaints of dyspnea and non-productive cough for one day, also right ear/jaw pain, sinus and nasal congestion with pressure as well as headache. Based on the results of a chest x-ray, “simple pneumonia and pleurisy” were diagnosed. Following three days of antibiotic treatment in the hospital, she was discharged with complete resolution of symptoms. The investigator assessed the pneumonia to be possibly related to the study product, because the event began within one day after the second injection of vaccine and no other explanation was available. The subject was asymptomatic and had no abnormal physical

findings on the day of vaccination. Having a history of chronic obstructive pulmonary disease and HIV infection (her CD4 count four weeks prior to second vaccination was 299 cells/µl) combined with acute sinus and nasal congestion due to swimmer's ear starting the day prior to hospital admittance, BN believes these underlying respectively confounding diseases may represent more likely contributory factors in the development of this case of simple pneumonia and pleurisy. The event had resolved completely without sequelae already at the time of initial reporting. Following review by the DSMB members, no concerns were expressed. No additional follow-up on this case is expected.

The fourth SAE involves a 28 year old, healthy (no active or history of AD) female participant who experienced mild transitory ocular muscle paresis eight days following the second vaccination with IMVAMUNE®. Prior to onset of the event the subject was not feeling ill and she had tolerated the vaccinations well, having experienced only mild, transient, self-limiting fever, headache, nausea and myalgia following both doses of vaccine. She was not taking concomitant medications at any time during the study; intake of oral contraceptives was excluded as well. Serology tests confirmed that the subject was negative for hepatitis B and C as well as HIV. An ophthalmologist made the initial diagnosis and suggested the causality. The mild paresis causing dyplopia completely resolved after 26 days of treatment with carbamazepine and vitamin B complex. There was no remaining ocular mobility limitation and the subject’s vision returned to 20 / 20 in both eyes.

In the scientific literature numerous cases of optic neuritis have been reported following administration of rubella, measles, hepatitis B, influenza as well as anthrax vaccines. The ophthalmologist did not report that the optic nerve was involved in this case; however, rather the impairment seems to have been limited to the extraocular inferior rectal muscle. No other cases of paralysis or similar events have been observed following administration of IMVAMUNE®. Due to the temporal relationship as well as a lack of other obvious risk factors, BN nonetheless agrees that a causal relationship cannot be completely ruled out in this case. The DSMB members responsible for this trial have reviewed the available information and concluded that this event does not raise concerns which would necessitate putting the trial on hold.

2.5.1.2 Adverse Events / Reactions

Table 2 lists the undesirable effects assessed as having been at least possibly related to the study vaccine grouped by frequency which have been reported in the completed IMVAMUNE® clinical trials POX-MVA-001, POX-MVA-004, POX-MVA-005, POX-MVA-007 and POX-MVA-010. The majority of events is typical for modern injectable vaccines and was classified as being mild to moderate. All events resolved without sequelae.

Table 2: Adverse reactions reported in completed clinical trials (N=1,025 subjects) with IMVAMUNE® (status: 01-Feb-2009)

Very common (≥ 10%)	Administration site disorders: Pain, erythema, induration, swelling, pruritus.<br>Body as a whole – general disorders: Fatigue, headache, myalgia.<br>Gastrointestinal system: Nausea.
Common (≥ 1 - < 10%)	Administration site disorders: Warmth, hematoma, hemorrhage, inflammation.<br>Body as a whole – general disorders: Body temperature increased, chills.<br>Blood and the lymphatic system disorders: Lymphadenopathy.<br>Central and peripheral nervous system: Dizziness.
Uncommon (≥ 0.2 - < 1%)	Administration site disorders: Pigmentation change, irritation, paraesthesia, anesthesia, bruising, movement impairment.<br>Body as a whole – general disorders: Arthralgia, flushing, malaise, muscular weakness, axillary pain, neck pain, asthenia, non-cardiac chest pain, back pain, restlessness.<br>Gastrointestinal system: Diarrhea, abdominal pain, dry mouth.<br>Skin disorders: Rash, pruritus*, hyperhidrosis, dermatitis atopic*, urticaria, erythema.<br>Respiratory tract: Pharyngolaryngeal pain, nasopharyngitis, sinusitis.<br>Cardiac disorders: Palpitations, tachycardia.<br>Hepatobiliary disorders: Hepatic enzyme increased.<br>Eye disorders: Conjunctivitis.

* The two cases each of [generalized] pruritus and AD were reported in patients with active AD recruited in the trial specifically investigating subjects with atopic disorders, POX-MVA-007.

A complete listing of the exact frequencies of adverse drug reactions (ADRs) according to system organ class as well as a separate list of the possibly related reactions which have been documented only in single subjects reported so far in all completed IMVAMUNE® clinical trials is provided in the Investigator's Brochure.

In all recent clinical trials using IMVAMUNE®, particular attention has been placed on monitoring for cardiac signs and symptoms. These are defined in the clinical trial protocols as AEs of special interest or special interest AEs (SIAEs) and include:

- Any cardiac symptom determined to be clinically significant
- ECG changes determined to be clinically significant
- Cardiac enzymes elevated ≥ 2 x ULN

In the clinical trial POX-MVA-005 designed to closely monitor cardiac events in vaccinia-naïve and vaccinia-experienced subjects after vaccination with the IMVAMUNE® dose or placebo, 11 SIAEs were observed in 10 subjects (Table 3).

Table 3: SIAEs reported in Clinical Trial POX-MVA-005

Subject	Gender*	Age (years)	AE (Diagnosis)	Relationship to study vaccine
**Group 1 (vaccinia-naïve subjects, 2 vaccinations IMVAMUNE® / IMVAMUNE®)**				
287	Female	28	Sinus tachycardia	Unlikely related
317	Male	45	Tachycardia	Unlikely related
557	Male	35	Palpitations (for about 5 seconds). Subject known palpitations from years ago during sports for the first time.	Unlikely related
**Group 2 (vaccinia-naïve subjects, 2 vaccinations IMVAMUNE® / Placebo)**				
220	Female	26	Palpitations**	Possibly related
228	Male	32	Sinus tachycardia	Unlikely related
563	Female	25	Tachycardia (105 bpm)	Possibly related
**Group 3 (vaccinia-naïve subjects, 2 vaccinations Placebo / Placebo)**				
689	Male	30	Palpitations	Unlikely related
**Group 4 (vaccinia-experienced subjects, 1 vaccination IMVAMUNE®)**				
028	Male	46	Palpitations	Possibly related
028	Male	46	Palpitations	Possibly related
397	Female	37	Tachycardia reported by subject	Possibly related
411	Female	47	Palpitations	Unlikely related

* All subjects listed are Caucasians
** The report of "palpitations" in the Group 2 subject occurred after the second vaccination with placebo

Three SIAEs each were reported in the vaccinia-naïve groups receiving two IMVAMUNE® doses (Group 1) or one dose (Group 2) and one SIAE was reported in a placebo subject from Group 3. Among the vaccinia-experienced subjects in Group 4, four SIAEs were reported. These included 6 cases of palpitations, 3 cases of tachycardia and 2 cases of sinus tachycardia. 5 events (3 cases of palpitations and 2 reports of tachycardia) were assessed by the investigator to be possibly related to the study vaccine. Two of these possibly related SIAEs occurred in two female subjects in Group 2, one event of tachycardia in these subjects occurred after the placebo injection. The remaining three possibly related SIAEs occurred in Group 4 in one male (2 reports of palpitations) and one female subject (tachycardia). Extensive work-up of these subjects ruled out any clinical significance of these events.

In summary, all available study data from clinical trials using IMVAMUNE® have revealed no trends for unexpected and/or serious adverse reactions due to the investigational product. The large pool of available data from over 2,300 subjects vaccinated with IMVAMUNE® confirms the encouraging safety profile of this next generation smallpox vaccine candidate.

2.5.2 Immunogenicity Overview of IMVAMUNE®

Dose finding for IMVAMUNE® performed in Phase I and II trials using doses of 1 x 10^6, 2 x 10^7, 5 x 10^7 or 1 x 10^8 $TCID_{50}$ consistently showed a dose-response for all immunological parameters following vaccination (studies POX-MVA-001, -002 and -004). A dose response was also observed in terms of the magnitude and timing of the humoral immune response. While the results from these studies revealed all doses of IMVAMUNE® had been well tolerated, the superior dose in terms of magnitude and timing (earliest onset) of the antibody response was clearly the higher dose of IMVAMUNE® (1 x 10^8 $TCID_{50}$). Based on these data, the dose for vaccination with IMVAMUNE® has been determined to be 1 x 10^8 $TCID_{50}$ for both, healthy and at-risk populations.

Table 4 gives an overview of the level of protection as measured by seroconversion (using ELISA and PRNT) assessed in vaccinia-naïve subjects that were vaccinated with the optimal dose (1 x 10^8 $TCID_{50}$). Following a single vaccination with IMVAMUNE®, the seroconversion rates for healthy subjects (using the validated ELISA) were between 81 and 100% by day 28 post vaccination in group sizes varying from 14 to 183 subjects. Moreover, a seroconversion rate of 71% to 100% was already observed by day 14 post vaccination in healthy subjects. This confirms the rapid immune induction within 7 to 10 days following a vaccination with IMVAMUNE® also observed in non-clinical studies (mice and non-human primates).

Similar seroconversion rates were observed in people diagnosed with AD with 93% and 100% of the subjects having detectable antibodies to vaccinia by day 14 and 28 post vaccination (POX-MVA-007).

Cellular immune responses were analyzed in various trials using intracellular cytokine staining detecting vaccinia-specific IFN-γ producing CD4+/CD8+ T cells. These data also showed a strong, dose-dependent cellular immune response.

Clinical studies often included individuals with previous vaccination against smallpox in the past. IMVAMUNE® was able to stimulate the memory T and B cell responses induced by a previous smallpox vaccination using traditional vaccines (POX-MVA-001).

In study POX-MVA-002 (DMID 02-017) the immune responses induced by IMVAMUNE® were compared to the traditional smallpox vaccine, Dryvax®. Nearly 100% of the subjects had seroconverted by Day 10-14 following a single administration of IMVAMUNE® when analyzed by ELISA. GMTs measured via ELISA of the 1 x 10^8 $TCID_{50}$ IMVAMUNE® dose groups were comparable to Dryvax® with a strong increase in GMTs 14 days after the second vaccination. In addition, IMVAMUNE® and Dryvax® induced similar levels of T cell immunity with all subjects having detectable T cell responses 26-30 days following single vaccinations.

This study also examined the effect of vaccination with IMVAMUNE® on challenge with Dryvax®. Clearly, the immune response induced by IMVAMUNE® reduced the ability of the Dryvax® VV to replicate, resulting in reduced titers and a more rapid clearance and recovery of the vaccine lesion (Figure 1).

Table 4 Summary of ELISA specific seroconversion rates in studies involving healthy vaccinia-naïve subjects following IMVAMUNE® ($1x10^8$ $TCID_{50}$ SC)

		Percentage Seroconverters								
Days Post Vaccination		0	14	28[6]	42	56	112	182	1 yr	2 yr
POX-MVA-001 (Group C, N=16)	n	16	-	16	15	-	-	-	12	7
	ELISA[1,2]	6	-	81	100	-	-	-	42	43
	PRNT[1,2]	19	-	56	80	-	-	-	-	-
POX-MVA-002 (Group C, N=15)	n	15	15	15	15	14	13	-	-	-
	ELISA[2]	0	100	100	100	100	100	-	-	-
	PRNT[2]	7	73	73	100	100	100	-	-	-
POX-MVA-002 (Group E, N=15)	n	15	14	14	13	13	12	9	7	-
	ELISA[2]	13	93	100	100	100	100	100	100	-
	PRNT[2]	0	93	71	100	100	100	100	100	-
POX-MVA-004 (N=52)	n	52	-	52	52	-	-	-	-	27
	ELISA[2]	0	-	94	100	-	-	-	-	56
	PRNT[2,3]	0	-	10	71	-	-	-	-	67
POX-MVA-005 (N=183) FAS[5]	n	183	182	180	176	178	-	178	-	-
	ELISA[4]	8	71	89	99	99	-	73	-	-
	PRNT[4]	3	45	57	89	86	-	65	-	-
POX-MVA-007 (N=15)	n	15	15	15	15	15	-	15	-	-
	ELISA[2]	0	93	100	100	100	-	100	-	-
	PRNT[2]	7	73	80	100	93	-	93	-	-
POX-MVA-010 (N=25)	n	25	23	24	25	25	-	22	-	-
	ELISA[4]	16	78	96	100	96	-	86	-	-
	PRNT[4]	8	83	71	96	96	-	82	-	-

N=number of subjects in a group; n=number of subjects analyzed; - no blood sample taken.

1 Assays (ELISA and PRNT) were not validated and subsequently replaced.
2 Seropositivity rates reflect the rate of ELISA or PRNT above the cut-off value of the assay.
3 PRNT not validated.
4 For subjects that were seronegative prior to vaccination, seroconversion was defined as ELISA or PRNT value above the cut-off value of the assay. For subjects that were seropositive prior to vaccination, seroconversion was defined as at least a two-fold increase over pre-vaccination titer.
5 POX-MVA-005 shows FAS data whereas for other studies the PPS data are presented.
6 Vaccination schedule: Day 0 initial vaccination, Day 28 second vaccination.

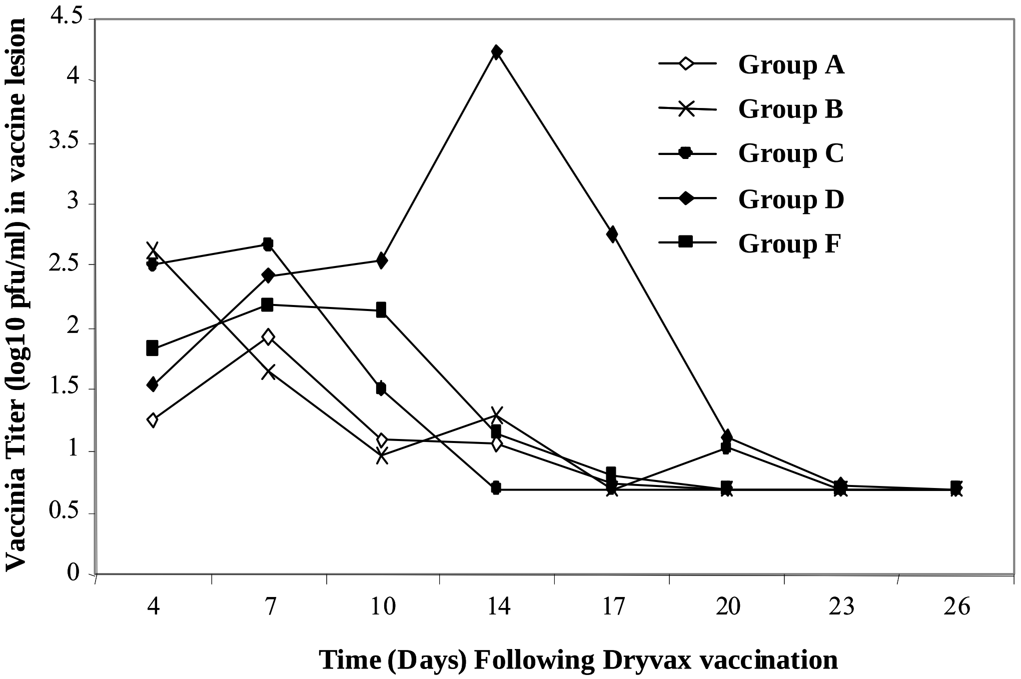

Figure 1: Vaccinia Virus Titers in the Vaccine Lesions Following Vaccination with Dryvax®. Healthy subjects (N=90) were randomized into 6 groups. Groups A, B and C all received two s.c. vaccinations with IMVAMUNE® on Day 0 and 28 with either 2 x 10^7, 5 x 10^7, or 1 x 10^8 $TCID_{50}$. Group F also received two vaccinations using the same schedule, although IMVAMUNE® (1 x 10^8 $TCID_{50}$) was administered intramuscularly. On Day 112, all subjects in Groups A, B, C and F were vaccinated with Dryvax® via scarification. Group D subjects received placebo on Day 0 and 28, followed by Dryvax® on Day 112. The vaccinia titers were determined in the vaccine lesion at different time points following the Dryvax® vaccination.

Interestingly, the efficacy of IMVAMUNE® was not dependent on the dose or route. This is especially surprising when the neutralizing antibody titers at the time of the Dryvax® vaccination (Day 112) are compared between the groups (Table 5). Even though some people did not show a detectable neutralizing antibody titer at Day 112 (the day of the Dryvax® challenge), there was no difference in the measured efficacy (clearance of Dryvax® and healing of the vaccine lesion). In contrast, at the time of the Dryvax® vaccination (Day 112) there was a 100% seroconversion by ELISA (total antibody response) in all IMVAMUNE® vaccinated groups. Therefore, the results from this trial have demonstrated that the total antibody (Immunoglobuline G [IgG]) measured by ELISA is a better indication for assessing the efficacy of IMVAMUNE® (VV titers and vaccine lesion healing time after Dryvax® scarification) than neutralizing antibodies. These results support the preclinical data that have also shown that there is not a single correlate of protection against lethal challenges with orthopox viruses and that protection can occur in the absence of detectable neutralizing antibody titers, while total antibodies (IgG) measured by ELISA are the best predictive immunological parameter for protection. This probably reflects the fact that total antibody titers are highly correlated to neutralizing titers in animals and humans (Figure 2) and that the ELISA is a more sensitive assay compared to the PRNT, supporting the use of the ELISA as the primary endpoint in pivotal non-clinical and clinical studies.

Table 5: Humoral Immune Responses Following Vaccination with Various Doses of IMVAMUNE® and Challenge with Dryvax® (Data from POX-MVA-002)

Group	Seroconversion rates (%)									
	Day 0 (1st vaccination)		Day 14		Day 28 (2nd vaccination)		Day 42		Day 112 Dryvax® challenge	
	ELISA[1]	PRNT[2]	ELISA	PRNT	ELISA	PRNT	ELISA	PRNT	ELISA	PRNT
A	0	0	93	53	93	20	100	100	100	54
B	13	7	87	67	93	47	100	93	100	92
C	0	7	100	87	100	53	100	87	100	85
F	7	7	100	73	100	67	100	100	100	94

[1] Seroconversion classified as ELISA titer ≥ 50
[2] Seroconversion classified as PRNT titer ≥20
Group A: 2 x 10^7 $TCID_{50}$ IMVAMUNE® (s.c.) followed by Dryvax® on Day 112
Group B: 5 x 10^7 $TCID_{50}$ IMVAMUNE® (s.c.) followed by Dryvax® on Day 112
Group C: 1 x 10^8 $TCID_{50}$ IMVAMUNE® (s.c.) followed by Dryvax® on Day 112
Group F: 1 x 10^8 $TCID_{50}$ IMVAMUNE® (i.m.) followed by Dryvax® on Day 112

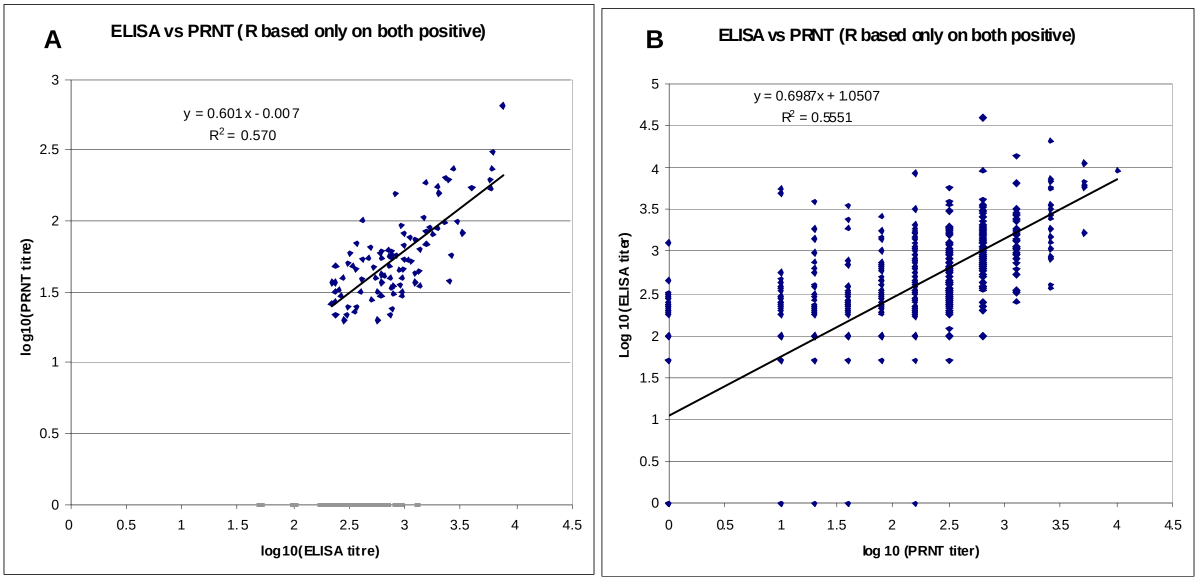

Figure 2: ELISA/PRNT Correlation Analysis from A) POX-MVA-004 and B) POX-MVA-010)
ELISA titers in both studies were determined using a validated ELISA and the titers were determined by linear regression from the plotted OD versus sera dilution and expressed at the GMT. For POX-MVA-004 (A), the PRNT titers were also determined by linear regression (plaque count versus sera dilution, i.e. the exact point in the curve where the virus is neutralized by 50%) at BN and expressed as GMT. However, for POX-MVA-010 (B), the PRNT titers were determined by Focus Diagnostics Inc. as the first dilution that resulted in a 50% reduction in the total number of plaques and resulted in discrete dilutional titers (e.g. 10, 20, 40, 80, 160 etc).

Additional detailed information on the clinical development of IMVAMUNE® is provided in the Investigator's Brochure.

2.6 Rationale

Studies performed with IMVAMUNE® include vaccinia-naïve as well as vaccinia-experienced subjects, with a clear age difference between the two populations due to the worldwide discontinuation of compulsory vaccination programs against smallpox in the early 1980s. Results from these studies that included vaccinia-experienced subjects have shown that one dose of IMVAMUNE® can boost pre-existing immune responses originally generated by vaccination with conventional smallpox vaccines during the eradication program in subjects up to 55 years of age.

However, there are no data available yet to show if the vaccination with IMVAMUNE® can elicit a comparable immune response even in subjects which are older than 55 years. It is known that the immune function decreases with increased age of human subjects. One of the most striking changes that occur during normal human aging is known as immunosenescence, a progressive and overall diminution of immune functions that affect all cells and organs of the innate and adaptive immune system. As a hallmark of human aging, the progressive involution of the thymus leads to a disturbed balance and function of naïve, memory and effector T cells, thus promoting a latent pro-inflammatory status in the elderly. This situation manifests in clinically relevant implications such as poor overall immune responses, decreased ability to control infectious disease and diminished response to vaccinations [Gruver et al. 2007; Pfister and Saviano, 2008].

To overcome this lack of data, it is intended to evaluate the safety and immunogenicity in an elderly population. This study will target a population 56-80 years of age to expand the IMVAMUNE® data set in vaccinia-experienced subjects. The primary goal is to generate for the first time data on IMVAMUNE® in an elderly population while comparing two different vaccination schedules (one or two IMVAMUNE® vaccinations) with regard to safety and immunogenicity.

A close monitoring of physical condition, vital signs and blood parameters will collect a robust set of data in the elderly population to confirm the previously observed excellent safety profile of IMVAMUNE® in healthy as well as immunocompromised populations. Data from previous trials have shown that IMVAMUNE® is similarly well tolerated in vaccinia-experienced compared to vaccinia-naïve subjects.

This study will also collect humoral immunogenicity data in vaccinia-experienced subjects using validated PRNT and ELISA assays to show that one dose of IMVAMUNE® can increase antibody titers compared to baseline. This has already been shown in a recently completed Phase II trial (POX-MVA-005) where a single vaccination with IMVAMUNE® resulted in a booster response in nearly 100% of 200 healthy 18-55 year old vaccinia-experienced subjects demonstrated by an increase of anti-vaccinia titers.

Since positive baseline titers in vaccinia-experienced subjects can be considered protective as they have been generated by previous vaccination with conventional smallpox vaccines, any increase or re-appearance of titers based on immunological memory may be considered to be a reasonable indication of immunity. Taking into consideration that elderly subjects may respond less strongly to vaccines compared to younger people, this study additionally intends to evaluate

whether one vaccination is sufficient to generate a measurable booster response or whether a second dose might be needed.

2.7 Study Population

Women and men aged 56 to 70 years will initially be recruited for enrolment into this study. After recruitment of 30 subjects and review of safety data from these subjects through Visit 2 by the DSMB has revealed no safety concerns, subjects aged 56 to 80 years meeting all of the in- and exclusion criteria will be enrolled.
Women and members of minorities and their subpopulations will be included in this clinical study if volunteering.

2.8 Benefit/Risk Assessment

2.8.1 Risks for Study Participants

Preclinical data with IMVAMUNE® in mice and rats have revealed no special hazard for humans based on conventional studies of safety.

Over 1,700 healthy and more than 950 subjects with underlying conditions like HIV infection, AD or hematopoetic stem cell transplantation have been vaccinated in clinical trials using either IMVAMUNE® (throughout the development program for an improved smallpox vaccine) or MVA / MVA-BN® as a vector (with HIV specific recombinant inserts or inserts to be used as a melanoma vaccine).

Based on the present experience with IMVAMUNE® and MVA-based vaccines, adverse reactions to IMVAMUNE® are expected to be comparable to typical adverse reactions seen with other modern vaccines.

As with all injected vaccines, there is a risk of an allergic reaction or an anaphylactic event, although this has never been observed with IMVAMUNE®. Study center staff will watch volunteers for 30 minutes after each vaccination and in case a severe allergic reaction should occur, appropriate medical treatment and supervision will be readily available.

The severe and life-threatening adverse reactions such as progressive vaccinia, eczema vaccinatum, generalized vaccinia and inadvertent inoculation that are observed after the administration of conventional smallpox vaccines are due to the replication of the vaccinia strains. IMVAMUNE® is replication incompetent in human cells and therefore has a better safety and tolerability profile and as a consequence presumably cannot induce the severe side effects such as progressive vaccinia which is associated with replication competent vaccinia viruses. Apart from the better safety profile with regard to severe reactions, the available clinical experience with IMVAMUNE® shows that it is generally better tolerated (e.g. local reactions) than conventional smallpox vaccines.

Based on the comprehensive data available for IMVAMUNE®, the risk for the study participants is expected not to be higher than the risk observed with other modern vaccines. The main risk is the development of local reactions at the vaccination site, e.g. erythema, pain, swelling and induration.

Blood drawing may cause discomfort, bruising or light-headedness. Rarely, a blood draw may result in infection at the site where the blood is taken.

2.8.2 Benefits for Study Participants

Study participants will contribute significantly to the development of a safer smallpox vaccine, which is a benefit for society in view of a potential threat following deliberate release of smallpox virus. There will be no direct benefit to the study participants. Based on the current immunogenicity and efficacy data collected in non-clinical and clinical studies, study participants are expected to acquire protection against smallpox infection.

Analysis of the samples collected will not directly benefit the subject. BN may learn more about smallpox and other diseases: how to prevent them, how to treat them, or how to cure them.

3. Objectives

The primary objective of the trial is:
to expand the IMVAMUNE® data base on safety in a vaccinia-experienced population 56-80 years of age after administration of either one or two doses of IMVAMUNE®.

The secondary objectives of the trial are:
to investigate the safety and reactogenicity of IMVAMUNE® in a vaccinia-experienced population 56-80 years of age after administration of either one or two doses of IMVAMUNE®

to compare the immunogenicity of IMVAMUNE® in a vaccinia-experienced population 56-80 years of age after administration of either one or two doses of IMVAMUNE®.

4. Study Design

4.1 Experimental Design

This is a randomized, double-blind, placebo-controlled study. A stratified enrolment with regard to the age strata 56–70 and 71–80 years will be performed. In a first step 30 subjects in the stratum 56–70 years of age will be enrolled. Subsequently, the DSMB will perform a safety review of the data collected through Visit 2 from these subjects in this age cohort. If the DSMB does not identify a safety concern precluding continuation of the study as planned, enrolment for the remaining subjects will be opened to include volunteers up to 80 years of age.

Group 1 60 subjects will receive two s.c. vaccinations with 0.5 ml IMVAMUNE® vaccine containing 1 x 10^8 $TCID_{50}$ / dose according to a 0-4 week schedule (Day 0 / Day 28-35)

Group 2 60 subjects will receive a first s.c. vaccination with placebo (0.5 ml saline) at Day 0, followed by a second s.c. vaccination with 0.5 ml IMVAMUNE® vaccine containing 1 x 10^8 $TCID_{50}$ at Day 28-35

Visit	SCR	V1<br>1st Vacc.	V2	V3<br>2nd Vacc.	V4	V5	V FU
Day(s)	-28–1	0	V1<br>+ 10–15	V1<br>+ 28–35	V3<br>+10–15	V3<br>+ 28–35	V3<br>+182–210

4.2 Description of Study Procedures

The study will be conducted according to the Flow Chart (see 1.7 Flow Chart).

Visits should be scheduled within the specified intervals. The total amount of blood drawn within the study will be a maximum of 93 ml.

4.2.1 Screening Phase

Informed consent procedures will be performed at the screening visit prior to any study procedures. Potential subjects will be presented an overview of the study by the PI or a medically qualified designee (i.e. study coordinator). This includes thoroughly reviewing the ICF and HIPAA authorization individually with each potential subject, paying close attention to the study visit schedule, required evaluations and procedures. After review of these documents and clarification of all questions, each subject agreeing to participate in the study will sign and date the ICF and HIPAA authorization. A written test of understanding will then be performed to evaluate the potential subject's understanding of the study. Potential subjects may make up to three attempts to pass the test and must answer at least 90% of questions correctly in order to proceed with the screening process.

After the ICF and HIPAA have been signed, and the test of understanding has successfully been passed, subjects will enter a screening period of up to four weeks. The following assessments / examinations must be performed at screening:

Visit SCR (Days -28 to -1)
• Informed consent procedure including HIPAA and test of understanding<br>• Review medical history (Section 8.1.1)<br>• Review inclusion/exclusion criteria (Section 5.2 and 5.3)<br>• Complete physical examination including evaluation of body weight (Section 8.1.2)<br>• Evaluation of vital signs (Section 8.2.1.1)<br>• Review baseline signs & symptoms (Section 9)<br>• Perform baseline ECG<br>• Urine analysis<br>• Serum pregnancy test (if applicable)<br>• Safety laboratory (Section 8.2.1.3)<br>• Review prior/concomitant medication (Section 7.7)

If a subject was screened and could not be enrolled because of a certain transient condition (e.g. abnormal lab value due to an acute condition or a missing lab evaluation due to mishandling of the sample), then the subject may be re-screened and the respective tests have to be repeated as a "partial" re-screening rather than a full re-screening. The re-screening visit must be within the 28 days window started by the first screening visit and the window must not be exceeded.

If a subject could not be enrolled due to other circumstances (e.g. washout period for a vaccine or medication which represents a temporary exclusion criterion) or the 28 day period is exceeded, a complete re-screening assessment including physical examination, lab examination, ECG must be performed. The clock then re-starts at the re-screening visit with day -28 before 1st vaccination.

4.2.2 Active Study Phase

As soon as a subject passes all screening evaluations successfully, Visit 1 (V1) will be scheduled. The procedures performed at V1 and all following visits are listed below. Blood draws and all other tasks mentioned in the list above the actual vaccination must always be performed prior to vaccination.
The randomization of the study subjects to one of the two study groups will be performed as outlined in Section 7.4.

Each immunization consists of one dose of 1 x 10^8 $TCID_{50}$ IMVAMUNE® vaccine or one dose Placebo, respectively, administered s.c. preferably in the non-dominant upper arm.

Following vaccination subjects will be kept under close observation at the study site for at least 30 minutes, with appropriate medical treatment readily available in case of a rare anaphylactic reaction following the administration of vaccines.

Any AEs that occur during or after vaccination will be recorded. Subjects will be given oral and written instructions for completing a daily diary for 7 days following each vaccination. They will also be provided with a thermometer and a ruler for measuring the body temperature and any possible local reactions at the vaccination site. The subjects will record solicited and unsolicited AEs occurring on the vaccination day and the seven days following each vaccination on their diary card. If symptoms persist at Day 7, temperature/symptom measurements should continue to be recorded each day until resolved.

Visit 1 (Day 0)
• Review and verify inclusion/exclusion criteria (Sections 5.2 and 5.3)<br>• Review baseline signs & symptoms (Section 9)<br>• Urine or serum pregnancy test (if applicable)<br>• Review prior/concomitant medication (Section 7.7)<br>• Targeted physical examination (Section 8.1.2)<br>• Evaluation of vital signs<br>• Sera sampling for anti-vaccinia antibody analysis<br>• Randomization (Section 7.4)<br><br>**All tasks mentioned above must always be performed prior to vaccination**<br>• 1st Vaccination<br>• Handout of diary card for 1st vaccination, a ruler and a thermometer<br>• Recording and documentation of AEs/SAEs (Section 8.2.2 and Section 9)

Visit 2 (Visit 1 + 10–15 days)
• Safety laboratory<br>• ECG<br>• Sera sampling for anti-vaccinia antibody analysis<br>• Targeted physical examination<br>• Evaluation of vital signs (Section 8.2.1.1)<br>• Review concomitant medication<br>• Collection of the diary card and review together with subject<br>• Recording and documentation of AEs/SAEs (Section 8.2.2 and Section 9)

Visit 3 (Visit 1 + 28–35 days)
• Review and verify inclusion / exclusion criteria (Please see Section 6.2: Contradictions and Precautions for further Study Vaccinations<br>• Urine or serum pregnancy test (if applicable)<br>• Sera sampling for anti-vaccinia antibody analysis<br>• Targeted physical examination<br>• Evaluation of vital signs (Section 8.2.1.1)<br>• Review concomitant medication<br>**All tasks mentioned above must always be performed prior to vaccination**<br>• 2nd Vaccination<br>• Handout of diary card for 2nd vaccination and if needed, a ruler and thermometer<br>• Recording and documentation of AEs/SAEs (Section 8.2.2 and Section 9)

Visit 4 (Visit 3 + 10–15 days)
• Safety laboratory<br>• ECG<br>• Sera sampling for anti-vaccinia antibody analysis<br>• Targeted physical examination<br>• Evaluation of vital signs (Section 8.2.1.1)<br>• Review concomitant medication<br>• Collection of diary card and review together with subject<br>• Recording and documentation of AEs/SAEs (Section 8.2.2 and Section 9)

Visit 5 (Visit 3 + 28–35 days; conclusion of active study phase)
• Targeted physical examination<br>• Evaluation of vital signs (Section 8.2.1.1)<br>• Urine or serum pregnancy test (if applicable)<br>• Sera sampling for anti-vaccinia antibody analysis<br>• Review concomitant medication<br>• Recording and documentation of AEs/SAEs (Section 8.2.2 and Section 9)

4.2.3 Unscheduled Visits

If clinically indicated, additional visits may be necessary between scheduled visits. Unscheduled visits may be scheduled to repeat laboratory testing or physical exams due to a new development. These visits will be identified with letters added to the visit number to define the sequence of the visits until the next scheduled one (e.g. 1A, 1B etc. will indicate extra visits between Visit 1 and Visit 2).

4.2.4 Follow-up Phase

To obtain long-term safety and immunogenicity data of the IMVAMUNE® vaccination(s), the subject has to return for a FU examination 182–210 days (26–30 weeks) after the last vaccination (V3).

Visit FU (Visit 3 + 182–210 days; Follow-up visit)
• Targeted physical examination including evaluation of vital signs<br>• Sera sampling for anti-vaccinia antibody analysis<br>• Recording and documentation of SAEs (Section 9)

4.3 Study Duration

The total duration of the study for each subject from screening visit until follow-up visit will be up to 39 weeks.

All subjects receiving an IMVAMUNE® vaccination will be followed for at least 26 weeks. It is expected to recruit subjects within a reasonably short time, to limit the total study duration to 12 months.

4.4 Study Halting Rule

A temporary halting or termination for the study as a whole can be requested by the DSMB at any time during the study. Any member of the DSMB, one of the investigators, BN Safety Officer, or DMID Medical Officer can request a DSMB review based on any observation. Possible triggers for an ad hoc DSMB review include the occurrence of an SAE, an unexpected Grade 3 or higher systemic reaction or lab toxicity assessed to be at least possibly related to the administration of IMVAMUNE®. These parameters are not all-inclusive and other AEs could occur that would trigger a DSMB review as well.

Any definitely related SAE would lead to an immediate halting of the study until the DSMB has the opportunity to review all available safety data and provide a recommendation regarding further conduct of the study.

4.5 DSMB

The DSMB is an independent board that oversees the safety of subjects participating in the study. The members of the DSMB are selected by BN and the DMID in accordance with the DMID guidelines. DSMB members must be independent, i.e. not involved as investigators in one of the ongoing IMVAMUNE® studies. The primary responsibilities of the DSMB are to periodically review and evaluate the accumulated study data for participant safety, study conduct and progress, and make recommendations to BN, the DMID and the Coordinating and Principal Investigator(s) concerning the continuation, modification, or termination of the trial program. The DSMB considers study specific data as well as relevant background knowledge about the disease, test agent, and subject population under study. A separate charter describes in detail relevant operational procedures, communication pathways, roles and responsibilities of the DSMB.

In case an event occurs which fulfills the study halting criteria the DSMB will review the event in a timely manner and give a recommendation to BN, the DMID and the Coordinating and Principal Investigator(s) to halt, resume or terminate the study participation of the affected subject and/or the study as a whole.

4.6 Interim Safety Review

A blinded interim safety review will be performed after an initial enrolment of a total of 30 subjects in the age stratum from 56 to 70 years. Enrolment of 56 to 70 year old subjects will continue throughout the interim safety review.

For the interim safety review the DSMB will be provided with a brief report that describes short term toxicities (AEs, abnormal ECG findings and laboratory parameters) up through V2 following the first vaccination of the first 30 subjects. If the DSMB has any safety concerns, unblinded information will be made available to them.

Under the condition that no safety concerns are identified during the review of the interim data, subjects in the age stratum from 71 to 80 years may then also be enrolled.

5. Selection of Subjects

5.1 Recruitment Procedure

In total 120 subjects will be enrolled. The first 30 subjects have to be in the age stratum from 56 to 70 years. If there are no concerns identified by the DSMB at the time of the interim safety review, enrolment for the remaining subjects will be opened to include volunteers up to 80 years of age. A minimum (30) of these 90 subjects must be in the age stratum from 71 to 80 years.

After signing the Informed Consent, subjects undergo screening procedures to check eligibility regarding inclusion/exclusion criteria as defined in Section 4.2. In the event of a screening failure secondary to mild or limited acute illness or abnormal laboratory values, the subject may be re-screened after resolution of the event. Re-screening may require only an additional blood draw or a complete re-screening evaluation, depending on the circumstances of and the time interval from the initial screening failure. Partial re-screening is to be done within the 28 day screening period, while complete re-screening is required in case the 28 day screening period has been exceeded.

The investigator will keep a log of subjects screened for the study, but not enrolled, and provide the reason for exclusion.

BN will be informed about every subject entered in the study in a weekly status report.

5.2 Inclusion Criteria

1. Male and female subjects 56-70 years of age. If no safety concerns are identified upon review of the safety data from the first 30 subjects enrolled, the age range is extended up to 80 years.
2. Time since most current smallpox vaccination > 10 years.
3. The subject has read, signed and dated the Informed Consent Form (ICF), successfully completed (at least 90% correct [no more than 3 attempts allowed]) the test of understanding and has signed the Health Insurance Portability and Accountability Act (HIPAA) authorization form.
4. Women must have a negative serum pregnancy test at screening and negative urine pregnancy test within 24 hours prior to vaccination.
5. Women of childbearing potential (WOCBP) must have used an acceptable method of contraception for 30 days prior to the first vaccination, must agree to use an acceptable method of contraception during the study and must not plan to become pregnant for at least 28 days after the last vaccination. (Acceptable contraception methods are restricted to abstinence, barrier contraceptives, intrauterine contraceptive devices or licensed hormonal products).
6. Weight: ≥ 100 pounds (45.5 kg) and ≤ 330 pounds (150 kg).
7. White blood cells ≥ 2500/mm^3 and < 11,000/mm^3.
8. Absolute neutrophil count within normal limits.
9. Hemoglobin within normal limits.
10. Platelets within normal limits.

11. Adequate renal function defined as:
    - Urine protein ≤ +1 (by dip stick)
    - Serum creatinine within normal limits
12. Adequate hepatic function defined as:
    - Total bilirubin ≤ 1.5 x upper limit of normal (ULN) in the absence of other evidence of significant liver disease.
    - Aspartate aminotransferase (AST), alanine aminotransferase (ALT) and alkaline phosphatase ≤ 1.5 x ULN.
13. Cardiac troponin I < 2 x ULN.
14. Electrocardiogram (ECG) without clinically significant findings, e.g. any kind of atrioventricular or intraventricular conditions or blocks such as complete left or right bundle branch block, AV node block, QTc or PR prolongation, sustained atrial arrhythmias, sustained ventricular arrhythmia, 2 premature ventricular contractions (PVC) in a row, ST elevation consistent with ischemia.

5.3 Exclusion Criteria

1. History of or active immunodeficiency or immuno-suppression caused by acquired or congenital diseases or caused by treatments such as chronic administration (> 14 days) of systemic, i.e. parenteral or oral, corticosteroids (> 5 mg prednisone [or equivalent] per day), radiation or immune-modifying drugs.
2. Periodic steroid injections, e.g. intraarticular, are not allowed within 30 days prior to the first vaccination and throughout the study until Visit 5 (V5).
3. Post organ transplant subjects whether or not receiving chronic immunosuppressive therapy.
4. Uncontrolled serious infection, i.e. not responding to antimicrobial therapy.
5. History of any serious medical condition, which in the opinion of the investigator would compromise the safety of the subject or prevent the subject from complying with study requirements.
6. History of or active autoimmune disease, e.g. Type I diabetes. Persons with vitiligo or thyroid disease taking thyroid hormone replacement are not excluded.
7. Skin cancer in the past six months. If treatment for skin cancer was successfully completed more than six months ago and the malignancy is considered to be cured, the subject may be enrolled. Subjects with history of skin cancer must not be vaccinated at the previous site of cancer.
8. Any other malignancy in the past five years. If treatment for cancer was successfully completed more than 5 years ago and the malignancy is considered to be cured, the subject may be enrolled.
9. Clinically significant hematological, renal, hepatic, pulmonary, central nervous, cardiovascular or gastrointestinal disorders which are not adequately controlled by medical treatment within the last 12 weeks before vaccination as judged by the site's Principal Investigator.
10. History of myocardial infarction, congestive heart failure with marked limitation of activity due to symptoms, e.g. walking short distances [20-100 m] (i.e. > Grade II according to the New York Heart Association), cardiomyopathy and stroke or transient ischemic attack in the past two years.

11. Uncontrolled high blood pressure defined as systolic blood pressure ≥ 150 mm Hg and/or ≥ diastolic blood pressure ≥ 100 mm Hg within the last six months.
12. Subjects with active coronary heart disease manifested by angina, even if on medication.
13. 25 % or greater risk of developing a myocardial infarction or coronary death within the next 10 years using the National Cholesterol Education Program's Risk Assessment Tool: http://hin.nhlbi.nih.gov/atpiii/calculator.asp
14. Clinically significant mental disorder not adequately controlled by medical treatment.
15. History of chronic alcohol abuse (40 g/day, e.g. 3 glasses of beer or 2 glasses of wine for at least six months) and/or intravenous drug abuse (within the last six months). Subjects with a history of other substance and/or alcohol abuse are also excluded if – in the opinion of the investigator – the abuse could prevent the subject from complying with study requirements.
16. History of allergic disease or reactions likely to be exacerbated by IMVAMUNE® or any component of the vaccine, e.g. tris(hydroxymethyl)-amino methane, chicken embryo fibroblast proteins, aminoglycosides (gentamycin).
17. History of anaphylactic shock or any severe allergic reaction to a vaccine requiring immediate treatment.
18. Subjects undergoing treatment for tuberculosis infection or disease.
19. Having received any vaccinations or planned vaccinations with a live vaccine within 30 days prior to or after study vaccination.
20. Having received any vaccinations or planned vaccinations with a killed vaccine within 14 days prior to or after study vaccination.
21. Administration or planned administration of immuno-globulins and/or any blood products during a period starting from three months prior to administration of the vaccine and ending at study conclusion.
22. Use of any investigational or non-registered drug or vaccine other than the study vaccine within 30 days preceding the first dose of the study vaccine or planned administration of such a drug during the study period.
23. Temperature ≥ 100.4°F (38.0°C) at the time of enrollment.
24. Any condition which might interfere with study objectives or would limit the subject's ability to complete the study in the opinion of the investigator.
25. Study personnel.

6. Withdrawal of Subjects

6.1 Individual Withdrawal Criteria during the Study

Subjects may withdraw or be removed from the study for any of the reasons cited below:

- An AE occurs that, in the opinion of the investigator, makes it unsafe for the subject to continue the study. In this case, the appropriate measures will be taken.
- Subject's request to withdraw.
- Subject unwilling or unable to comply with study requirements.
- Clinical need for concomitant or ancillary therapy not permitted in the study.
- Unrelated intercurrent illness that, in the judgment of the investigator, will affect assessment of clinical status to a significant degree.

6.2 Contraindications and Precautions for further Study Vaccinations

The following criteria should be checked prior to second vaccination. If any are applicable, the subject should not receive the second vaccination, but should continue other study procedures:

- Anaphylactic reaction following the administration of any vaccine(s).
- Use of any investigational or non-registered drug or vaccine other than the study vaccine.
- Administration of a licensed vaccine not foreseen by the study protocol.
- Start of chronic administration (defined as more than 14 days) of > 5 mg prednisone (or equivalent) per day or any other immune-modifying drugs during a period starting from three months prior to administration of the vaccine and ending with the conclusion of active study phase (V5). Periodic steroid injections, e.g. intraarticular, are not allowed within 30 days prior to the first vaccination and throughout the study until Visit 5
- Administration of immunoglobulins and/or any blood products.
- A troponin I value at V2 ≥ 2x ULN.
- Pregnancy.

Temporary deferral of 2nd vaccination:
If an acute illness is present at the time scheduled for the 2nd vaccination, the subject may be vaccinated at a later date within the foreseen time window. The vaccine can be administered to persons with a minor illness such as diarrhea, mild upper respiratory infection, or any other mild condition with or without low-grade febrile illness, i.e. oral temperature ≤ 100.4°F (≤ 38.0°C).

The investigator may also decide not to administer the 2nd vaccination, if such a condition is present, in which case the subject should continue other procedures until conclusion of the study.

6.3 Subject Withdrawal Procedure

If a subject discontinues prior to completion of the study, the date and reason for the discontinuation will be obtained.

Once a subject has received at least one dose of IMVAMUNE®, he/she must be followed for safety as stated in the protocol. From the time of discontinuation, all diagnostic procedures and evaluations scheduled for V4 should be performed (see 1.7 Flow Chart).

As a general rule, subjects who discontinued the trial after having received at least one vaccination will not be replaced. Subjects that have been included in the study but already discontinued the trial prior to the first vaccination should be regarded as screening failures.

7. Study Treatment

7.1 Investigational Product

IMVAMUNE® is a highly attenuated live vaccinia Virus. It will be provided in liquid-frozen aliquots (Lot 0070808). One dose of 0.5 ml liquid-frozen vaccine contains 1 x 10^8 $TCID_{50}$ MVA-BN®. IMVAMUNE® will be given s.c.

For details see current version of the Investigator's Brochure.

Placebo consists of the IMVAMUNE® formulation buffer, Tris-buffered saline. It will be provided in liquid aliquots (Lot TBS-005-07-08). One dose of 0.5 ml placebo contains 1.21 mg/ml tris(hydroxymethyl)-amino methane and 8.18 mg/ml sodium chloride.

7.2 Packaging and Labeling

IMVAMUNE®:
The bulk drug substance MVA-BN® is produced at Bavarian Nordic A/S and the final drug product IMVAMUNE® is filled and labeled at the contract manufacturer IDT Biologika GmbH.

Addresses:

Bavarian Nordic A/S
Klaus B. Madsen (QA)
Hejreskovvej 10A
DK-3490 Kvistgård, Denmark
Phone: +45 3326 8383

IDT Biologika GmbH
Dr. Margrit Gehrt
Am Pharmapark
06861 Dessau-Rosslau, Germany
Phone: +49 34901 885 0

Placebo is produced and labeled at Bavarian Nordic GmbH in Berlin, Germany.

Address:
Bavarian Nordic GmbH
Uwe Werner
Robert-Roessle-Strasse 10
13125 Berlin, Germany
Phone: +49 30 9406 3900

IMVAMUNE® vaccine and placebo are provided separately in an openly labeled manner. The vaccine and the placebo will be shipped to the location/person at the clinical study site in charge of vaccine preparation, e.g. the pharmacy/pharmacist.

Packages and vials of vaccine and placebo will be labeled with the US IND label.

7.3 Storage and Handling

The liquid-frozen IMVAMUNE® vaccine has to be stored at -20 °C ± 5 °C / -4 °F ± 9 °F. A vial may not be re-frozen once it has been thawed. The placebo has to be stored at +2°C to +8 °C. / 36°F to 46°F.

Details on vaccine handling can be found in BN's SOP, SOP/CLIN/016, entitled "Storage, Handling and Vaccination Procedures of Liquid Frozen MVA-BN® (IMVAMUNE®) for Clinical Trials".
Handling of the placebo is in analogy to what is described for IMVAMUNE® after thawing.

7.4 Randomization

Subjects eligible for Groups 1 and 2 will be randomly allocated per center to one of the two treatment groups in order of their appearance in a 1:1 ratio.

The randomization list will be prepared by an independent Contract Research Organization (CRO). This CRO is not responsible for study management. At the investigational site, only study independent personnel and the unblinded person preparing the study medication will have access to the randomization list. In case of emergency which makes unblinding necessary, the Medical Monitor of BN (contact details listed in Section 1.3) will also have access to a sealed copy of the randomization list.

Members of the DSMB will be provided with blinded information in the first instance. Only if safety concerns are raised on the blinded data will unblinded information be provided to the DSMB. Should the need arise, the independent CRO will prepare any unblinded tables and listings requested by the DSMB. This unblinded information will not be given to members of BN (unless there is an urgent safety need). The DSMB will discuss the unblinded information in a closed DSMB session, meaning the PIs, BN and representatives of the CRO responsible for study management are not allowed to attend.

After the final database lock and after authorization from BN has been obtained, the CRO responsible for the randomization list will provide the list to the CRO responsible for data management of the study. From this point onward the study will be considered unblinded.

7.5 Preparation, Administration and Dosage

The preparation of the vaccine/placebo will be performed by a person, e.g. the pharmacist, who is the only unblinded person at the study site. This person must not be involved in the study treatment and/or the evaluation of study subjects.

For administration, the vaccine vial will be thawed at room temperature. To ensure homogeneity upon thawing, the vial will be swirled gently (not shaken) for at least 30 seconds. After thawing the drug product should appear as a pale milky colored suspension. The liquid vaccine should be visually inspected for any foreign particulate matter prior to administration. In case foreign particulate matter is visible, the vaccine must not be used. Vials of placebo will be removed from the refrigerator, gently swirled and inspected for any foreign particulate matter.

The injection volume of 0.5 ml vaccine / placebo will be withdrawn with a syringe using an injection needle long enough to reach the bottom of the vial (minimum length: 38 mm / 1½ inch). After withdrawal of the vaccine / placebo, the needle should be changed to a s.c. injection needle.

If the vaccine cannot be administered immediately, it is recommended to administer the product within 12 hours after thawing. During this time, the thawed vaccine vial has to be stored between 35.6 °F to 46.4 °F (+2 °C to +8 °C) in the dark, e.g. in a refrigerator.

The readily prepared syringe must be labeled with the study number, subject number and the date / time of preparation. The prepared syringe should not allow identification of the contents, i.e. whether the syringe contains IMVAMUNE® or placebo. The vaccine / placebo should be administered to the subject immediately (within 30 minutes) after delivery from the unblinded person, e.g. the pharmacist, to the vaccinator.

Subjects in study Group 1 will receive two s.c. vaccinations with 0.5 ml IMVAMUNE® vaccine four weeks apart (V1 / Day 0 and V3 / V1 + 28–35 days), each dose containing at least 1 x 10^8 $TCID_{50}$, preferably in the non-dominant upper arm (deltoid region).

Subjects in study Group 2 will receive the first s.c. vaccination with 0.5 ml Placebo, the second s.c. vaccination will consist of one IMVAMUNE® dose (1 x 10^8 $TCID_{50}$) four weeks later (V1 / Day 0 and V3 / V1 + 28-35 days), preferably in the non-dominant upper arm (deltoid region).

The rationale for the selected dose and the route of administration (see also Section 2.5.2) derives from preclinical and clinical data that have shown satisfactory immune responses after a 1 x 10^8 $TCID_{50}$ dose and a good safety profile of IMVAMUNE® when administered via the s.c. route.

Details on vaccine administration is provided in Bavarian Nordic’s SOP, SOP/CLIN/016, entitled “Storage, Handling and Vaccination Procedures of Liquid Frozen MVA-BN® (IMVAMUNE®) for Clinical Trials”.

7.6 Accountability and Disposal

Used and unused vials of IMVAMUNE® should be stored in a safe place and remain the property of BN. The Principal Investigator of the respective site or his designee is responsible for ensuring adequate accountability of all used and unused study drug. This includes acknowledging receipt of each shipment of study drug (quantity and condition) and maintenance of a drug inventory log. The drug inventory log will document quantity of IMVAMUNE® received from BN, quantity of study drug used for vaccination (including lot number, date dispensed, subject identification number and initials of the person dispensing the study medication) and quantity of study drug returned to BN or destroyed.

In case destruction is agreed, material should be discarded at the site according to local regulations. Additionally, used syringes should be autoclaved or incinerated and discarded at the site according to local regulations.

7.7 Concomitant Medication

All concomitant medications except homeopathic substances and dietary supplements must be recorded in the case report form (CRF) with the reason for administration, the dosage regimen, and the onset and end of treatment.

8. Clinical and Laboratory Assessments

8.1 General

8.1.1 Medical History

The baseline assessment for safety parameters will be performed during the screening visit (Visit SCR). History will focus particularly on any important diseases and in case of infections or tumors, the pathogen involved or the pathological diagnosis, respectively, if available. Special attention should be given to history of prior allergic reactions, especially to vaccines, as well as to history of alcohol and/or intravenous drug use.

8.1.2 Complete Physical Examination

The complete physical examination will be performed at the screening visit. The examination includes a review of major organ systems and determination of body weight. The examination should be directed at finding evidence of any infections, tumors and/or lymphadenopathy.

In addition, listening to the heart and lungs specifically for heart failure, presence of rubs, gallops, murmurs, crackles, and rales will be performed.
The examination must be performed by a person licensed to perform medical assessments such as physical exams.

8.1.3 Targeted Physical Examination

A targeted physical examination will be performed at every study visit (V1- FU). The subject is examined regarding any signs and symptoms previously identified or any new symptoms observed since the last visit.

In addition, listening to the heart and lungs specifically for heart failure, presence of rubs, gallops, murmurs, crackles, and rales will be performed.

The examination must be performed by a person licensed to perform medical assessments such as physical exams.

8.1.4 ECG and Cardiac Event Assessment

A standard 12-lead ECG will be taken on all subjects at the Visit SCR, V2 and V4.

ECGs will be evaluated by a centralized procedure. The workflow / communication flow will be provided in a separate manual.

Subjects who develop any kind of symptoms during the study that are considered by the PI to be possibly cardiac related, such as but not limited to chest pain, dyspnea, arrhythmia, or edema, are referred to a cardiologist for cardiac evaluation which may include measurement of cardiac enzymes, (treadmill) ECG and/or echocardiogram as deemed necessary by the cardiologist. Depending on the results of this evaluation, further diagnostic tests will be done as recommended by the cardiologist and subjects will be followed up as determined by the cardiologist.

Furthermore, unclear ECG abnormalities observed during the study will be examined upon recommendation of the cardiologist.

Newly developing clinically significant cardiac AEs fulfill the definition of an “AE of special interest” and are to be handled and reported as described under Sections 9.1.3 and 9.3.

Using replication-competent vaccinia-based smallpox vaccines during smallpox vaccination programs in the US during the last years, cases of acute myocarditis and pericarditis were observed (Grabenstein & Winkenwerder, Jr. 2003).

Case definitions as published by the Centers of Disease Control and Prevention ("Update: Cardiac-Related Events During the Civilian Smallpox Vaccination Program -- United States, MMWR May 30, 2003, Vol. 52, No. 21, p. 494") are provided in Appendix II in order to:

- help investigators to recognize possible events of acute myocarditis and/or pericarditis and
- distinguish from unspecific and isolated ECG changes without or with unclear clinical meaning.

Figure 3 outlines the algorithm for assessment of cardiac events.

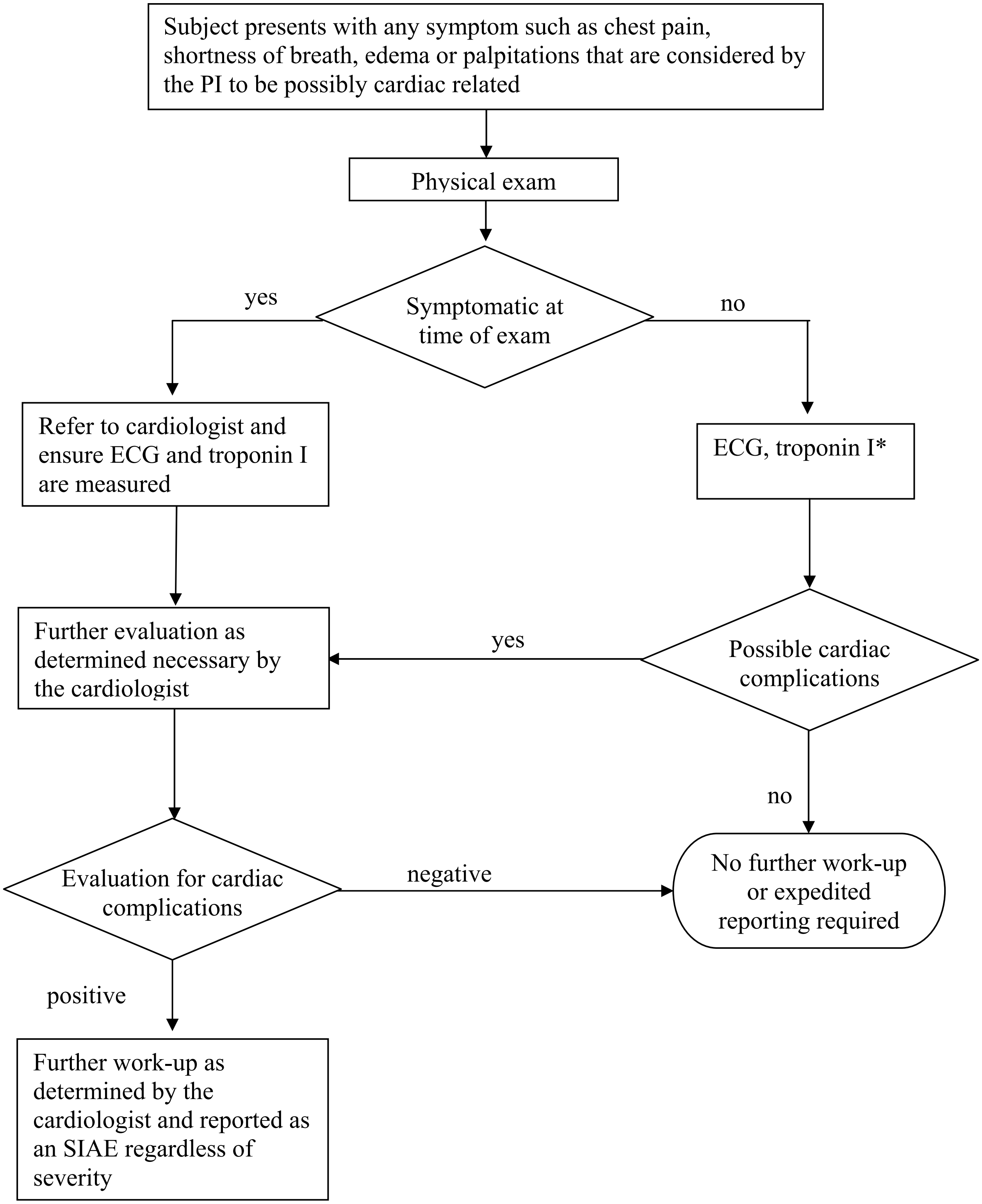

Figure 3: Algorithm for Assessment of Cardiac Events
*At any protocol-scheduled ECG and/or troponin I abnormality, the algorithm will begin at this point.

8.2 Assessment of Safety and Reactogenicity

8.2.1 Assessment of General Safety Parameters

8.2.1.1 Vital signs

Blood pressure, heart rate and temperature will be taken during screening (Visit SCR), during the treatment period at each visit (V1–5) and at study end (Visit FU). Body weight will be measured at Visit SCR.

8.2.1.2 Laboratory Measurements

The severity of laboratory / systemic quantitatively measured toxicities will be graded according to the toxicity scale in Appendix I. These grading scales include the laboratory values determined with the routine safety parameters. In case of other laboratory values not included in the routine safety laboratory and not listed in Appendix I, the National Cancer Institute Common Toxicity Criteria table, Version 2.0, published April 30, 1999 will be used for grading of laboratory toxicities.

8.2.1.3 Safety Laboratory

Safety laboratory is determined at Visit SCR, V2 and V4. The safety laboratory measurements are performed at a central laboratory. Laboratory normal ranges are provided by the central laboratory and filed in the investigator file. Safety laboratory parameters to be evaluated are:

Hematology
Red blood cell count, hemoglobin, total and differential WBC, platelet count

Serum chemistry
Total bilirubin, alkaline phosphatase, AST, ALT, serum creatinine, sodium, potassium, calcium, troponin I

Pregnancy test
β-Human choriogonadotropin (HCG) pregnancy tests will be conducted for all women at screening (Visit SCR), within 24 hours prior to vaccination and 28–35 days after the second vaccination (V5). At screening a serum β-HCG pregnancy test will be conducted and all other pregnancy tests may be conducted as urine β-HCG tests. Results of pregnancy tests must be known prior to each vaccination.

The following parameters will only be evaluated during the screening period (Visit SCR) to check for compliance with inclusion / exclusion criteria:

Cholesterol (total, High Density and Low Density Lipoprotein)
Urine analysis (e.g. with Combur-10 stick)
Protein, glucose, occult blood, nitrites, leucocytes, pH, bilirubin, ketones, urobilinogen, specific gravity

8.2.2 Assessment of AEs

Details regarding the definitions and reporting are described in Section 9 of this protocol.

8.2.2.1 Assessment of Solicited Local AEs

After the vaccination each subject receives a diary to record solicited local and general AEs most likely to occur on the day of vaccination or the following 7 days. All solicited AEs observed after vaccination with details concerning the severity and the course of the event should be documented there. The investigator will collect this information during the next scheduled visit and will assess the relationship of the solicited general events to study medication.

In case of severe and unexpected local and systemic reactions, the study physician should be contacted outside from scheduled study visits.

To standardize procedures uniform rulers will be handed out to all subjects for measurements of erythema, swelling and induration diameters and digital thermometers for oral measurements of body temperature.

Solicited Local AEs
The solicited local symptoms erythema, swelling, induration, pain and pruritus at the injection site have to be documented in the diary by the subject and severity is assessed in the following way:

Injection site erythema	size measured in diameter
Injection site swelling	size measured in diameter
Injection site induration	size measured in diameter

The maximum severity will be scored as follows:

0	=	0
1	=	< 30 mm
2	=	≥ 30 – <100 mm
3	=	≥ 100 mm

Injection site pruritus:

0	=	Absent
1	=	Mild
2	=	Moderate
3	=	Severe

Injection site pain:

0	=	Absent
1	=	Painful on touch
2	=	Painful when limb is moved
3	=	Spontaneously painful / prevents normal activity

8.2.2.2 Assessment of Solicited Systemic AEs

Subjects are asked to document the solicited general AEs as described in the table below on the day of vaccination and the following 7 days in their diary card. Symptoms continuing beyond the 7 days captured in the diary card should be documented daily until resolution or until the next study visit.

In the subject's diary the grading of maximum symptom severity is described in basic, easily understood language based on the following descriptions.

Table 6: Grading of Symptoms from the Subject's Diary

MedDRA coded Preferred Term<br>General AEs	Grade	Maximum Severity
Body temperature*	0	< 99.5 °F (< 37.5 °C)
	1	≥ 99.5 – <100.4 °F (≥ 37.5 – < 38.0 °C)
	2	≥ 100.4 – 102.2 °F (≥ 38.0 – < 39.0 °C)
	3	≥ 102.2 – 104 °F (≥ 39.0 – < 40.0 °C)
	4	≥ 104°F (40.0 °C)
Headache, Myalgia, Nausea and Fatigue	0	None
	1	Mild: easily tolerated, minimal discomfort and no interference with daily activity
	2	Moderate: Some interference with daily activity
	3	Severe: Prevents daily activity

*Pyrexia is defined as oral temperature ≥ 100.4 °F (≥38.0 °C).

Causal relationship between solicited general AEs and the study vaccine will be assessed by the investigator.

8.2.2.3 Assessment of Unsolicited AEs

During every study visit following screening, the investigator has to record any unsolicited AE experienced by the subject.

Unsolicited AEs following the vaccination will generally be recorded by the subjects in a special section of the diary card and transferred by the investigator to the AE section of the CRF. In addition, all intercurrent diseases not recorded on the diary card but reported when the investigator actively inquires of the subject will be documented in the respective section of the CRF.

AEs will be assessed and documented at all visits of the active study phase and if ongoing at Visit 5, followed until resolution or the FU visit at the latest.

SAEs will be assessed and documented at all study visits, including the FU Visit. Ongoing SAEs will be followed up until resolution or achievement of stable clinical conditions.

8.2.2.4 Assessment of Severity for AEs

The scale for grading the maximum severity of all AEs will be based on the following descriptions:

Grade 1 An AE which is easily tolerated by the subject, causing minimal discomfort and not interfering with daily activities.

Grade 2 An AE which is sufficiently discomforting to interfere with daily activities.

Grade 3 An AE which prevents daily activities. Such an AE would, for example, prevent attendance at work and would necessitate the administration of corrective therapy.

Grade 4 Life-threatening or disabling

8.2.2.5 Assessment of Laboratory Measured Toxicities

The severity of laboratory toxicities will be graded according to the toxicity scale in Appendix I. This grading scale includes all laboratory values determined with the routine safety parameters as described in Section 8.2.1. In case of other laboratory values not included in the routine safety laboratory and not listed in Appendix I. the National Cancer Institute Common Toxicity Criteria table, Version 2.0, published April 30, 1999 will be used for grading of laboratory toxicities.

For more details on the procedure for reporting and documenting AEs refer to Section 9 of this protocol.

8.2.2.6 Causality Assessment

The relationship between the occurrence of an AE and the study drug will be assessed using the following categories:

None	• The time interval between the administration of the study drug and the occurrence or worsening of the AE rules out a relationship, **and/or**<br>• another cause is established and there is no evidence of a (concomitant) causal connection with or worsening caused by the study medication.
Unlikely	• The time interval between administration of the study drug and the occurrence or worsening of the AE makes a causal relationship unlikely, **and/or**<br>• the known effects of the study medication or substance class provide no indication of a (concomitant) causal connection with or worsening caused by the study medication and there is another cause which serves as an adequate explanation, **and/or**<br>• although the known effects of the study medication or substance class make it possible to derive a plausible causal chain with regard to a (concomitant) causal connection or worsening, however, another cause is considerably more likely, **and/or**<br>• another cause of the AE has been identified and a (concomitant) causal connection with or worsening caused by the study medication is unlikely.
Possible	• A plausible causal chain with regard to a (concomitant) causal connection with / worsening of the AE can be derived from the pharmacological properties of the study medication or substance class. However, other approximately equally likely causes are known, **or**<br>• although the pharmacological properties of the study medication or substance class provide no indication of a (concomitant) causal connection with / worsening of the AE, there is no other known cause which provides an adequate explanation.
Probable	• The pharmacological properties of the study medication or substance class, **and/or**<br>• the course of the AE after discontinuation of the study drug and possible subsequent re-exposure, **and/or**<br>• specific findings (e.g. positive allergy test or antibodies against the trial drug / metabolites) suggest a (concomitant) causal connection with / worsening of the AE resulting from the study medication, however another cause cannot completely be ruled out.
Definite	• The pharmacological properties of the study medication or substance class **and/or**<br>• the course of the AE after discontinuation of the study drug and possible subsequent re-exposure, **and/or**<br>• specific findings (e.g. positive allergy test or antibodies against the trial drug / metabolites) definitely indicate that there is a (concomitant) causal connection with / worsening of the AE resulting from the study medication and there are no indications of other causes.

8.3 Assessment of Immunogenicity

The method of collection, storage and handling of laboratory specimen for the immune analysis is specified in SOP/CA/020: “Instructions for the Collection, Preparation and Storage of Serum Samples”. A written instruction for this procedure will be provided to the investigators before enrollment. Additionally, the procedure will be explained in detail during the investigator meeting and/or at the site initiation visit.
Only specimen testing outlined in this protocol will be conducted with biologic specimens collected during this study. Residual specimens may be stored long-term at the sponsor, with the subject’s consent.

8.3.1 Antibody Response

Immune response analysis is planned at all study visits except the screening visit. The baseline assessment for immunogenicity parameters will be performed during V1 (before vaccination).

Antibody responses against IMVAMUNE® will be measured using a direct vaccinia-specific ELISA and a vaccinia-specific PRNT, both established and validated in-house assays.

Immune analyses will be performed at BN’s laboratory at Bavarian Nordic GmbH, 82152 Martinsried, Germany. The protocols for the analytical tests performed are detailed in the following SOPs. The latest versions of the SOPs will be filed in the Trial Master File:

SOP/CA/017: “Plaque Reduction Neutralization Assay Using Vaccinia Virus Western Reserve”.

SOP/CA/029: “(Automated) Standard ELISA for Detection of Vaccinia Specific Antibodies in Human Sera”.

The names and titles of the mentioned SOPs can be subject to changes or updates during the trial.

8.3.1.1 ELISA

Immune response will be assessed as appearance of antibody titers ≥ 50 in the vaccinia-specific ELISA for initially seronegative subjects, or an increase of the antibody titer compared to the baseline (V1) titer for subjects with a pre-existing antibody titer in the ELISA.

The GMT is calculated by taking the antilogarithm of the mean of the log10 transformations. Antibody titers below the cut-off of the assay will be given an arbitrary value of one for the purpose of this calculation.

8.3.1.2 PRNT

Immune response will be assessed as appearance of antibody titers ≥ 6 in a vaccinia-specific PRNT assay for initially seronegative subjects, or an increase of the antibody titer compared to the baseline titer (V1) for subjects with a pre-existing antibody titer in the PRNT.

The GMT is calculated by taking the antilogarithm of the mean of the log10 transformations. Antibody titers below the cut-off of the assay will be given an arbitrary value of one for the purpose of calculation.

9. Safety Assessment and Reporting

Any signs and symptoms that occur before first vaccination will be recorded in the baseline signs and symptoms section and will not be considered as AEs.

9.1 Definition of AE

AEs are defined as any untoward (undesirable) medical occurrence in a clinical trial subject administered a medicinal product and which does not necessarily have a causal relationship with this medication. All AEs (e.g. feeling of ill-health, subjective symptoms and objective signs, intercurrent diseases, accidents, etc.) observed by the investigator and/or reported by the subject must be recorded in the CRF regardless of the assessment of causality in relationship with the study drug.

Abnormal laboratory values that were assessed as clinically significant by the investigator are to be documented as AEs. In addition, abnormal laboratory values fulfilling the Grade 3 criterion according to the toxicity scale (Appendix I) are to be documented as AEs, regardless of whether they are considered clinically relevant or not.

9.1.1 Definition of Solicited AE

Within this study protocol solicited AEs are defined as all events recorded by the subjects in the diary provided to them following every vaccination. For each of the 7 days post-vaccination the subjects are requested to monitor and record local symptoms, i.e. erythema, swelling, induration, pruritus and pain at the site of injection as well as general symptoms, i.e. body temperature, headache, myalgia, nausea and fatigue.

9.1.2 Definition of Unsolicited AE

At every study visit the investigator should ask the subject if they have experienced any AEs since their last visit. All intercurrent diseases reported by the subject, regardless of whether recorded in the subject diary or not, need to be recorded by the investigator in the appropriate page of the CRF.

The severity and causality of the events will be graded according to the procedures described in Section 8.2.2 Assessment of AEs.

9.1.3 Definition of "AE of Special Interest" (SIAE)

An “AE of special interest” or “special interest AE” (SIAE) is defined in this study as:

- Any cardiac sign or symptom developing during the study which is deemed to be clinically significant by the Investigator (defined in Section 8.1.4)
- ECG changes determined to be clinically significant
- Cardiac enzymes elevated above 2 x ULN

SIAEs are to be reported according to the procedures and timelines applicable for SAEs (see Section 9.3).

9.2 Definition of SAE

An SAE is any untoward medical occurrence or effect that at any dose:

- Results in death,
- Is life-threatening*,
- Requires inpatient hospitalization or prolongation of existing hospitalization,
- Results in persistent or significant disability or incapacity,
- Is a congenital anomaly or birth defect,
- or is an otherwise important medical event.

* The term "life-threatening" in the definition of "serious" refers to an event in which the patient was at risk of death at the time of the event; it does not refer to an event which hypothetically might have caused death if it were more severe.

Medical and scientific judgment should be exercised in deciding whether expedited reporting is appropriate in other situations, such as important medical events that may not be immediately life-threatening or result in death or hospitalization but may jeopardize the patient or may require intervention to prevent one of the other outcomes listed in the definition above. These should also usually be considered serious.

Examples of such events are intensive treatment in an emergency room or at home for allergic bronchospasm; blood dyscrasias or convulsions that do not result in hospitalization; or development of drug dependency or drug abuse.

Hospitalization for performing an elective surgery which is unrelated to the vaccine or a study procedure is not considered an SAE, rather should be documented as an AE.

Timelines
AEs will be followed up and documented up to 28 days after vaccination. SAEs will be followed up through the course of the study and the FU-phase. Ongoing AEs will be followed up until resolution or end of the study. Ongoing SAEs will be followed up until resolution or stable clinical conditions.

Severity of AEs
The severity of the event will be graded according to the procedure described under Section 8.2.2

9.3 Reporting of SAEs

All SAEs or SIAEs occurring throughout the entire course of the study have to be reported to the Kendle Safety Department. The study site has to send by e-mail or fax the completed SAE form to the Kendle Safety Department within 24 hours of becoming aware of the SAE or SIAE.

Kendle Safety forwards all serious adverse events and adverse events of special interest within 24 hours to Bavarian Nordic Drug Safety. Bavarian Nordic is responsible for expedited reporting to the involved regulatory authorities (e.g. FDA) and to the concerned investigators and IECs/IRBs in compliance with national and international regulations as well as to the responsible NIH-DMID representatives. In addition each PI must inform their local IRB in accordance with their IRB rules and regulations.

Figure 4 outlines the reporting process and timelines.

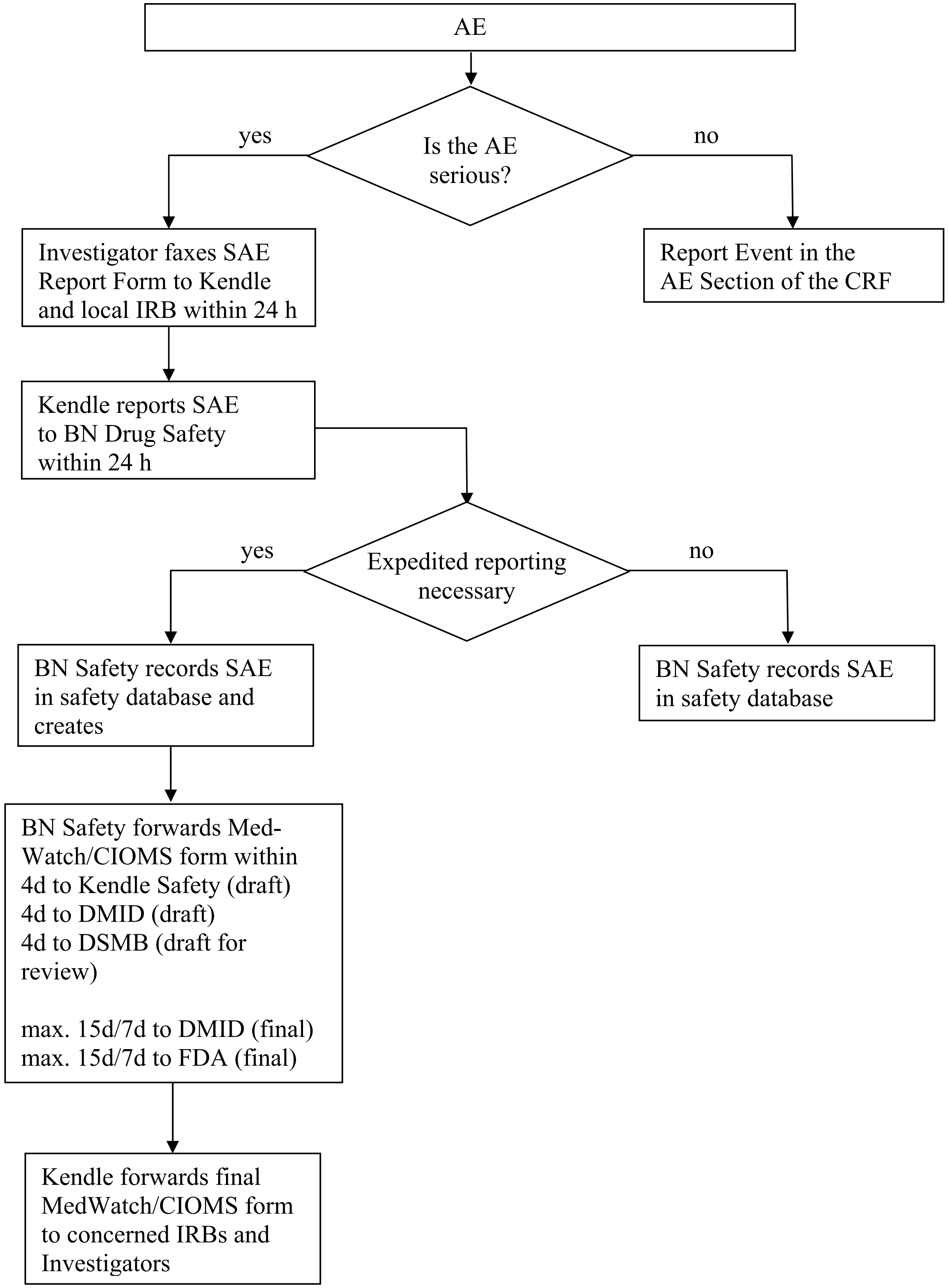

Figure 4: Algorithm for Reporting of SAEs

SAE / SIAE reports should be faxed to the following number:

Kendle Safety:

fax: +1 800 352 8133

phone: +1 800 265 1542

The investigator should not delay reporting because of missing information. Nonetheless, the report should be as complete as possible. This initial notification should include, as a minimum, sufficient information to permit identification of the following:

- the reporter (investigator's name and contact information)
- the subject
- involved study medication
- AE(s)
- date of onset

9.4 Pregnancy

Subjects who become pregnant during the study period (up to and including one month [minimum 28 days] after receiving a dose of vaccine) must not receive additional doses of vaccine but may continue other study procedures at the discretion of the investigator.

Subjects should be instructed to notify the investigator if it is determined after completion of the study that they became pregnant either during the study or within one month (minimum 28 days) after receiving the last vaccine dose. Pregnancy must be reported to BN on a Pregnancy Form within 24 hours of the investigator's becoming aware of the event.

A pregnancy should be followed to term, any premature terminations reported, and the health status of the mother and child including date of delivery and the child's gender and weight should be reported to BN after delivery.

10. Statistical Considerations

The primary objective is to expand the IMVAMUNE® data base on safety in a vaccinia-experienced population 56-80 years of age after administration of either one or two doses of IMVAMUNE®.

The secondary objectives are

- to investigate the safety and reactogenicity of IMVAMUNE® in a vaccinia-experienced population 56-80 years of age after administration of either one or two doses of IMVAMUNE®.
- to compare the immunogenicity of IMVAMUNE® in a vaccinia-experienced population 56-80 years of age after administration of either one or two doses of IMVAMUNE®.

Statistical methods will only be applied in an explorative manner.

Data of subjects discontinuing the trial will be used to their maximal possible extent.

10.1 Statistical Analysis Plan

The statistical methods for the analyses will be discussed in detail in the Statistical Analysis Plan (SAP). The planned statistical analysis may change if circumstances should arise during the study rendering this analysis inappropriate, or if in the meantime improved methods of analysis should come to light. Any changes to the planned statistical analysis will be described in the clinical study report.

The SAP will be finalized before the database of the study is locked and unblinded. SAS software® (Version 8.2 or higher) will be used for all data presentation and summarization including statistical analyses, summary tables, graphs and data listings.

10.2 Sample Size Calculations

A total of 120 healthy subjects will be enrolled in 2 groups of 60 subjects each.

The sample size calculation is based on the primary endpoint to evaluate the incidence of serious adverse reactions. A sample size of 60 subjects will provide a probability of 95% to detect serious adverse drug reactions with an incidence of at least 5%, i.e. 60 patients guarantee a 95% certainty of detecting at least one serious adverse drug reaction that occurs with an incidence of 1 in 20.

The following is a supportive calculation to show that this study should also have sufficient power to test if one dose of IMVAMUNE® produces a significantly lower response to using two vaccinations with IMVAMUNE®.

From the vaccinia-experienced group (N=200) in the POX-MVA-005 study (where a single vaccination of IMVAMUNE® of 1 x 10^8 $TCID_{50}$ was given to healthy subjects) an ELISA seroconversion rate of 95.3% was observed. This seroconversion rate is higher than the 87.4% seroconversion rate observed in the vaccinia-naïve group who were given a single vaccination of IMVAMUNE® at 1 x 10^8 $TCID_{50.}$ However, after two vaccinations with IMVAMUNE® the seroconversion rate observed in the vaccinia-naïve subjects rose to 98.9%. It is therefore not unreasonable to assume that the peak seroconversion rate in Group 1 in this study will be at least 98.9% after two vaccinations in vaccinia-experienced subjects. Assuming a peak response rate of 98.9% in Group 1 and 95.3% in Group 2, with a power of 80% and a significance level of 95% the required sample size to demonstrate a difference in response rates between the two groups is then N=52 per group in the PPS.

10.3 Endpoints

10.3.1 Safety and Reactogenicity

Primary endpoint:
Occurrence of any SAEs associated with the study vaccine occurring until the last active study visit (Visit 5).

Secondary endpoints:
Occurrence of unsolicited non-serious AEs within 28 days after each vaccination: Severity, duration and relationship to vaccination.

Occurrence of any Grade 3 or 4 AEs associated with the study vaccine within 28 days after each vaccination.

Occurrence, relationship and severity of any cardiac events and/or any ECG change indicating a case of myo-/pericarditis at any time during the study.

Occurrence of solicited local adverse reactions within one week (Days 0-7) after each vaccination: Analysis of severity and duration.

Occurrence of solicited general AEs within one week (Days 0-7) after each vaccination: Analysis of severity, duration, and relationship to vaccination.

10.3.2 Immunogenicity

10.3.2.1 ELISA

Secondary endpoints:
Percentage of subjects with responses, i.e. either an appearance of antibody titers ≥ 50 in a vaccinia-specific ELISA for initially seronegative subjects or an increase of the antibody titer compared to the baseline titer for subjects with a pre-existing antibody titer, at the individual peak response.

Percentage of subjects with responses at all individual blood sampling time-points.

GMTs measured by vaccinia-specific ELISA titers at the individual peak response.
GMTs measured by vaccinia-specific ELISA titers at all blood sampling time points.
The GMT is calculated by taking the antilogarithm of the mean of the log10 titer transformations. Antibody titers below the cut-off of the assay will be given an arbitrary value of one for the purpose of this calculation.

10.3.2.2 PRNT

Percentage of subjects with responses, i.e. either an appearance of antibody titers ≥ 6 in a vaccinia-specific PRNT for initially seronegative subjects or an increase of the antibody titer compared to the baseline titer for subjects with a pre-existing antibody titer in the PRNT, at the individual peak response.

Percentage of subjects with responses at all individual blood sampling time-points.

GMTs measured by vaccinia-specific PRNT titers at the individual peak response.
GMTs measured by vaccinia-specific PRNT titers at all blood sampling time points.
The GMT is calculated by taking the antilogarithm of the mean of the log10 titer transformations. Antibody titers below the cut-off of the assay will be given an arbitrary value of one for the purpose of this calculation.

Pearson's correlation coefficient between antibody titers measured by ELISA and PRNT at each individual visit, and individual peak values. It is assumed that the data are log-normally distributed. Therefore, the titers will be log10 transformed for the correlation analysis.

10.4 Study Cohorts/Data Sets to be evaluated

For the statistical analysis the included subjects will be divided up into the following datasets:

Safety population:
This is the subset of subjects who were included and received at least one vaccination with either IMVAMUNE® or placebo and for whom any safety data are available.

The main analysis of safety will be performed on this population.

Full Analysis Set (FAS):
This is a subset of subjects in the Safety population and for whom baseline and any post vaccination immunogenicity data are available.

Per Protocol Set (PPS):
This is the subset of subjects in the FAS who have received both vaccinations and completed all study visits according to the protocol. Subjects with minor (not relevant) protocol violators can be included into this dataset.

The decision whether a protocol deviation is relevant or not for the classification of subjects into the various subsets will be made on a case-by-case basis in a blinded data review meeting prior to database lock.

The primary immunogenicity population dataset will be the FAS. For further descriptive purposes, the same statistical procedures will be applied to the PPS.

Analysis of immunogenicity variables will be done on a valid case basis, i.e. for missing observations no imputation technique such as "Last observation carried forward" (LOCF) will be applied, since this could introduce an optimistic bias into the analysis.

The Safety population will be used for the analysis of all safety data. In case the Safety population and FAS are the same then only the FAS will be reported.

10.5 Biometrical Evaluation

10.5.1 Analysis of Demographics and Baseline Characteristics

Descriptive statistics for the demographic data will be produced by treatment group and by age strata (56-70 and 71-80 years of age).

A Wilcoxon test for differences of the main baseline characteristics (age, body weight, height) will be performed by treatment group (2 groups).

10.5.2 Analysis of Immunogenicity

Antibody titers will be assessed by direct ELISA and PRNT method as described in Section 8.3.

Descriptive statistics for ELISA titers (N, GMT, Geometric Standard Deviation, 95% confidence interval for the GMT, median, minimum and maximum) will be calculated for each group at each visit and for the individual peak titers. The confidence intervals for the GMTs will be based on the assumption that the log10 transformations of the titers have a Normal distribution. Titers below the value of 50 will be assigned the arbitrary value of 1 for the purposes of these calculations. These descriptive statistics will also be presented by group and age stratum.

For descriptive purposes the p-values of differences between the groups will be calculated using the t-test on the log10 titers at each visit and for the individual peak values. The p-values will also be presented by group and age stratum.

Mean courses of the ELISA titer will be graphically displayed by group over the entire study course using the geometric means and 1 geometric standard deviation range. Titers will be displayed on the y-axis using a power of 10 scale, i.e. with tick marks 1, 10, 100, etc., while the visits will be displayed on the x-axis by target week of the visit.

Similar descriptive statistics will also be calculated for the PRNT GMTs (titers below the value 6 will be assigned the arbitrary value of 1 for the purposes of these calculations).

The main immunogenicity variable is the vaccinia-specific peak titer rate of response (either the appearance of a measureable titer in subjects who were initially seronegative, or an increase of the peak titer in initially seropositive subjects). This parameter is derived from the ELISA specific antibody titers.

The main immunogenicity hypothesis is to test whether the ELISA humoral immune response of subjects within each group are significantly different. That is, to investigate if the percentage of subjects that can be re-activated by a double dose of IMVAMUNE® is significantly above those seen using a single vaccination of IMVAMUNE®. This hypothesis will be tested by calculating an Exact 95% confidence interval for the difference in the rates (Group 1 - Group 2).

The rates of ELISA response for each visit (and individual peak titers) will be presented as percentages for each group, along with the Exact 95%, and differences between the groups (Group 1 - Group 2) along with corresponding 95% confidence intervals.

A similar stratified analysis will also be produced by group and age stratum.

Analogous tables should also be produced for the PRNT response rates (either the appearance of a measureable titer in subjects who were initially seronegative, or a response of the peak titer in initially seropositive subjects).

Scatter plots of ELISA titers vs. PRNT titers will be produced (with both titers plotted on the power of 10 scales) for each visit and for the individual peak titers. The best fit least-squares linear regression line will also be superimposed on each scatter plot. The Pearson's correlation coefficient will also be calculated for each visit (and peak) along with the associated 95% confidence interval for the correlation coefficient.

10.5.3 Analysis of Safety and Reactogenicity

10.5.3.1 Solicited Local AEs

The occurrence of solicited local AEs, i.e. injection site reactions listed in the subject diary, within 1 week after vaccination will be summarized on a per subject and per vaccination basis.

The maximum severity over the 7-day period after the vaccination day will be used and categorized as follows:

Pruritus / Pain:
- Any AE (i.e. Grade > 0)
- Grade < 2 / ≥ 2
- Severe AE (i.e. Grade = 3)

For measurements of diameter size:
- Any AE (i.e. Diameter > 0)
- Diameter < 30mm / ≥ 30 mm
- Severe AEs (i.e. Diameter ≥ 100 mm)

These categories will be compared between treatment groups by means of Fisher’s exact test. Note that all local solicited AEs are automatically considered as causally related to the study vaccine.

The duration of the AEs will also be summarized and compared between groups using the Wilcoxon test.

10.5.3.2 Solicited General AEs

Occurrence of solicited general AEs within 1 week after vaccination will be summarized per subject and per vaccination basis.

The maximum severity over the 7-day period after the vaccination day will be used and categorized as follows:

- Any AE (i.e. Grade > 0)
- Grade < 2 / ≥ 2
- Severe AEs (i.e. Grade ≥ 3)
- Causally related AEs (i.e. possibly, probably or definitely related to study vaccine)
- Causally related severe AEs (Grade ≥ 3)

These categories will be compared between treatment groups by means of Fisher's exact test.

The duration of the AEs will also be summarized and compared between groups using the Wilcoxon test.

10.5.3.3 Unsolicited AEs:

Unsolicited AEs will be coded with the MedDRA coding terminology. The severity of AEs will be graded according to Section 8.2.2.4.

The number of AEs and number of subjects with at least one AE for each preferred term will be descriptively compared between treatment groups in the 28 days following each vaccination. Similarly the number of severe (Grade 3 or 4) unsolicited AEs will be compared in the 28 days following each vaccinations, as well as the number of causally related unsolicited AEs, and the number of causally related severe unsolicited AEs.

All unsolicited AEs occurring outside of the 28 day window following each vaccination will be listed.

10.5.3.4 SAEs

SAEs and SIAEs will be listed separately. Each SAE and SIAE will be described individually in detail. The number of subjects with at least one SAE will be compared between treatment groups by means of the Fisher's exact.

10.5.3.5 Safety Laboratory

The individual laboratory findings will be listed per visit and summarized descriptively. Individual values will be evaluated using the laboratory normal ranges. The abnormal values will be flagged with "L" for values below the lower limit of the laboratory normal range and "H" for values above the upper limit of the laboratory normal range.

The intensity of the individual laboratory findings will also be graded according to the toxicity scale in Appendix I, which will be included in the listing and described per visit by frequencies and percentages for each laboratory parameter.

In case of any repeats of laboratory values, the repeated values will be used for the before-treatment assessment and the original value for the post-study assessment. All other repeated values will be listed in a separate listing.

10.5.3.6 Vital Signs and Physical Examinations

The individual values will be listed per time point and summarized descriptively. Physical examination data will be listed per time point.

10.5.3.7 Concomitant Medication

Concomitant medication will be coded using the WHODRUG dictionary (2008) and will be classified by Anatomical-Therapeutic-Chemical classification (ATC) categories and preferred term. Concomitant medication will be listed in full in an appendix.

10.5.4 Data Handling

All data obtained in this study and documented in the Electronic CRFs will be listed. For parameters of interest, summary tables with descriptive group statistics (mean, standard deviation, minimum, maximum, number of valid cases) for continuous variables will be prepared. For ordinal / dichotomous variables summary tables showing the absolute and percent in each category will be prepared.

11. Ethical Aspects

11.1 Ethical and Legal Regulations

Investigators are obliged to ensure that this clinical trial is conducted in complete accordance with the provisions of the Declaration of Helsinki (and its amendments of Seoul, Tokyo, Venice, Hong Kong, Somerset West and Edinburgh), the national laws and other guidelines for the conduct of clinical studies like the ICH Harmonized Tripartite GCP to guarantee the greatest possible subject protection.

11.2 Approval of an IEC/IRB and Institutional Biosafety Committee (IBC)

The protocol must be reviewed by the competent IEC/IRB and IBC (if applicable) according to the national and local laws of the respective site before the first subject is included in this study.

If one of the investigators is a member of one of these committees, he may not vote on any aspect of the review of this protocol.

The sponsor will assure that the IEC/IRB and IBC (if applicable) is informed of any amendment to the protocol, any unanticipated problems involving risks to human subjects included in the study and any SAE, if applicable. Such information will be provided to the committee at intervals appropriate to the degree of subject risk involved, but not less than once a year. Copies of all correspondence between the investigator and the committee must be forwarded immediately to the sponsor. In case of withdrawal of ethics committee approval of the study, the sponsor has to be contacted immediately by facsimile or telephone.

11.3 Confidentiality and Data Protection

The (Principal) Investigator of the respective site is obliged to ensure anonymity of the subject. He/she has to make sure that all documents including CRFs provided (e.g. in the course of a marketing authorization procedure) to third parties (in this case: to the manufacturer of IMVAMUNE® or to an authority) contain no subject names.

Subjects are only to be identified by a subject and center number, not by their name and/or clinic and subject file number. The (Principal) Investigator keeps separate confidential subject logs for study enrollment, which allow subject numbers to be matched with names and addresses of subjects at any time. Documents not meant to be passed on to third parties have to be stored confidentially by the (Principal) Investigator.

Any information collected in the course of the study may be made available only to persons directly involved in this study (Principal Investigator / Investigator, designated staff members, monitor, statistician) or to authorized persons by the sponsor or authorities / ethics committees.

12. Informed Consent

No subject is allowed to participate in this study without having signed an informed consent after the investigator or his delegate has informed the subject clearly and completely, verbally and in writing, over the purpose, procedures, potential benefits and risks of the current study and prior to study drug administration.

One signed copy of the informed consent must be given to each subject and one signed original must remain in the study documentation file and be available for verification by the monitor or competent regulatory authorities at any time.

Subjects must be informed unequivocally that they may refuse participation in the study and that they may withdraw from the study at any time and for whatever reason and that withdrawal of consent will not affect their subsequent medical treatment or relationship with the treating physician.

Subjects also consent to authorize the monitor, quality assurance personnel and regulatory authorities to inspect source documents for quality assurance purposes. Such verifications will always be conducted on site and under the ethical supervision of the investigator. All aspects of the confidentiality of the subject's data will be guaranteed.

The informed consent template must be submitted to the IEC/IRB. The written consent template will embody the elements of informed consent as described in the Declaration of Helsinki and will adhere to the ICH Harmonized Tripartite Guideline for GCP.

13. CRFs and Retention of Records

13.1 Electronic CRFs

All of the clinical data will be captured via electronic data capture (EDC) using a web-based tool. The investigator site staff will enter and edit the data via a secure network with secure access features (username and password). A complete electronic audit trail will be maintained. The investigator will approve the data using an electronic signature (Ref: 21 CFR Part 11), and this approval is used to confirm the accuracy of the data recorded.

Electronic CRFs will be used for all subjects. The investigator's data will be accessible from the investigator's site throughout the trial. The electronic CRFs must be kept current to reflect subject status at each phase during the course of the trial. The subjects are identified on the electronic CRF by number, gender and date of birth. The investigator must make a separate confidential record of these details (subject identification and enrollment log). While the trial is ongoing and until the access to the database has been terminated, there will be a full audit trail of changes. All changes to data are done by the investigator through the EDC system. If a change is necessary once the investigator has no further access to the database, a request for change will be sent to the investigator for confirmation of the change.

It is the responsibility of the (Principal) Investigator of the respective site to ensure that all subject discontinuations or changes in study or other medications entered on the subject's CRF are also made on the subject's medical records.

General comment: If the subject agrees, every effort should be made to contact the primary care physician to obtain the most recent history to describe the current medical status of the subject.

The CRF for any subject leaving the study should be completed at the time of the final visit or shortly thereafter.

13.2 Retention of records

The (Principal) Investigator shall maintain the records of disposition of drug receipts and drug inventory logs, subject files and regulatory documents (informed consents, ethical approval) for 15 years after the end of the study.

To meet regulatory requirements, the CRF data will be electronically stored at sites. The Clinical Data Interchange Standards Consortium (CDISC) Operational Data Modeling (ODM) standard (see www.cdisc.org for details) will be used to store and archive all electronic data at the sites. Since CDISC ODM is also the source for the EDC web-based system, no transcription of data is necessary.

CDISC ODM is a platform-independent, standardized data format which includes the complete study metadata and audit trail. The data can be reviewed at a later stage using off-the-shelf tools. CDISC provides a complete CDISC ODM viewer for these purposes. If needed, hardcopies can be created from ODM files.

14. Monitoring of the Study

The monitor is responsible for obtaining an overview of the course of the trial in co-operation with the investigator, checking if the trial protocol is being observed, and helping the investigators to solve any problems which may arise. All documents in the context with this clinical trial will be handled confidentially at any time. This will be done under preservation of data protection.

The investigator has agreed to give the monitor access to relevant hospital or clinical records to confirm their consistency with the CRF entries and to obtain an adequate overview of the course of the trial. The monitor checks entries in the CRF for completeness, accuracy and correctness. The entries in the CRF will be verified against source documents with respect to the following criteria and in the following frequencies:

- Informed consent 100%
- Demographic data 100%
- In-/exclusion criteria 100%
- Administration of study medication 100%
- AEs 100%
- Time points of visits 100%
- Laboratory parameters 100%
- Vital signs and body weight 100%

The items for source data verification will be specified in detail in the monitoring manual. For data that are directly captured in the CRF (Source), the CRF itself becomes source data and source data verification is not applicable.

The source data verification must be performed by direct insight. If a subject refuses to consent to this procedure he/she must not be enrolled in the study. The investigational site will provide direct access to all trial related sites, source data/documents, and reports for the purpose of monitoring and auditing by the sponsor, and inspection by local and regulatory authorities. The investigator (or a representative) has further agreed to support the monitor in solving any problems he/she discovers during his/her visits.

15. Responsibilities of the Investigator

The investigator agrees to carry out the study in accordance with the guidelines and procedures outlined in this trial protocol. The investigator especially consents to strictly adhere to the ethical principles (see Section 11 of this protocol).

The investigator knows that he/she must, according to professional regulations for physicians, obtain the approval of the competent Ethics Committee prior to enrollment of any study subjects.

Any deviation from the trial protocol must, before its implementation, be agreed to by the sponsor in writing and by the Ethics Committee initially consulted.

Changes to the protocol require written "Amendments to the protocol" and written approval by the Principal Investigator. Changes are allowed only if study value is not reduced and if they are ethically justifiable. The statistician must agree to the amendment, if appropriate, his statement is to be submitted to the Ethics Committee. The amendment must be passed on to all participating investigators with the obligation to adhere to its provisions. If warranted, the Subject Information has to be changed accordingly.

It is within the responsibility of the investigator that an electronic CRF be completed and electronically signed after the subject has finished the trial for each subject participating in the study.

At the conclusion of the study, the investigator will return all partly used, unused and empty drug containers to the sponsor.

The investigator may ask to terminate the study due to administrative or other reasons. If this should be the case, appropriate measures which safeguard the interests of the participating subjects must be taken after verification and consultation with the PI.

The PI is responsible to ensure that all study procedures are performed by appropriately licensed study site staff. The responsibilities of and tasks performed by these individuals need to be documented and filed in the study file and must also be in compliance with any local IRB and/or medical society regulations concerning duties of study site staff.

By signing this protocol, the investigator confirms that he/she has read the entire trial protocol, agrees to its procedures, and will comply strictly with the formulated guidelines.

Each investigator will maintain appropriate medical and research records for this trial, in compliance with ICH E6 GCP, Section 4.9, and regulatory and institutional requirements for the protection of confidentiality of subjects. He/she will permit authorized representatives of the sponsor and regulatory agencies to review (and, when required by applicable law, to copy) clinical records for the purposes of quality assurance reviews, audits, and evaluation of the study safety and progress.

All manuscripts resulting from this trial will be reviewed by representatives from the site. The investigator agrees to follow the detailed publication policy included in the clinical trial agreement.

16. References

ACAM 2000 Vaccines and Related Biological Products Advisory Committee (VRBPAC) Briefing Document, April 2007 http://www.fda.gov/ohrms/dockets/ac/07/briefing/2007-4292B2-00-index.htm

Antoine, G., Scheiflinger, F., Dorner, F., & Falkner, F. G. 1998, "The complete genomic sequence of the modified vaccinia Ankara strain: comparison with other orthopoxviruses", Virology, vol. 244, no. 2, pp. 365-396.

Blanchard, T. J., Alcami, A., Andrea, P., & Smith, G. L. 1998, "Modified vaccinia virus Ankara undergoes limited replication in human cells and lacks several immunomodulatory proteins: implications for use as a human vaccine", J.Gen.Virol., vol. 79 (Pt 5), pp. 1159-1167.

Carroll, M. W. & Moss, B. 1997, "Host range and cytopathogenicity of the highly attenuated MVA strain of vaccinia virus: propagation and generation of recombinant viruses in a nonhuman mammalian cell line", Virology, vol. 238, no. 2, pp. 198-211.

Chan I. 1998, Exact tests of equivalence and efficacy with a non-zero lower bound for comparative studies. Statistics in Medicine 17, 1403-1413.

Chaplin, P. J., Howley, P., & Meisinger, C. 2002, Modified Vaccinia Ankara Virus Variant, WO 02/42480 A2 (patent).

Drexler, I., Heller, K., Wahren, B., Erfle, V., & Sutter, G. 1998, "Highly attenuated modified vaccinia virus Ankara replicates in baby hamster kidney cells, a potential host for virus propagation, but not in various human transformed and primary cells", J.Gen.Virol., vol. 79 (Pt 2), pp. 347-352.

Frey, S. E., Newman, F. K., Cruz, J., Shelton, W. B., Tennant, J. M., Polach, T., Rothman, A. L., Kennedy, J. S., Wolff, M., Belshe, R. B., & Ennis, F. A. 2002, "Dose-related effects of smallpox vaccine", N.Engl.J.Med., vol. 346, no. 17, pp. 1275-1280.

Frey, S. E., Newman, F. K., Kennedy, J. S., Sobek, V., Ennis, F. A., Hill, H., Yan, L. K., Chaplin, P., Vollmar, J., Chaitman, B. R., & Belshe, R. B. 2007, "Clinical and immunologic responses to multiple doses of IMVAMUNE (Modified Vaccinia Ankara) followed by Dryvax challenge", Vaccine, vol. 25, no. 51, pp. 8562-8573.

Goldstein, J. A., Neff, J. M., Lane, J. M., & Koplan, J. P. 1975, "Smallpox vaccination reactions, prophylaxis, and therapy of complications", Pediatrics, vol. 55, no. 3, pp. 342-347.

Grabenstein, J. D. & Winkenwerder, W., Jr. 2003, "US military smallpox vaccination program experience", JAMA, vol. 289, no. 24, pp. 3278-3282.

Gruver AL, Hudson LL, Sempowski GD. 2007 Immunosenescence of ageing. J Pathol. Jan;211(2):144-56.

Guillaume, J. C., Saiag, P., Wechsler, J., Lescs, M. C., & Roujeau, J. C. 1991, "Vaccinia from recombinant virus expressing HIV genes", Lancet, vol. 337, no. 8748, pp. 1034-1035.

Lane, J. M., Ruben, F. L., Neff, J. M., & Millar, J. D. 1969, "Complications of smallpox vaccination, 1968", N.Engl.J.Med., vol. 281, no. 22, pp. 1201-1208.

Lane, J. M., Ruben, F. L., Neff, J. M., & Millar, J. D. 1970, "Complications of smallpox vaccination, 1968: results of ten statewide surveys", J.Infect.Dis., vol. 122, no. 4, pp. 303-309.
McElwain, W. P. 1972, "Complications of smallpox vaccination", J.Ky.Med.Assoc., vol. 70, no. 3, pp. 165-166.

Meyer, H., Sutter, G., & Mayr, A. 1991, "Mapping of deletions in the genome of the highly attenuated vaccinia virus MVA and their influence on virulence", J.Gen.Virol., vol. 72 (Pt 5), pp. 1031-1038.

Pfister G. and Saviano W. 2008 . Can the immune system still be efficient in the elderly? An immunological and immunoendocrine therapeutic perspective.; Neuroimmunomodulation 15(4-6):351-64.

Redfield, R. R., Wright, D. C., James, W. D., Jones, T. S., Brown, C., & Burke, D. S. 1987, "Disseminated vaccinia in a military recruit with human immunodeficiency virus (HIV) disease", N.Engl.J.Med, vol. 316, no. 11, pp. 673-676.

Rosel, J. L., Earl, P. L., Weir, J. P., & Moss, B. 1986, "Conserved TAAATG sequence at the transcriptional and translational initiation sites of vaccinia virus late genes deduced by structural and functional analysis of the HindIII H genome fragment", J.Virol., vol. 60, no. 2, pp. 436-449.
Rosenthal, S. R., Merchlinsky, M., Kleppinger, C., & Goldenthal, K. L. 2001, "Developing new smallpox vaccines", Emerg.Infect.Dis., vol. 7, no. 6, pp. 920-926.

Stittelaar, K. J., van, A. G., Kondova, I., Kuiken, T., van Lavieren, R. F., Pistoor, F. H., Niesters, H. G., van, D. G., van der Zeijst, B. A., Mateo, L., Chaplin, P. J., & Osterhaus, A. D. 2005, "Modified vaccinia virus Ankara protects macaques against respiratory challenge with monkeypox virus", J.Virol., vol. 79, no. 12, pp. 7845-7851.

Sutter, G. & Moss, B. 1992, "Nonreplicating vaccinia vector efficiently expresses recombinant genes", Proc.Natl.Acad.Sci.U.S.A, vol. 89, no. 22, pp. 10847-10851.

Vollmar, J., Arndtz, N., Eckl, K. M., Thomsen, T., Petzold, B., Mateo, L., Schlereth, B., Handley, A., King, L., Hulsemann, V., Tzatzaris, M., Merkl, K., Wulff, N., & Chaplin, P. 2006, "Safety and immunogenicity of IMVAMUNE, a promising candidate as a third generation smallpox vaccine", Vaccine, vol. 24, no. 12, pp. 2065-2070.

17. Appendices

17.1 Appendix I: Toxicity Scale

Grade 1 or Grade 2 toxicity is only graded according to these tables, if the value is outside of the institutional normal range applicable for this study.

Estimating severity grade

For abnormalities NOT found elsewhere in the Toxicity Tables use the scale below to estimate grade of severity:

Grade 1 An AE which is easily tolerated by the subject, causing minimal discomfort and not interfering with daily activities.

Grade 2 An AE which is sufficiently discomforting to interfere with daily activities.

Grade 3 An AE which prevents daily activities. Such an AE would, for example, prevent attendance at work and would necessitate the administration of corrective therapy.

Grade 4 Life-threatening or disabling

Serious or life-threatening AEs

ANY clinical event deemed by the clinician to be serious or life-threatening should be considered a Grade 4 event. Clinical events considered to be serious or life-threatening include, but are not limited to: Seizures, coma, tetany, diabetic ketoacidosis, disseminated intravascular coagulation, diffuse petechiae, paralysis, acute psychosis, severe depression.

Table 7: Serum Chemistry

Lab value	Mild (Grade 1)	Moderate (Grade 2)	Severe (Grade 3)	Potentially Life Threatening (Grade 4)
Sodium – Hyponatremia mmol/L	< Lower Limit of Normal (LLN) - 132	130 - 131	125 - 129	< 125
Sodium – Hypernatremia mmol/L	> ULN - 149	150 - 154	155 - 159	≥ 160
Potassium – Hyperkalemia mmol/L	> ULN - 5.9	6.0 - 6.5	6.6 - 7.0	> 7.0
Potassium – Hypokalemia mmol/L	< LLN - 3.1	2.5 - 3.0	2.0 - 2.4	< 2.0
Calcium – Hypercalcaemia mmol/L	> ULN - 2.89	2.90 - 3.09	3.10 - 3.30	> 3.30
Calcium- Hypocalcaemia mmol/L	< LLN - 2.00	1.76 - 2.00	1.50 -1.75	< 1.50

Serum creatinine mg/dl	≥ ULN -< 1.5 x ULN	≥ 1.5 - < 3 x ULN	≥ 3- 6 x ULN	> 6 x ULN
Alkaline Phosphatase increase by factor	> 1.25 - < 2.0 x ULN	≥ 2.0 - < 3.0 x ULN	≥ 3.0 x ULN	
Liver Function Tests increase by factor	> 1.0 - < 2.5 x ULN	≥ 2.5 - < 4 x ULN	≥ 4 x ULN	
Total Bilirubin increase by factor	> ULN – 1.5 x ULN	> 1.5 - 3.0 x ULN	> 3.0 - 10.0 x ULN	> 10.0 x ULN
Creatine Kinase increase by factor	>ULN - < 2 x ULN	≥ 2 - <5x ULN	≥ 5x ULN	
Creatine Kinase Myocardial Band increase by factor	>ULN – <2.0 x ULN	≥ 2.0 - < 5.0 x ULN	≥ 5.0 x ULN	
Cardiac troponin I increase by factor	>ULN – <2.0 x ULN	≥ 2.0 - < 5.0 x ULN	≥ 5.0 x ULN	
Total Cholesterol mg/dl	> ULN - 300	> 300 - 400	> 400	

Table 8: Hematology

Lab Value	Mild (Grade 1)	Moderate (Grade 2)	Severe (Grade 3)	Potentially Life Threatening (Grade 4)
Hemoglobin (Female) g/dl	< LLN - ≥ 10.5	< 10.5 - ≥ 10.0	< 10.0	
Hemoglobin (Male) g/dl	< LLN - ≥ 12.5	< 12.5 - ≥ 11.0	< 11.0	
WBC Increase cell/mm^3	≥ ULN - < 15.000	≥ 15.000 - < 20.000	≥ 20.000	
WBC Decrease cell/mm^3	< LLN - ≥ 2.500	< 2.500 - ≥ 1,500	< 1.500	
Lymphocytes Decrease cell/mm^3	< 1.000 - ≥ 750	< 750 - ≥ 500	< 500	
Neutrophils Decrease cell/mm^3	< 2.000 - ≥ 1.500	< 1.500 - ≥ 1.000	< 1.000	
Platelets Decreased cell/mm^3	< LLN - ≥ 75.000	< 75.000 - ≥ 50.000	< 50.000	

Table 9: Urine

Protein	Trace - ≤ 1+	> 1+ - ≤ 2+	> 2+ - ≤ 3+	> 3+ - nephritic syndrome
Glucose	Trace	1+	2+	> 2+
Blood (microscopic) red blood cells per high power field (rbc/hpf)	≥ 0 - < 10	≥ 10 - < 50	≥ 50	Gross
Blood (measured by e.g. Combur urine sticks)	Trace ≤ -1+	> 1+ - ≤ 2+	> 2+ - ≤ 3+	≥ 3+

17.2 Appendix II: Case Definitions Acute Myocarditis / Pericarditis

Case Definition for Acute Myocarditis
A possible case of acute myocarditis is defined by the following criteria and the absence of evidence of any other likely cause of symptoms:
Presence of dyspnea, palpitations, or chest pain of probable cardiac origin in a subject with either one of the following:

- ECG abnormalities beyond normal variants, not documented previously, including
- ST-segment or T-wave abnormalities,
- Paroxysmal or sustained atrial or ventricular arrhythmias,
- AV nodal conduction delays or intraventricular conduction defects, or
- Continuous ambulatory electrocardiographic monitoring that detects frequent atrial or ventricular ectopy, **or**
- Evidence of focal or diffuse depressed left-ventricular (LV) function of indeterminate age identified by an imaging study (e.g., echocardiography or radionuclide ventriculography).

A probable case of acute myocarditis, in addition to the above symptoms and in the absence of evidence of any other likely cause of symptoms, has one of the following:

- Elevated cardiac enzymes, specifically, abnormal levels of cardiac troponin I, troponin T, or creatine kinase myocardial band (a troponin test is preferred);
- Evidence of focal or diffuse depressed LV function identified by an imaging study (e.g., echocardiography or radionuclide ventriculography) that is documented to be of new onset or of increased degree of severity (in the absence of a previous study, findings of depressed LV function are considered of new onset if, on follow-up studies, these findings resolve, improve, or worsen); or
- Abnormal result of cardiac radionuclide imaging (e.g., cardiac MRI with gadolinium or gallium-67 imaging) indicating myocardial inflammation.

A case of acute myocarditis is confirmed if histopathologic evidence of myocardial inflammation is found at endomyocardial biopsy or autopsy.

Case Definition for Acute Pericarditis
A possible case of acute pericarditis is defined by the presence of

- Typical chest pain (i.e., pain made worse by lying down and relieved by sitting up and/or leaning forward) and no evidence of any other likely cause of such chest pain.

A probable case of acute pericarditis is a possible case of pericarditis, or a case in a person with pleuritic or other chest pain not characteristic of any other disease, that, in addition, has one or more of the following:

- Pericardial rub, an auscultatory sign with one to three components per beat,
- ECG with diffuse ST-segment elevations or PR depressions without reciprocal ST depressions that are not previously documented, or
- Echocardiogram indicating the presence of an abnormal collection of pericardial fluid (e.g., anterior and posterior pericardial effusion or a large posterior pericardial effusion alone).

A case of acute pericarditis is confirmed if histopathologic evidence of pericardial inflammation is evident from pericardial tissue obtained at surgery or autopsy.

17.3 Appendix III: Test of understanding

Test of Understanding

Protocol Title: A randomized, double-blind, placebo-controlled Phase II study to evaluate safety and immunogenicity of one and two doses of IMVAMUNE® smallpox vaccine in 56-80 year old vaccinia-experienced subjects

Protocol Number: POX-MVA-024, DMID 08-0019

Sponsor: Bavarian Nordic GmbH

Please complete the following questionnaire. Circle only one answer for each question using the true or false responses.

You may retake this test two more times (a total of three times), if you did not answer 9 of the 10 questions correctly on the first attempt.

No.	Statement	True	False
1	I will receive two injections as part of this study	T	F
2	If I have any questions about the study even after I am vaccinated, I can call the study site and they will answer my questions.	T	F
3	This vaccine will give me smallpox.	T	F
4	I may take other experimental products while I am participating in this study.	T	F
5	This vaccine has not previously been studied in humans.	T	F
6	If I have a reaction or problem after having received the vaccine, I must contact the study team.	T	F
7	I should tell the study team if I start any new medication.	T	F
8	I may withdraw from the study at any time if I choose to, or my participation can be stopped if the study team thinks it is in my best interest.	T	F
9	My participation in the study is expected to be 39 weeks.	T	F
10	There are possible risks from getting the vaccine.	T	F

Volunteer's Signature: ____________________________Date: ___________________

Volunteer's Printed Name: ________________________ Subject number: _________

Score: (9 of the 10 questions must be answered correctly)

First attempt: ________% Second attempt: ________% Third attempt: __________%

Study staff (printed name):__________________________Study Site Number ____

POX-MVA-024 Test of Understanding Page 1 of 1 Version 1.0, 18-MAR-2009
