# Supplementary material for: A Randomized, Double-Blind, Placebo-Controlled Phase II Trial Investigating the Safety and Immunogenicity of Modified Vaccinia Ankara Smallpox Vaccine (MVA-BN®) in 56-80-Year-Old Subjects
Source: PLoS One. 2016 Jun 21;11(6):e0157335. doi: 10.1371/journal.pone.0157335 (PMC4915701; doi:10.1371/journal.pone.0157335)
Supplement: S4 File — (PDF) [file pone.0157335.s006.pdf]

A Clinical Phase II Study Confirming the Safety and Immunogenicity of One or Two Doses IMVAMUNE® (MVA-BN®) Smallpox Vaccine in Vaccinia-experienced Elderly Subjects

Greenberg RN[1], Hay CM[2], Stapleton JT[3], Marbury T[4], Wagner E[5], Rösch S[5], Müller J[5], Kreusel C[5], Young P[5], Chaplin P[5]

[1]University of Kentucky, Lexington KY [2]University of Iowa, Iowa City IA [3]University of Rochester, Rochester NY [4]Orlando Clinical Research Center, Orlando FL [5]Bavarian Nordic, Martinsried, Germany

BAVARIAN NORDIC

Abstract

Background

IMVAMUNE® (MVA-BN®) is a live, highly attenuated Modified Vaccinia Ankara virus vaccine formulated at a dose of 1 x 10^8 $TCID_{50}$ / 0.5 ml. IMVAMUNE® does not replicate in human cells and is in clinical development as a 3rd generation smallpox vaccine.

Methods

A Phase II study (POX-MVA-024) was conducted to evaluate safety and immunogenicity (using both ELISA and PRNT) of one and two doses of IMVAMUNE® smallpox vaccine in 56-80 year old vaccinia-experienced subjects (n= 120). Subjects received either two injections of 0.5 ml IMVAMUNE® or one injection of 0.5 ml placebo and one injection of IMVAMUNE® four weeks apart.

Results

Vaccinations were well tolerated by all subjects. No serious adverse events related to IMVAMUNE® and no cases of myo-/pericarditis were reported. The overall incidence of unsolicited AEs was similar in both groups. A second dose did not increase reactogenicity. ELISA as well as PRNT results were comparable after one dose for the two groups.

Group (individual peak titers)	N	ELISA Seroconversion Rate (%)	ELISA GMT	PRNT Seroconversion Rate (%)	PRNT GMT
2 doses IMVAMUNE®	61	90	992	95	258
1 dose IMVAMUNE®	58	85	645	78	140

Response rates and seroconversion rates measured by PRNT increased after a second dose. A second dose increases GMTs in the ELISA and to a higher extent in the PRNT.

Conclusions

One or two doses of IMVAMUNE® were safe and immunogenic in the 56-80 year old vaccinia-experienced population. Safety, reactogenicity and immune responses were similar to that seen in the younger (18-55 year old) healthy population as investigated in other trials. The results indicate that in an emergency situation it is sufficient to vaccinate this population only once.

Methods

This randomized, double-blind, placebo-controlled Phase II trial conducted at four sites in the US enrolled 120 subjects divided among two groups. Vaccinia-experienced women and men aged 56 to 80 years were eligible. The study consisted of a screening period of up to four weeks, an active study period of eight to 10 weeks consisting of five visits, and a follow-up period at least 26 weeks after the last vaccination.

Vaccine Dose and Schedule

Group 1 (N=61) received two subcutaneous (s.c.) vaccinations with IMVAMUNE® (0.5 ml vaccine containing 1 x 10^8 tissue culture infectious dose 50% ($TCID_{50}$)/dose) at 0 and 4 weeks.

Group 2 (N=58) received a first s.c. vaccination with placebo (0.5 ml saline), followed by a second s.c. vaccination with IMVAMUNE® four weeks later.

Safety

To evaluate safety of the IMVAMUNE® vaccinations, solicited and unsolicited adverse events (AEs) were recorded and safety laboratory tests including troponin I, physical examinations including vital signs and electrocardiograms (ECG) were performed.

Criteria for evaluation:

- Serious adverse events (SAEs) associated with the study vaccine
- Unsolicited non-serious AEs within 28 days after each vaccination
- Grade 3 or 4 AEs associated with the study vaccine within 28 days after each vaccination
- Any cardiac events and/or any ECG change indicating a case of myo-/pericarditis
- Solicited local adverse reactions within one week (Days 0 to 7) after each vaccination
- Solicted general AEs within one week (Days 0 to 7) after each vaccination

Immunogenicity

Methods

Immune analyses were performed for each trial visit except for the screening visit. The baseline assessment for immunogenicity parameters was performed prior to the first vaccination. The humoral immune response was tested at each visit using a direct vaccinia-specific ELISA and a vaccinia-specific PRNT.

Criteria for evaluation of ELISA & PRNT:

- Percentage of subjects with responses
- Percentage of subjects seroconverting defined as either the appearance of antibody titers for initially seronegative subjects or a doubling or more of the antibody titer compared to the baseline titer for subjects with a pre-existing antibody titer
- Geometric mean titers (GMTs)

Results

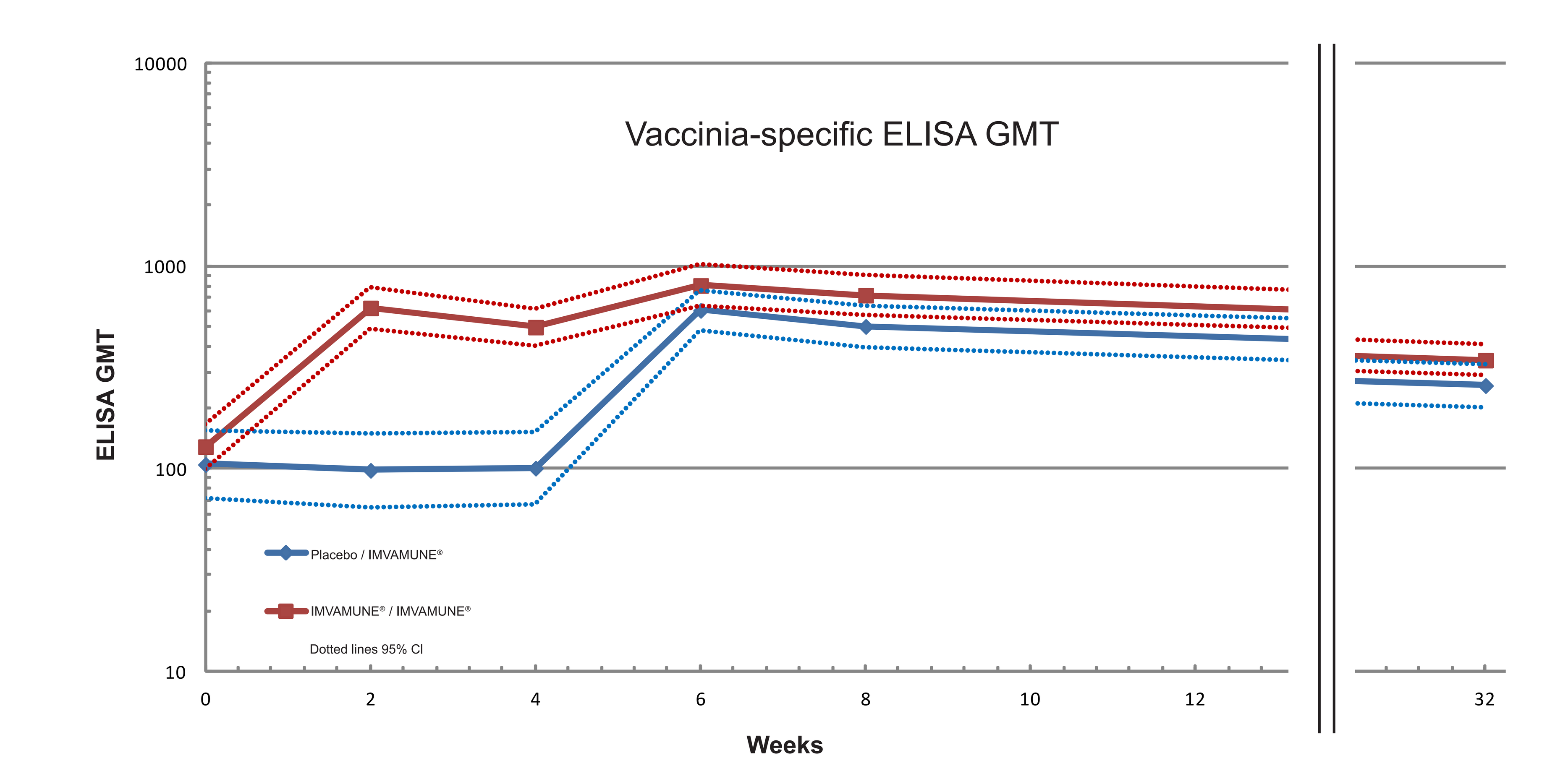

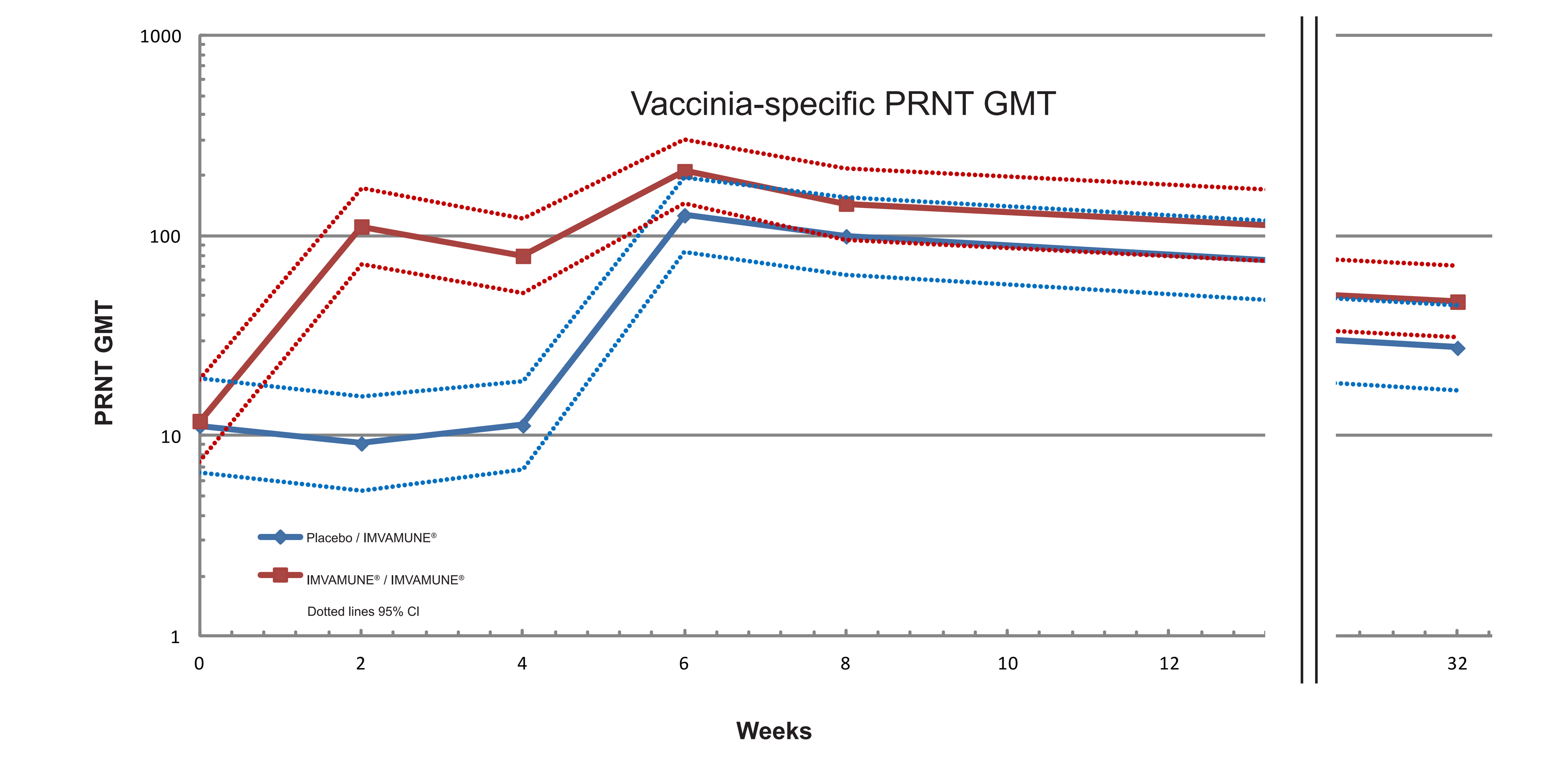

- IMVAMUNE® is immunogenic in a vaccinia-experienced elderly population up to 80 years of age and able to activate immunologic memory
- ELISA as well as PRNT response rates, seroconversion rates, and GMTs were comparable for the two groups after one vaccination with IMVAMUNE® (not all data shown)
- One or two doses of IMVAMUNE® lead to comparable response rates and seroconversion rates measured by ELISA. Response rates and seroconversion rates measured by PRNT increased after a second IMVAMUNE® dose
- A second IMVAMUNE® dose increases GMTs in the ELISA and to a higher extent in the PRNT. Further vaccinia exposure seems to enhance maturation of neutralizing antibodies

Demographics

There were no clinically relevant differences observed in any demographic parameter. In both groups, more female than male subjects were included.

		IMVAMUNE® / IMVAMUNE® (N = 62)	PLACEBO / IMVAMUNE® (N = 58)
Age [years]	Mean	64.6	62.6
	95% Confidence Interval	63.3; 66.0	61.1; 64.1
Gender [n (%)]	Female	37 (59.7)	40 (69.0)
	Male	25 (40.3)	18 (31.0)
Race [n (%)]	White (Caucasian)	59 (95.2)	57 (98.3)
	Black or AA	2 (3.2)	1 (1.7)
	Asian	1 (1.6)	0 (0.0)

Safety Results

Summary of Safety Results

Safety Endpoint	IMVAMUNE® / IMVAMUNE® (N = 62) n (%)	IMVAMUNE® / IMVAMUNE® (N = 62) n (%) related	PLACEBO / IMVAMUNE® (N = 58) n (%)	PLACEBO / IMVAMUNE® (N = 58) n (%) related
At least one AE	57 (91.9)	34 (54.8)	55 (94.8)	33 (56.9)
SAE	2 (3.2)	0 (0.0)	2 (3.4)	0 (0.0)
AEs of Grade ≥ 3	11 (17.7)	3 (4.8)	4 (6.9)	1 (1.7)
Unsolicited AEs vaccination period 1	25 (40.3)	9 (14.5)	18 (31.0)	12 (20.7)
Unsolicited AEs vaccination period 2	23 (41.1)	11 (19.6)	23 (39.7)	13 (22.4)

Summary of Reactogenicity Results

Safety Endpoint: Solicited AEs	IMVAMUNE® / IMVAMUNE® (N = 62) n (%)	IMVAMUNE® / IMVAMUNE® (N = 62) Grade 3	PLACEBO / IMVAMUNE® (N = 58) n (%)	PLACEBO / IMVAMUNE® (N = 58) Grade 3
Solicited local AE (vacc. period 1)	53 (85.5)	4 (6.5)	12 (20.7)	0 (0.0)
Solicited local AE vaccination period 2	45 (80.4)	4 (7.1)	46 (79.3)	2 (3.4)
Solicited general AEs vaccination period 1	27 (43.5)	3 (4.8)	15 (25.9)	0 (0.0)
Solicited general AEs vaccination period 2	21 (37.5)	1 (1.8)	25 (43.1)	1 (1.7)

- No SAE related to IMVAMUNE® was observed
- No cardiac adverse event related to IMVAMUNE® was detected
- Overall few AEs grade ≥3 related to IMVAMUNE® were observed
- The overall incidence of unsolicited AEs was similar in both study groups
- A second dose of IMVAMUNE® administered to this population is safe and reactogenicity does not increase

Conclusions

1. One or two doses of IMVAMUNE® were safe and immunogenic in 56-80 year old vaccinia-experienced subjects
2. Safety, reactogenicity and immune responses were similar to that seen in the younger (18 -55 year old) population as investigated in other trials
3. The results indicate that in an emergency situation it is sufficient to vaccinate this population only once with IMVAMUNE®

Immunogenicity in Young Vaccinia-naïve Subjects

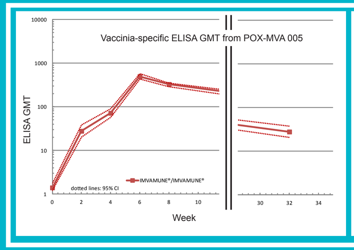

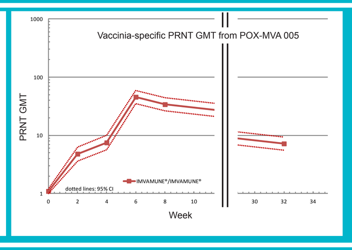

The two graphs show the ELISA and PRNT GMTs from a vaccinia-naïve population (mean age 25.3 years) after two vaccinations with 1x10^8 $TCID_{50}$ IMVAMUNE® at week 0 and week 4. Data are taken from the clinical trial POX-MVA-005.

Corresponding author: Greenberg RN, Rngree01@uky.edu,
University of Kentucky, Lexington KY, USA

Acknowledgements
BN's clinical development program is supported by contract DMID N01-AI-40072
