# Supplementary material for: A Randomized, Double-Blind, Placebo-Controlled Phase II Trial Investigating the Safety and Immunogenicity of Modified Vaccinia Ankara Smallpox Vaccine (MVA-BN®) in 56-80-Year-Old Subjects
Source: PLoS One. 2016 Jun 21;11(6):e0157335. doi: 10.1371/journal.pone.0157335 (PMC4915701; doi:10.1371/journal.pone.0157335)
Supplement: S1 Table — (DOCX) [file pone.0157335.s007.docx]

S1 Table Demographic Data (PPS, N = 102)

|  |  | Group MM (N = 50) | Group PM (N = 52) |
| --- | --- | --- | --- |
| Age [years] | Mean (SD) | 64.4 (5.6) | 62.3 (5.9) |
|  | 95% CI | (62.8, 66.0) | (60.6, 63.9) |
|  | Median | 63.5 | 61.0 |
|  | Range | 56-77 | 56-80 |
| Gender [n (%)] | Female | 30 (60) | 38 (73.1) |
|  | Male | 20 (40) | 14 (26.9) |
| Race [n (%)] | White (Caucasian) | 48 (96.0) | 51 (98.1) |
|  | Black or African American | 2 (4.0) | 1 (1.9) |
|  | Asian | 0 (0.0) | 0 (0.0) |
| Ethnicity [n (%)] | Hispanic or Latino | 1 (2.0) | 0 (0.0) |
|  | Non-Hispanic or Latino | 49 (98.0) | 52 (100.0) |

PPS = Per Protocol Set; N= Number of subjects; n = Number of subjects in specified group;
SD = Standard Deviation
