# Supplementary material for: A Randomized, Double-Blind, Placebo-Controlled Phase II Trial Investigating the Safety and Immunogenicity of Modified Vaccinia Ankara Smallpox Vaccine (MVA-BN®) in 56-80-Year-Old Subjects
Source: PLoS One. 2016 Jun 21;11(6):e0157335. doi: 10.1371/journal.pone.0157335 (PMC4915701; doi:10.1371/journal.pone.0157335)
Supplement: S2 Table — (DOCX) [file pone.0157335.s008.docx]

S2 Table Overview of Immunogenicity Results (PPS, N = 102)

| ELISA | | Baseline | Two weeks after first MVA-BN vaccination ^1^ | Two weeks after second MVA-BN vaccination ^2^ | Individual peak |
| --- | --- | --- | --- | --- | --- |
| Group MM  (N = 50) | Seropositivity rate (%) | 100.0 | NA | NA | NA |
|  | Response rate (%) | NA | 98.0 | 98.0 | 100.0 |
|  | SC rate (%) | NA | 84.0 | 90.0 | 92.0 |
|  | GMT | 130.1 | 634.2 | 874.0 | 1061.9 |
| Group PM (N = 52) | Seropositivity rate (%) | 94.2 | NA | NA | NA |
|  | Response rate (%) | NA | 98.1 | NA | 100.0 |
|  | SC rate (%) | NA | 84.6 | NA | 86.5 |
|  | GMT | 106.3 | 604.7 | NA | 647.3 |
| PRNT | | Baseline | Two weeks after first MVA-BN vaccination ^1^ | Two weeks after second MVA-BN vaccination ^2^ | Individual peak |
| Group MM  (N = 50) | Seropositivity rate (%) | 70.0 | NA | NA | NA |
|  | Response rate (%) | NA | 84.0 | 98.0 | 98.0 |
|  | SC rate (%) | NA | 76.0 | 92.0 | 98.0 |
|  | GMT | 11.4 | 113.2 | 229.6 | 283.0 |
| Group PM (N = 52) | Seropositivity rate (%) | 69.2 | NA | NA | NA |
|  | Response rate (%) | NA | 82.7 | NA | 84.6 |
|  | SC rate (%) | NA | 76.9 | NA | 76.9 |
|  | GMT | 12.1 | 129.6 | NA | 142.9 |

ELISA = enzyme-linked immunosorbent assay, PPS = Per Protocol Set, GMT = geometric mean titer, peak = maximum individual titer of Visit 2 to Visit 5, N = Number of subjects in specified group, NA = Not Applicable, PRNT = plaque reduction neutralization test, SC = seroconversion

^1^ = Two weeks after first vaccination is week 2 for Group 1 and week 6 for Group 2.

^2^ = For Group 1 only: two weeks after second vaccination is week 6 for Group 1.
