# Supplementary material for: A Randomized, Double-Blind, Placebo-Controlled Phase II Trial Investigating the Safety and Immunogenicity of Modified Vaccinia Ankara Smallpox Vaccine (MVA-BN®) in 56-80-Year-Old Subjects
Source: PLoS One. 2016 Jun 21;11(6):e0157335. doi: 10.1371/journal.pone.0157335 (PMC4915701; doi:10.1371/journal.pone.0157335)
Supplement: S3 Table — (DOCX) [file pone.0157335.s009.docx]

S 3Table Maximum intensity and relationship to study medication for unsolicited AEs (29-day follow-up periods after administration) (all enrolled subjects, N = 120)

| Intensity | Group MM (N = 62)  n (%) | Group PM (N = 58)  n (%) |
| --- | --- | --- |
| Grade 1 | 61 (82.4) | 51 (86.4) |
| Grade 2 | 9 (12.2) | 7 (11.9) |
| Grade 3 | 4 (5.4) | 1 (1.7) |
| Grade 4 | 0 (0.0) | 0 (0.0) |
| Missing | 0 (0.0) | 0 (0.0) |
| Total | 74 (100.0) | 59 (100.0) |
| Relationship | Group MM (N = 62)  n (%) | Group PM (N = 58)  n (%) |
| None | 33 (44.6) | 27 (45.8) |
| Unlikely | 6 (8.1) | 2 (3.4) |
| Possible | 20 (27.0) | 13 (22.0) |
| Probable | 8 (10.8) | 5 (8.5) |
| Definite | 7 (9.5) | 12 (20.3) |
| Missing | 0 (0.0) | 0 (0.0) |
| Total | 74 (100.0) | 59 (100.0) |

AE = adverse event, N = number of subjects in the specified group with symptom sheets for unsolicited symptoms, n = number of events, % = percentages based on total number of events.

Intensity: Grade 1 = AE easily tolerated by subject, Grade 2 = AE discomforting, Grade 3 = AE prevents normal activities, Grade 4 = life-threatening or disabling (serious adverse event)
