# Supplementary material for: A Randomized, Double-Blind, Placebo-Controlled Phase II Trial Investigating the Safety and Immunogenicity of Modified Vaccinia Ankara Smallpox Vaccine (MVA-BN®) in 56-80-Year-Old Subjects
Source: PLoS One. 2016 Jun 21;11(6):e0157335. doi: 10.1371/journal.pone.0157335 (PMC4915701; doi:10.1371/journal.pone.0157335)
Supplement: S4 Table — (DOCX) [file pone.0157335.s010.docx]

S4 Table: Unsolicited AEs with possibility of vaccine relatedness with an incidence ≥ 3.0% in any group (29-day follow-up period after first administration) (all enrolled subjects, N = 120)

| System organ class, Preferred term, (MedDRA 12.0) | Group MM (N = 62) n (%) | Group PM (N = 58) n (%) |
| --- | --- | --- |
| At least one AE documented | 9 (14.5) | 12 (20.7) |
| General disorders and administration site conditions | 5 (8.1) | 5 (8.6) |
| Injection site haematoma | 0 (0.0) | 3 (5.2) |
| Injection site nodule | 2 (3.2) | 0 (0.0) |
| Investigations | 2 (3.2) | 6 (10.3) |
| Neutrophil count decreased | 0 (0.0) | 3 (5.2) |
| Nervous system disorders | 2 (3.2) | 0 (0.0) |
| Dizziness | 2 (3.2) | 0 (0.0) |
| Musculoskeletal and connective tissue disorders | 2 (3.2) | 0 (0.0) |
| Skin and subcutaneous tissue disorders | 2 (3.2) | 0 (0.0) |

AE = adverse event, MedDRA = Medical Dictionary for Regulatory Activities, N = number of documented vaccination periods in the specified group, n = number of vaccination periods with at least one report of one particular unsolicited symptom, % = percentages based on N.

Note: There may be findings in more than one category.

Note: The cut-off of ≥ 3.0% was used for both system organ classes and preferred terms.
