# Supplementary material for: A Randomized, Double-Blind, Placebo-Controlled Phase II Trial Investigating the Safety and Immunogenicity of Modified Vaccinia Ankara Smallpox Vaccine (MVA-BN®) in 56-80-Year-Old Subjects
Source: PLoS One. 2016 Jun 21;11(6):e0157335. doi: 10.1371/journal.pone.0157335 (PMC4915701; doi:10.1371/journal.pone.0157335)
Supplement: S6 Table — (DOCX) [file pone.0157335.s012.docx]

S6 Table Unsolicited AEs with an incidence ≥ 3.0% in any group (29-day follow-up period after each administration) (all enrolled subjects, N = 120)

| System organ class, Preferred term, MedDRA 12.0) | Group MM (N = 62) n (%) | Group PM (N = 58) n (%) |
| --- | --- | --- |
| At least one AE documented | 36 (58.1) | 31 (53.4) |
| General disorders and administration site conditions | 9 (14.5) | 10 (17.2) |
| Application site haematoma | 0 (0.0) | 3 (5.2) |
| Injection site haematoma | 1 (1.6) | 4 (6.9) |
| Injection site nodule | 4 (6.5) | 2 (3.4) |
| Injection site pain | 2 (3.2) | 0 (0.0) |
| Musculoskeletal and connective tissue disorders | 6 (9.7) | 6 (10.3) |
| Arthralgia | 1 (1.6) | 2 (3.4) |
| Skin and subcutaneous tissue disorders | 6 (9.7) | 0 (0.0) |
| Infections and infestations | 10 (16.1) | 5 (8.6) |
| Nasopharyngitis | 2 (3.2) | 1 (1.7) |
| Sinusitis | 3 (4.8) | 0 (0.0) |
| Upper respiratory tract infection | 2 (3.2) | 2 (3.4) |
| Cardiac disorders | 3 (4.8) | 0 (0.0) |
| Gastrointestinal disorders | 5 (8.1) | 4 (6.9) |
| Diarrhoea | 1 (1.6) | 3 (5.2) |
| Investigations | 7 (11.3) | 8 (13.8) |
| Alanine aminotransferase increased | 2 (3.2) | 1 (1.7) |
| Lymphocyte count decreased | 2 (3.2) | 0 (0.0) |
| Neutrophil count decreased | 1 (1.6) | 4 (6.9) |
| Nervous system disorders | 4 (6.5) | 4 (6.9) |
| Dizziness | 3 (4.8) | 1 (1.7) |
| Respiratory, thoracic and mediastinal disorders | 5 (8.1) | 3 (5.2) |
| Injury, poisoning and procedural complications | 2 (3.2) | 5 (8.6) |
| Contusion | 0 (0.0) | 2 (3.4) |

AE = adverse event, MedDRA = Medical Dictionary for Regulatory Activities, N = number of subjects in the specified group, n = number with at least one report, % = percentages based on N

Note: There may be findings in more than one category.

Note: The cut-off of ≥ 3.0% was used for both system organ classes and preferred terms.
