# Supplementary material for: A Randomized, Double-Blind, Placebo-Controlled Phase II Trial Investigating the Safety and Immunogenicity of Modified Vaccinia Ankara Smallpox Vaccine (MVA-BN®) in 56-80-Year-Old Subjects
Source: PLoS One. 2016 Jun 21;11(6):e0157335. doi: 10.1371/journal.pone.0157335 (PMC4915701; doi:10.1371/journal.pone.0157335)
Supplement: S7 Table — (DOCX) [file pone.0157335.s013.docx]

S7 Table Baseline (Week 0) ELISA and PRNT seropositivity rates (IAS, N = 119)

| Seropositivity | Group MM (N = 61) | | | | | Group PM (N = 58) | | | | |
| --- | --- | --- | --- | --- | --- | --- | --- | --- | --- | --- |
|  | n | S+ | % | 95% LCLa | 95% UCLa | n | S+ | % | 95% LCLa | 95% UCLa |
| ELISA | 61 | 60 | 98.4 | 91.2 | 100.0 | 58 | 55 | 94.8 | 85.6 | 98.9 |
| PRNT | 61 | 44 | 72.1 | 59.2 | 82.9 | 58 | 40 | 69.0 | 55.5 | 80.5 |

a 95% Clopper-Pearson CI, LCL and UCL.

ELISA = enzyme-linked immunosorbent assay, IAS = Immunogenicity Analysis Set, LCL = lower confidence limit, N = number of subjects in specified group, n = number of subjects with data available, S+ = number of seropositive subjects = number of subjects with antibody titers ≥ detection limit, UCL = upper confidence limit.

% = percentage based on n.
