# Supplementary material for: A Randomized, Double-Blind, Placebo-Controlled Phase II Trial Investigating the Safety and Immunogenicity of Modified Vaccinia Ankara Smallpox Vaccine (MVA-BN®) in 56-80-Year-Old Subjects
Source: PLoS One. 2016 Jun 21;11(6):e0157335. doi: 10.1371/journal.pone.0157335 (PMC4915701; doi:10.1371/journal.pone.0157335)
Supplement: S8 Table — (DOCX) [file pone.0157335.s014.docx]

S8 Table Overview of Immunogenicity Results (IAS, N = 119 ^3^)

| ELISA | | Two weeks after first MVA vaccination ^1^ | Two weeks after second MVA vaccination ^2^ | Individual peak |
| --- | --- | --- | --- | --- |
| Group MM (N = 61 ^3^) | Response rate (%) | 98.4 | 98.3 | 100.0 |
|  | SC rate (%) | 83.6 | 83.3 | 90.2 |
|  | GMT | 622.5 | 804.1 | 992.4 |
| Group PM (N = 58) | Response rate (%) | 96.6 | NA | 100.0 |
|  | SC rate (%) | 82.8 | NA | 84.5 |
|  | GMT | 605.8 | NA | 645.2 |
| PRNT | | Two weeks after first MVA vaccination ^1^ | Two weeks after second MVA vaccination ^2^ | Individual peak |
| Group MM (N = 61 ^3^) | Response rate (%) | 83.6 | 96.7 | 96.7 |
|  | SC rate (%) | 73.8 | 90.0 | 95.1 |
|  | GMT | 111.4 | 210.3 | 257.6 |
| Group PM (N = 58) | Response rate (%) | 82.8 | NA | 84.5 |
|  | SC rate (%) | 77.6 | NA | 77.6 |
|  | GMT | 126.7 | NA | 139.6 |

ELISA = enzyme-linked immunosorbent assay, IAS = Immunogenicity Analysis Set, GMT = geometric mean titer, peak = maximum individual titer of Visit 2 (Week 2) to Visit 5 (Week 8), MM = MVA/MVA, N = Number of subjects in specified group, NA = Not Applicable, PM = Placebo/MVA, PRNT = plaque reduction neutralization test, SC = seroconversion

^1^ = Two weeks after first vaccination is week 2 for Group 1 and week 6 for Group 2.

^2^ = For Group 1 only: two weeks after second vaccination is week 6 for Group 1.

^3^ = One subject was excluded from the IAS because baseline results were missing.
